# Systemic safety inequities for people with learning disabilities: a qualitative integrative analysis of the experiences of English health and social care for people with learning disabilities, their families and carers

**DOI:** 10.1186/s12939-021-01612-1

**Published:** 2022-01-28

**Authors:** Lauren Ramsey, Abigail Albutt, Kayley Perfetto, Naomi Quinton, John Baker, Gemma Louch, Jane O’Hara

**Affiliations:** 1 Bradford Institute for Health Research, Bradford Teaching Hospitals NHS Foundation Trust, Bradford, UK; 2 NIHR Yorkshire and Humber Patient Safety Translational Research Centre, Bradford, UK; 3 Bradford Institute for Health Research and NIHR Yorkshire and Humber Patient Safety Translational Research Centre, Bradford, UK; 4 Queen’s University, 9 University Avenue, Kingston, Ontario Canada; 5 Leeds Institute of Medical Education, University of Leeds, Leeds, UK; 6 School of Healthcare, University of Leeds, Leeds, UK; 7 School of Healthcare University of Leeds UK and Theme Lead of Patient Involvement in Patient Safety NIHR Yorkshire and Humber Patient Safety Translational Research Centre, Leeds, UK

**Keywords:** Healthcare inequity, Health services research, Learning disabilities, Intellectual and developmental disabilities, Patient safety, Patient and family involvement, Patient centred care, Patient engagement, Patient experience, Qualitative methods

## Abstract

**Background:**

Failures in care for people with learning disabilities have been repeatedly highlighted and remain an international issue, exemplified by a disparity in premature death due to poor quality and unsafe care. This needs urgent attention. Therefore, the aim of the study was to understand the care experiences of people with learning disabilities, and explore the potential patient safety issues they, their carers and families raised.

**Methods:**

Two data sources exploring the lived experience of care for people with learning disabilities were synthesised using an integrative approach, and explored using reflexive thematic analysis. This comprised two focus groups with a total of 13 people with learning disabilities and supportive staff, and 377 narratives posted publicly via the feedback platform Care Opinion.

**Results:**

The qualitative exploration highlighted three key themes. Firstly, health and social care systems operated with varying levels of rigidity. This contributed to an inability to effectively cater to; complex and individualised care needs, written and verbal communication needs and needs for adequate time and space. Secondly, there were various gaps and traps within systems for this population. This highlighted the importance of care continuity, interoperability and attending to the variation in support provision from professionals. Finally, essential ‘dependency work’ was reliant upon social capital and fulfilled by paid and unpaid caring roles to divergent extents, however, advocacy provided an additional supportive safety net.

**Conclusions:**

A series of safety inequities have been identified for people with learning disabilities, alongside potential protective buffers. These include; access to social support and advocacy, a malleable system able to accommodate for individualised care and communication needs, adequate staffing levels, sufficient learning disabilities expertise within and between care settings, and the interoperability of safety initiatives. In order to attend to the safety inequities for this population, these factors need to be considered at a policy and organisational level, spanning across health and social care systems. Findings have wide ranging implications for those with learning disabilities, their carers and families and health and social care providers, with the potential for international learning more widely.

**Supplementary Information:**

The online version contains supplementary material available at 10.1186/s12939-021-01612-1.

## Background

People with learning disabilities are at an increased risk of preventable harm, avoidable death and reduced life expectancy when compared to the general population in the UK [1, 2], and patient safety issues for this population remain an international concern. Learning disabilities have been defined as “the presence of a significantly reduced ability to understand new or complex information and to learn new skills, with a reduced ability to cope independently which started before adulthood, with a lasting effect on development” [3].

Failings in care have been illuminated in the Confidential Inquiry into Premature Deaths of People with Learning Disabilities (CIPOLD) [4], and the subsequent Learning Disability Mortality Review (LeDeR) programme [5–8]. For instance, a disparity in age of death was identified, at 22 years younger for men and 27 years younger for women, resulting from conditions that could have been prevented with good quality care [8]. The LeDeR mortality reviews also identified three common themes [7]. Firstly, the need for co-ordinated care for people with complex or multiple health conditions, secondly, the effective provision of reasonable adjustments for people with learning disabilities and their families, and thirdly, mandatory awareness training for all staff supporting people with learning disabilities.

Building on this evidence, authors of a recent themed review from the National Institute for Health Research (NIHR) concluded that widespread inconsistencies in the quality and safety of health and social care for people with learning disabilities persist [9]. The review called for improvements in areas including the earlier identification and treatment of health risks, the provision of enhanced community services, assurances that hospital care is safe and effective, and the development of more supportive approaches to care across services [9]. A recently published scoping review also found increased safety risks for a range of hospital outcomes, including adverse events, maternal and infant outcomes and post-operative outcomes [10]. Similarly, key safety vulnerabilities were highlighted in a paediatric setting, increasing the likelihood of children with learning disabilities experiencing poor quality care and iatrogenic harm during their hospital stay, with negative implications for treatment outcomes [11]. Despite the legal interface of reasonable adjustments being in force in the UK since the Equality Act over a decade ago [12], core recommendations resulting from inquiries into substandard care for people with learning disabilities have repeatedly stated that disability must not be used to justify or excuse different standards of care [13–15]. Taken together, this evidence affirms that inequities for people with learning disabilities needs urgent attention.

The aim of this study was to understand the care experiences of people with learning disabilities, their families and carers. To capture these experiences comprehensively, the study was inclusive of a broad range of care settings, including reference to primary, secondary, mental health, tertiary, dental, social and community care in England. Specifically, the following research questions were addressed;

1. How do people with learning disabilities, their carers and families experience care?
2. What are the potential patient safety issues regarding those experiences of care?
3. Are the identified patient safety issues amenable to change in order to provide the basis for future interventions to ensure high quality and safe care for people with learning disabilities?

## Methods

This study adopted an explorative, qualitative design to understand the experiences of care for those with learning disabilities from the perspectives of people with learning disabilities, their families and carers.

### Patient and public involvement and engagement

The study materials were developed in conjunction with the learning disabilities community groups from which participants were recruited. Sara Ryan and Kate Smyth, two people with lived experience of learning and physical disabilities, also supported the development and conduct of this research. Sara has campaigned since the preventable death of her son Connor in 2013, leading to the identification of systematic failings in care for people with learning disabilities and mental ill-health, and helped shape our thinking throughout. Kate has lived experience and expertise in the field of disabilities and has volunteered as a Disability Adviser to train healthcare assistants to improve the way that people with disabilities are cared for in hospital, and helped define the focus of our work and iterated a draft of the paper.

### Data source 1. Focus groups

A total of 13 individuals were recruited via community groups in England to participate in one of two semi-structured focus groups, held within community settings to ensure participant convenience and familiarity. Community groups were identified via existing links with the University of Leeds, and contacting representatives via email. Focus groups were offered as an optional activity for adults with learning disabilities who had the capacity to consent, running alongside other regular sessions, such as arts and cooking. Other stakeholders were also invited to participate and/or support those with learning disabilities during the session where necessary. Focus groups consisted of the group facilitator, people with moderate to severe learning disabilities, supporting staff, and stakeholders in learning disabilities care, including a learning disabilities nurse, a social worker and a local liaison officer for a learning disabilities charity. To avoid unnecessarily burdening participants, other specific demographic information was not collected from individuals, however, this was recognised as a potential limitation.

The first focus group included seven participants, comprising five adults with learning disabilities and two supporting staff members, however, two participants were nonverbal. The second focus group included six participants, comprising three people with learning disabilities and three supporting staff members, all of whom were verbal. A topic guide was developed by authors in conjunction with the community groups to broadly direct the conversation towards experiences of care and potential patient safety issues, and to ask participants to suggest what they would like healthcare staff to know. Participants were also invited to draw upon previous positive and negative care experiences that were not specific to any setting. However, the guide also allowed for novel avenues of conversation to be pursued and views to be expressed freely, in order to maintain the appropriate level of spontaneity considered desirable for high-quality discussion [16, 17]. Prior, during and after the focus groups, the facilitator (NQ) kept field notes to capture nonverbal elements. The focus groups lasted approximately 90 min each.

### Data source 2. Care Opinion feedback (www.careopinion.org.uk)

A purposive sample of 377 feedback narratives publicly available on Care Opinion written by, or about, people with learning disabilities were extracted for analysis. Care Opinion is a national not-for-profit online platform on which the public can provide feedback regarding their care experiences using free text, which can then be responded to by staff within the organisation(s) concerned. This offers a rich, specific and naturally existing data source of discussions between people with learning disabilities, their carers, families and staff, capturing spontaneity. Care Opinion also interoperates with NHS.UK, a similar platform provided by NHS England.

All feedback concerning people with learning disabilities in any care setting in England published between June 1st 2017 and June 1st 2020 was extracted using the filters available. The sample was limited to feedback that had been published within three years of data extraction to ensure relevance to current issues in patient safety. A total of 377 feedback narratives comprised 299 which were uploaded to Care Opinion, and 78 which were uploaded to NHS.UK but subsequently published on Care Opinion. 121 were authored directly by people with learning disabilities, and 256 were authored on the behalf of someone with learning disabilities by representatives such as carers, family, advocates, friends, volunteers, or health professionals. Two feedback narratives used Talking Mats, a tool enabling those with communication difficulties to give their feedback more easily, and six were hand-drawn.

### Data integration and analysis

Focus group audio recordings were transcribed by an in-house transcriber, and Care Opinion feedback was extracted for analysis. To gain multiple and varied perspectives, and explore the research questions in-depth, the two separately generated, yet complementary data sources, were integrated to be analysed together [18, 19]. Together, all authors deemed the two data sources adequate to develop a robust and valid understanding of the study phenomenon, and therefore data collection stopped. Equal weighting was accorded to data sources and data retained their original form, with data brought together to explore similar ideas and considered as a single data set. The study was drafted in accordance with the consolidated criteria for reporting qualitative research (COREQ) [20].

A reflexive thematic analysis approach was used iteratively [21, 22]. Data were read carefully several times to gain a holistic view and achieve immersion by five authors independently (LR, AA, KP, JOH, JB). Researchers have multidisciplinary expertise, with backgrounds in improvement science, patient safety, psychology, mental health, qualitative methods and health services research. Reflective and descriptive comments were made and significant extracts were highlighted, making note of initial impressions, commonalities and differences. Discussion between authors helped inform a provisional inductive coding framework, based upon identified issues and concepts, which was refined collaboratively via regular discussion and cross-referencing with the research questions. Once authors reached consensus, data were systematically coded by three authors (LR, AA, KP), with significant extracts helping to define and evidence each theme, while revisiting original data to ensure that decisions were grounded within the data. Representation of data sources and participants was not necessarily equal, but determined by the relevance and importance within each theme, and the extent they addressed the research questions. A detailed log of theme development, refinement and rationale was kept throughout.

## Results

Our results comprise three core themes, each with their own subthemes. The definition of each theme, how subthemes are interlinked and supporting evidence from the qualitative data are presented.

### Health and care rigidity

Our first theme refers to overriding expectations that people with learning disabilities should fit within existing health and social care systems not necessarily designed with their needs in mind, rather than services adequately adapting to accommodate and ensure safe care. The varying levels of profound and complex needs of people with learning disabilities were often multifaceted, including both physical and mental health symptoms, which were attended to in various settings. As a result, many expressed basic requirements for all staff, regardless of setting, to make reasonable adjustments by having a comprehensive understanding of how people with learning disabilities needed to be cared for, to be able to establish effective communication and to treat people with learning disabilities with dignity, respect and compassion. Areas of particular concern included failures in information exchanges and insufficient time and space provided to people with learning disabilities during care interactions, causing unnecessary distress.

#### Complex and individualised care needs

People with learning disabilities often experienced lifelong conditions which varied in their nature and complexity. Therefore, all stakeholders considered the individualisation of care to be imperative to quality and safety outcomes. Many drew upon negative experiences where care could not be tailored to their specific needs and staff had failed to appropriately adjust support, leaving people feeling unheard, unseen and demoralised.

*“I’d be like, “please these lights”, I actually get headaches that make me have fits… I’ve actually got a phobia of hospital bathrooms, closed spaces. If someone leaves me and locks the door… I freak… I said “can you stay by the door?” and they didn’t, and I just couldn’t get back to the bathroom and I couldn’t walk, I needed help to get back… they thought I was being horrible. I tried to explain to them the reasons why, but they wouldn’t, they didn’t listen… it looks like I’m daydreaming and I’m zoning out for a bit and next minute I’m so scared, people are like, shouting at me in my face telling me to do things and I haven’t got the foggiest what’s happening.” (Focus group 2, participant 3 – person with learning disabilities)*.

The lack of system malleability to cater to complex needs meant that some organisations arguably seemed to come to accept standards of care that would be otherwise deemed unacceptable. This included the management of distress and relief, symptom interactions, physical and mental health concerns, and pain. Similarly, management of what was often termed ‘challenging behaviour’ sometimes meant that people with learning disabilities were considered too difficult to care for in settings without specialised resources, resulting in discharge from services.

*“I was sent here as my last hospital couldn’t handle my challenging behaviour.” (Care Opinion, Independent charity providing women’s psychiatric services - staff posting for patient with learning disabilities)*.

In some cases, physical and mental health symptoms were described as being falsely attributed to direct symptoms of learning disabilities, a phenomenon known as diagnostic overshadowing. The importance of fully exploring and addressing symptoms and their interactions was expressed.

*“I was waiting in A&E to see someone for my depression, I had to wait a good seven odd hours… the biggest issue I had there was waiting all alone with no one to talk to… They don’t really look at how ill I am and they feel I’m just overreacting. I got really upset, I started self-harming and all I remember was this nurse coming through, “excuse me are you going to carry on doing that? I will call the police and have you removed”… that felt even more depressing because it’s like I’m not being understood… When you go to these places, it’s like we’re trusting them with our lives” Focus group 2, participant 5 – person with learning disabilities)*

#### Written and verbal information exchanges

The relational aspects of care were deemed particularly important to ensure that people with learning disabilities felt listened to, and able to understand written and verbal information. Specific considerations were made regarding the accessibility of documents not presented in ‘easy-read’, such as text size, boldness of font, length of information, and the use of imagery to aid understanding.

*“I couldn’t read this massive folder. It’s too many words to take in… bullet points, it’s so easy for people like me to have a bullet point, okay, that makes more sense… She made a care plan and she did it exactly the way I wanted… I chose pictures because it’s more simplified.” (Focus group 2, participant 5 – person with learning disabilities)*.

Risks to patient safety were increased when effective communication could not be established, having wide ranging implications from an inability to read hospital menus, medication information and care documentation, through to omitting opportunities for informed consent.

*“I want it like bigger… I can’t see it, so small … if they give me a piece of paper, I’m like, well you’ll have to explain it or I’m going to have to take it home and show it to my mum and see what she says, because this isn’t right… I can’t understand what I even have to do.” (Focus group 1, participant 7 – person with learning disabilities)*.

Similar considerations were made regarding individualised needs for verbal information to ensure effective three-way communication between people with learning disabilities, their families and/or carers and care professionals. This included details of care being clearly explained, staff having the time to get to know individuals and how they preferred to be communicated with, and confirmation that discourse had been fully understood. Without such efforts in place, staff were sometimes falsely assuming knowledge.

*“He would take his hearing aid out when he was sleeping or resting, and the doctors were talking with him and not realising he couldn’t hear anything… That’s the problem, because the doctor thinks you’ve understood everything you’ve just said, the person goes home… they’re probably back in again.” (Focus group 2, participant 4 – learning disabilities self-advocacy manger)*.

Communication issues present across the general population were also perpetuated for some with learning disabilities, such as language barriers and digital exclusion posing challenges to effective information exchanges. For instance, basic administrative tasks such as providing personal details to services or checking into appointments using technology omitted considerations to present information in ‘easy read’, and some were encouraged to source information important to their health and care via the internet which posed challenges.

*“The problem is, with online, it’s not simplified… when you click a simple question it comes up with various results and you’re thinking, “what, what, what?” it’s nothing related to what you’re typing in.” (Focus group 2, participant 5 – person with learning disabilities)*.

Conversely, others reflected on positive aspects of communication. The significance of staff being approachable, polite, helpful, friendly, professional, and sensitive was repeatedly highlighted. Additionally, the extent staff expressed compassion, empathy, respect and humour, and were perceived to be caring, reassuring and thoughtful were considered imperative. Where information was able to flow effectively, and clear communication could be established, there was a gratefulness for details being thoroughly explained and all stakeholders partnering to ensure high quality and safe care throughout.

*“Our daughter recently moved… her transition was planned very well and the staff were so welcoming, caring… Communication is excellent with the nurses/care workers… We are always told about appointments and updates on how they went, and we feel very much a part of her care still.” (Care Opinion, Nursing home – family of person with learning disabilities)*.

#### Insufficient space and ‘hurry sickness’

Individuals drew upon the need for care providers to move at a pace the individual with learning disabilities could cope with. Feeling rushed within time restricted care interactions was often overwhelming, not only causing unnecessary distress, but also meaning that people could not achieve what they wanted from their appointments. ‘Hurry sickness’, therefore, has the potential to amplify any existing safety inequities and cause iatrogenic harm.

*“I don’t like being rushed, because I feel flustered… sometimes people do rush me… I was in hospital last time, when I said, I can’t speak to you when I have five doctors crowding me all the time.” (Focus group 2, participant 3 - person with learning disabilities)*.

Similarly, the lack of sufficient space was sometimes overwhelming. People with learning disabilities recalled unsettling care experiences where they perceived to have been swarmed by groups of healthcare staff during ward rounds or consultations or were asked to sit in overcrowded waiting rooms.

*“I was taken into a room by a counsellor and was shocked to find another counsellor waiting for us. I was expecting only one-on-one, and this immediately threw me. The assessment was done by both of them together, which was really difficult, because I couldn’t focus on both of them. [I] felt they were ganging up on me.” (Care Opinion, Mental health inpatient services – service user with learning disabilities)*.

Positive reflection from people for whom this reasonable adjustment could be accommodated further exemplified the importance, as people with learning disabilities, their carers and families felt ‘unhurried, cared for and valued’ due to the adequate time and space services and staff were able to provide.

*“They take plenty of time, interact with him really well and save so much unwanted stress.” (Care Opinion, Dental care – staff member posting for a carer)*.

Others also raised novel ideas to alleviate distress, both hypothetically and drawing upon previous personal experiences, such as designated quiet rooms, ‘safe rooms’ and unoccupied areas for those with learning disabilities to wait prior to their appointments.

### Systemic gaps and traps

Our second theme refers to discontinuities in processes and features of health and social care services with the potential to result in poorer safety outcomes, such as errors and unintended adverse events. Significant gaps and traps within the system were highlighted for people with learning disabilities, with commonly reported examples comprising the variation in support within and between services, suboptimal staffing levels, discontinuities in care, and challenges with interoperability.

#### Variation in support provision

Repeated comparisons of support offered within and between health and social care services highlighted a lack of parity. Some raised concerns regarding inequities in support identification and access, as this was heavily reliant upon actively seeking out information or learning via word of mouth. Other examples of best practice were drawn upon, noting where people felt that staff were able to go above and beyond to ensure high quality and safe care, such as those providing specialist support including Learning Disabilities Liaison Nurses, Counsellors and Autism Nurses.

*“I was petrified to go to see a psychologist… So [an autism nurse] actually came with me to my appointment… I am scared of the word Psychology… This is going to sound silly, but I felt like she was safeguarding me… I’m not going by myself. The problem is as well because my health is so complex, that I’m scared they’d try to trip me up.” (Focus group 2, participant 3 – person with learning disabilities)*.

Variation in support provision across services was impacted upon by the adequacy of staffing levels, resulting in the potential to widen gaps and cause patient safety risks. Many noted that suboptimal staffing emphasised known issues, including the discontinuation of scheduled activities and hospital leave, increased waiting times, perceptions of poor staff attitudes and insufficient time spent properly caring for people with learning disabilities. This had impacts ranging from disappointment, increased distress and a sense of unsafe care, to reported potentially life-threatening risks.

*“I put in a complaint 8 weeks ago and again 4 weeks ago because I was left without care and support due to staff leaving and I almost lost my life.” (Care Opinion, Home care, person with learning disabilities).*

Despite the majority focussing on both the want and need for support from services, some did call for increased autonomy, particularly those within an inpatient mental health setting. For instance, some referred to being denied access to personal items and wanting freedom for independent activities, such as internet access and hospital leave.

#### Importance of care continuity and interoperability

Significant benefits were reported where people with learning disabilities had developed continuous relationships within and between services with caring professionals, particularly where staff were able to provide support during transitions. This included the ability to build trust, reduce apprehensions and provide comforting reassurances. Where continuity of care was unable to be established or when care was disrupted, gaps within the system were widened, exacerbating issues with complex health concerns being taken seriously and addressed appropriately, and risking leaving people feeling ‘passed around from pillar to post’. This was emphasised in certain contents, such as being continuously referred to different health professionals, turnover of paid carers, changing of shifts in hospital, and feeling abandoned when being discharged from hospital to home.

*“A familiar face that’s who I need… doctors and nurses keep changing shifts, they’re not familiar and that is scary… They just presume that we’re okay and we can manage but we can’t… they don’t write down what medicines we took when and where and what the medicines are and, most times I’ve had to ring up the ward.“ (Focus group 2, participant 3 – person with learning disabilities)*.

Issues were also raised regarding the ability for healthcare systems to speak to one another, meaning that information had to be repeatedly retold, which was both repetitious for people with learning disabilities, and an additional burden for families and carers.

*“They’re supposed to write to your GP about everything that’s just happened… you finally get a GP appointment and the GP goes “I haven’t got that letter”… I’m sorry to say this, I don’t want to talk about my past all the time… A lot of my care now is in [my local Town], but some is in [the City], but the computers are not compatible’ (Focus group 2, participant 3 – person with learning disabilities)*.

Safety initiatives such as health passports and communication books were designed to minimise risks where interoperability could not be achieved, however, concerns were raised with these being viewed as a temporary measure, rather than looking to resolve larger systemic issues with gaps within and between services. These tools were also variably, and only locally adopted in certain care settings in the absence of a national system, which was perceived as frustrating, and challenges persisted with their use within organisations.

*“Some hospitals wouldn’t accept them [hospital passports] if they originated from a different hospital where essentially it’s just information that’s useful to share.” (Focus group 2, participant 2 – person with learning disabilities)*.

### Dependency work

Our final theme refers to the efforts made by those ‘picking up the slack’, and buffering against potential safety inequities for people with learning disabilities. This role tended to fulfilled by those in family caring roles, with a reliance upon social capital, and/or support from advocates. Therefore, safety inequities may be compounded where there are systemic resource constraints widening safety gaps in care, resulting in increasing demands for dependency work that cannot be easily met.

#### Reliance on social capital

‘Dependency work’ undertaken by individuals outside the official health and social care system was often fulfilled by family members who knew the individual well, and did not see their caring role outside their existing relationship. Those caring relationships were deemed essential and evident in various guises, such as pragmatic support, providing reassurances by alleviating fears of the unknown, bringing a sense of familiarity, and helping people with learning disabilities to feel safe while interacting with clinicians across a variety of care settings.

*“We need some people that we know to sit with us all the time to make sure we’re okay… I’ve got a weird phobia of everything to do with hospitals, so sometimes we just need reassurance.” (Focus group 2, participant 3 - person with learning disabilities)*.

Moreover, interpretive communication support was fulfilled by caring relationships. There was sense that staff often failed to understand and make themselves understood when interacting directly with people with learning disabilities. Therefore, social capital was called upon to bridge gaps in communications. In the absence of social support, however, effectively navigating the system became problematic and posed risks to safety. This was fuelled by fears regarding scenarios where social support would be temporarily or permanently unavailable to people with learning disabilities in the future, such as caring roles being fulfilled by ageing parents.

I have to have medication and the Doctor will, like, tell me and then I’m going, “mum I don’t know what they’ve said, can you explain?”… that’s why I always have to take my mum with me… I don’t know what the doctors are saying… If my mum goes away or something and I’m on my own… I’ll have to wait ‘till my mum gets back for appointments.” (Focus group 1, participant 7 – person with learning disabilities)

While the involvement of family carers was largely welcomed and relied upon by people with learning disabilities, some felt that contributions from paid carers were disempowering, due to exclusion from discussion and decision-making with health professionals suggesting that ‘carers seem to take over’. Nevertheless, where family carers trusted that the system was able to safely and effectively support their relative in their absence, their emotional burden was eased, bringing “complete peace of mind”, knowing that they were being taken good care of. One individual raised an innovative idea of a ‘buddy system’, whereby ‘experts by experience’ could help to support one another in the instance that their social capital was limited or provide welcome respite for unpaid carers at risk of burnout. However, it was unclear if or how this system might effectively work in practice.

#### Safety net of advocacy

Gratitude for additional support from advocates was emphasised by all stakeholders,

particularly when they worked independently of health and social care systems. Advocates were described as effective system navigators, and felt that their role was pivotal for a variety of reasons, including being a dependable lifeline to having voices heard. Sometimes in contrast to care providers, advocates were seen as activists who offered a non-judgemental ear, and worked to ‘fight the corner’ of people with learning disabilities, with specialist knowledge and expertise.

*“I was being pushed into decisions that I had no control over. But after [my advocate] came into the ward rounds with me, I felt a lot calmer and in control. The advocacy is not communicated enough on the ward as a patient’s choice.” (Care Opinion, Independent advocacy support in an acute mental health ward – patient with learning disabilities)*.

Advocacy services offered help with a variety of care elements, such as producing documentation, assisting in organising thoughts around care preferences, discussing personal matters people felt uncomfortable disclosing to friends and family, supporting carers, being present at ward rounds or meetings, identifying needs and ensuring implementation of reasonable adjustments and helping to prevent complaints from being discounted.

*“My health condition makes consistent cognition difficult… Having someone to come with me, whilst I discussed very personal matters was crucial…. [My community advocate] has been informed and understanding about my condition… utterly dependably, available and has produced typed notes after every meeting which has been invaluable. She has also listened accurately to my needs and produced very professional letters and emails to various figures of authority. She has enabled me to challenge mistakes.” (Care Opinion, Independent community advocacy support – patient with learning disabilities)*.

In doing so, many perceived that advocates enabled outcomes to be achieved for those with learning disabilities that would not have been possible otherwise, such as earlier discharge, exercising of rights, challenging staff decisions, and ensuring less restrictive care options were considered.

*“I had issues around leave, medication and discharge, which [my advocate] supported me with by explaining my rights, speaking with ward staff and social worker, challenging decisions, and helping me prepare for ward rounds. I’ve been discharged now and want to thank her for all of her support. Being in hospital has been a very difficult and lonely time and knowing I could call her for information has made a big difference.” (Care Opinion, Independent advocacy support in an acute mental health ward – patient with learning disabilities)*.

The genuine difference that advocacy made to the quality and safety of care for those with learning disabilities alleviated pressures on families who battled to ensure that their relative was not unnecessarily disadvantaged.

*“She did not want to engage with the care team until you became involved. You put her wishes and feelings forward… Having challenged the decision about discharge, she went home with a full package of care. As family members, we would like to thank you as we would not have achieved this result if you had not been at the meeting, you ensured that the least restrictive option was considered.” (Care Opinion, Independent advocacy support – volunteer posting for family of person with learning disabilities)*.

## Discussion

**Fig. 1:**
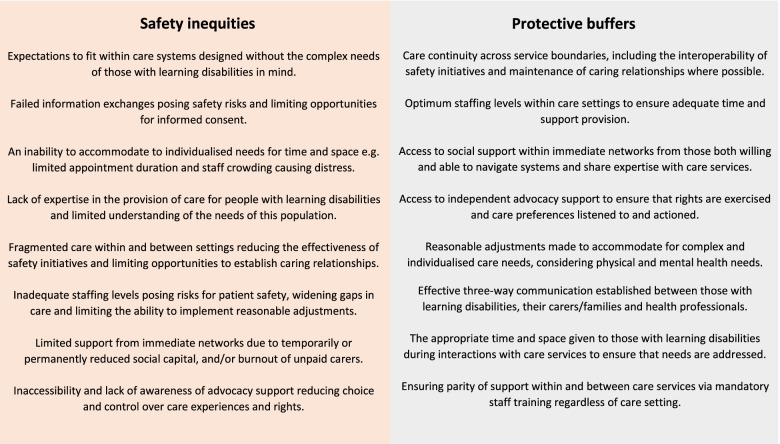
Safety inequities and protective buffers

Our qualitative exploration of experiences of care for those with learning disabilities, their carers and families goes beyond previous research which predominantly aims to learn from deaths in this population [1, 2, 4–8], and explores patient safety issues more widely. Our findings have highlighted both safety inequities and potential protective buffers enabling the optimum environment for high quality and safe care including: access to social support and advocacy, malleability of the system to accommodate individualised care and communication needs, appropriate staffing levels, sufficient expertise within and between care settings, and the interoperability of safety initiatives (summarised in Fig. 1). The safety inequities are likely avoidable and modifiable via quality and safety interventions such as system redesign and reconfiguration, increase in time and resource, staff training, professional specialisation, and modifications of current practice.

Interestingly, the patient safety issues are not necessarily novel to this population. However, importantly, the gaps in care appear to be wider, meaning that there are increased pressures on those outside of the system to ‘step in’ and support effective care. This suggests that healthcare systems are setup in such a way that facilitate safety inequities for people with learning disabilities, that often need to be buffered by themselves, or their social capital. Aligning with theoretical approaches to patient safety more generally, this suggests that patients and families provide a buffer for health and care system safety by ‘scaffolding’ support [23–26], and is cohesive with the early ‘patient-voice’ movement grounded in learning disability activism, campaigning for “no decision about us, without us” [27]. One potential concern is that this issue creates ‘hidden’ groups of people with learning disabilities, who may avoid interactions with health and care services due to the absence of support, and are subsequently at an increased safety risk. Additionally, where support is available, there are risks of burnout for those expected to ‘plug’ safety gaps and take on additional emotional loads while often lacking support themselves. For instance, the National Institute for Health and Care Excellence (NICE) has identified how breakdowns in family support due to ageing carers can create heightened vulnerabilities for those with learning disabilities [28], which has been further demonstrated in children with learning disabilities who are reliant upon parental presence during hospitalisation as a protective factor [11]. As evidenced here, diagnostic overshadowing has also been a longstanding issue, leading to experiences of poor care and potentially avoidable deaths [29–32].

One possible way of helping to address these risk factors is by not only ensuring that each patient with learning disabilities has the option to access support via advocacy, but also, increasing and/or introducing learning disabilities specialists across care settings as previously called for [33]. This specialism would help to ensure the provision of care informed by the appropriate skills and knowledge, and perhaps bridge some of the identified risks to patient safety, such as expectations to fit within care systems designed without those with learning disabilities in mind, failures in information exchanges, limited understanding of patients’ needs, inadequate staffing levels and providing those with learning disabilities with the appropriate time and space during appointments. Learning Disabilities Liaison Nurses for example, have shown to be key enablers to providing reasonably adjusted health services [34].

Previous research has focussed on training staff in caring for those with learning disabilities, including specific areas of concern such as pain recognition and management [35]. However, a randomised controlled trial of staff training focussed on reducing challenge behaviour did not show significant improvements in either primary or secondary outcomes, suggesting that training alone may have limited benefits in practice [36]. Innovatively, a recent training programme has focussed equipping families and carers will skills to better support people with learning disabilities [37], however, this may increase the burden on those outside of the system further.

Worryingly, Covid-19 is arguably further illuminating safety inequities for people with learning disabilities found within this study, and have been alluded to since the early national “Healthcare for all” report published in 2008 [38]. As services were suddenly reconfigured, staff were redeployed outside of their speciality and visitation from relatives and carers was limited, there were major impacts across the general population. However, preliminary evidence from Public Health England reports disproportionately higher numbers of people with learning disabilities who have died from Covid-19 [39], which may in part, be a result of existing inequalities to risk factors including respiratory disease and diabetes [40, 41] and lifestyle factors including physical inactivity, obesity, poor housing conditions, and smoking [42–45]. Nevertheless, systemic barriers in care which are highlighted here are likely to play a significant role. For instance, the increased gaps in care and a limited ability for carers, families and advocates to step in and provide protective factors to safety found here could be putting the most vulnerable at the most risk. The report proposes that death of those with learning disabilities is currently estimated at 4.1 times higher than the general population when adjusting for factors including sex and age, however, not all people with learning disabilities are registered within the databases used, therefore, it is expected to be higher in reality.

### Implications for research and theory

One way to consider these issues is through the lens of the ‘gaps, traps, bridges and props’ framework, previously developed to explain systemic safety issues in patient safety [46]. Supported by earlier research exploring the relationship between having learning disabilities and safety outcomes [10], our findings suggest that there are indeed both ‘gaps’ and ‘traps’ created by health and care infrastructure, which are likely to result in people with learning disabilities experiencing poorer safety outcomes in comparison to the general population. For instance, the design and delivery of healthcare interactions can cause unnecessary distress and iatrogenic harm, and reduce opportunities to establish effective communication can burden families and carers, and risk patients navigating the system without the appropriate information to provide informed consent. Examples include widened gaps during transitions of care, inadequate staffing levels with limited learning disabilities expertise and an inability for local level safety initiatives to speak to one another within and between settings, having direct and indirect implications for safety. Nonetheless, the findings also highlight potential bridges and props, which both formally and informally support the system where there are risks or weaknesses. This includes providing people with learning disabilities with the appropriate time and space during interactions such as designated quiet rooms, and ensuring that all information is presented in easy read and understanding is confirmed. The most prevalent mechanism for supporting patients with learning disabilities however, often came through those outside of the official system, by those who regularly ‘prop’ up services from their role in advocacy services, or as families and carers. The ways in which these props were provided was wide ranging, including pragmatic and emotional support, assistance in navigating the system and exercising rights. It is evident that without these ‘props’, the safety outcomes for people with learning disabilities may be significantly poorer.

### Implications for policy and practice

These findings have wide ranging implications for policy makers, those providing care to people with learning disabilities across settings, carers, families and people with learning disabilities themselves. Our qualitative exploration evidences specific risk factors and potential protective buffers to patient safety issues for this population, some of which are directly attributed to having learning disabilities, whereas many are a result of the interactivity between those and safety gaps causing heightened inequities. Therefore, we propose that effective interventions should be developed to promote the optimum environment in which to ensure high quality and safe care, and alleviate longstanding risks to this population, which should be tackled at both a policy and a local level across care settings.

### Limitations

Care Opinion enables the easy identification of feedback concerning services specifically designed for those with learning disabilities, and/or in which learning disabilities are explicitly discussed. However, feedback regarding non-specialised services where reference to learning disabilities were omitted, may have been missed. The severity of learning disabilities may have also highlighted patient safety risks for particular groups of people, however this could not be explored, as participants were not required to report this information. Additionally, the study had to be adapted due to Covid-19, including stopping recruitment to the focus groups. However, feedback from Care Opinion was used to supplement data from the two focus groups, increasing richness and variety. Finally, carers were invited to participate in the focus group sessions, however, none consented to take part, but their views were captured widely within the Care Opinion feedback.

## Conclusions

Our study found that people with learning disabilities experience a range of safety problems when receiving health and social care. These are problems not directly caused by their learning disabilities, but rather exacerbated by them as people navigate around and exist within a health and care social system that is not designed to meet their needs. This leads to a situation where people with learning disabilities, their families and carers are forced to operate as the malleable buffer between rigid service design and delivery and negative safety and health outcomes; a situation that creates problems for those ‘stepping in’, as well as increasing inequities for those without social capital. For these safety inequities to be addressed, it is evident that policy level change is urgently needed to tackle some of the systemic ‘safety gaps’. However, future research definitely has a role in designing and testing interventions that can reduce the impact of existing gaps and traps that exacerbate the likelihood of safety inequities.

## Supplementary Information

**Additional file 1.**

**Additional file 2.**

**Additional file 3.**

## Data Availability

The supporting data set is included within the articles additional files (see Additional Files 1, 2 and 3).

## Abbreviations

CIPOLD: Confidential Inquiry into Premature Deaths of People with Learning Disabilities
COREQ: Consolidated criteria for reporting qualitative research
NICE: National Institute for Health and Care Excellence
LeDeR: Learning Disability Mortality Review
NIHR: National Institute for Health Research
NIHR YH PSTRC: National Institute for Health Research Yorkshire and Humber Patient
PIPS: Patient Involvement in Patient Safety
NPSA: National Patient Safety Agency
PSTRC: Patient Safety Translational Research Centre
SoMREC: School of Medicine Research Ethics Committee
YQSR: Yorkshire Quality and Safety Research Group
