# Supplementary material for: Systemic safety inequities for people with learning disabilities: a qualitative integrative analysis of the experiences of English health and social care for people with learning disabilities, their families and carers
Source: Int J Equity Health. 2022 Jan 28;21:13. doi: 10.1186/s12939-021-01612-1 (PMC8795982; doi:10.1186/s12939-021-01612-1)
Supplement: Supplementary file 1 — Additional file 1. [file 12939_2021_1612_MOESM1_ESM.docx]

| Care Opinion feedback |
| --- |
| I want to go back to work I love to go gym I happy with food I going healthy eating I want ipad phone I want more money I want paid my gym off I want my flat back I want get fit and lose fat |
| I feel happy to be here Nothing wrong staff nothing wrong |
| A. T. S. is poorest one I have been in Low in staff = not good in community: (while here) Safeguarding not good; not very organized: (to get more staff) |
| Sometimes it's good when you go out to shop's/lunch/cafe. Don't like not going out. They look after me well here. I like to make phone call's/more if possible. |
| Happy with my care. Happy with staff and how they respect me, Everythings going well for me |
| I was taken into a room by a counsellor and was shocked to find another counsellor waiting for us. I was expecting only one on one, and this immediately threw me.The assessment was done by both of them together, which was really difficult, because I couldn't focus on both of them. Felt they were ganging up on me. One of them asked some questions which can be summed up by: Do you feel special for having visions and seeing things, because everyone's a little kooky, aren't they? You realise that all of this is inside your own head, and just your perception of the world? You present too well, and you are very articulate, therefore you must be ok really. I felt unheard and unseen and very judged. I felt that one counsellor in particular was not on my side. I felt to them I was just a statistic, and unless I had an actual gun to my head, I was beneath their notice. There was a complete lack of empathy and understanding. At the end they gave me a list of 'fun things to do' (!) and a referral to CBT counselling. I had already explained that I had had CBT several times and it did nothing for me. At the end I just closed up and said yes to everything because I felt very uncomfortable and that the session had been a complete waste of time. I felt embarrassed that I had opened up to them. I left feeling worse than before I had gone in, and wished I hadn't gone. |
| Lost my wife a year ago this time. I felt the standard of care has been atrocious and for three months someone visited for half a hour a week. No empathy or connection. Pill checkers really. Are you taking your tabs sorts and they are gone. Then the house I'm living in had a major flooding issue with the bathroom and kitchen requiring a major refit. The mental health sorted me a room in this hotel, they've never visited me here. Feel I've been imprisoned here. With a shop doorway as a alternative. All my sentimental things from the years with my wife at the house. So it is a very depressing situation, and I'm supposed to be thankful for it because the alternative is a shop doorway. |
| Area of building great, Facilities great, Staff great, Therapist great. |
| Poor service, misdiagnosis and inadequate supports - 6.5 years of my life wasted. After being misdiagnosed with bipolar in 2015 I requested a further assessment as I suspected I had adult ADHD. After waiting over a year and a half, during this time I lost my job due to the side effects of the medication I was on for a condition I didn't have. Once reassessed and ADHD confirmed I was seen by the ADHD services once regarding medications and discharged back to my GP. After taking a reaction to the medication I requested to be re-referred to trial a new medication (as my GP couldn't prescribe). After a further waiting period of one year I was finally seen. During this time I was seen by a private psychiatrist who also diagnosed dysthemia (severe depression which was treatment resistant). I spent all my savings just to access treatment. The private psychiatrists reports were never used and not accepted by the GP practice - further delaying my treatment by 1 year. During this time I had to move house and my marriage broke down due to the total decline in my mental health and depression. After 18 months I am still awaiting treatment for my ADHD. During my last meeting with a psychiatrist they refused to prescribe due to the chaos in my situation. They actually wanted me to get my life in order to prove that it was ADHD! ! ! The psychiatrist's knowledge of ADHD was shocking considering They were working in this particular area, I challenged this strongly and insisted it was discussed by the team. The decision was overturned and I am now awaiting my next appointment to start treatment. My son has ADHD and the support and understanding within Ryegate is incredible and I have a lot of input into his care and treatment. Through SPA it feels as if I'm being treated as a mental health service user not a Neuro-developmental service user. Absolutely appalling experience which has had such a detrimental impact on my life - I've lost 6. 5 years of my life due to the absolute incompetence of Sheffield Mental health services, GP and Neurodevelopmental services. This has affected my son which is completely unacceptable. I've asked if they'd find this level of service acceptable if it was a member of their family, uncomfortable silence or deflection by all.. However the reception staff have been amazing and really supportive, professional and efficient, it's just a pity that it's not seen in the actual medical team. |
| Called to confirm an appointment later that day because hadn't received a letter about it or a response to the phone message I'd left a few days prior, the receptionist kept interrupting me and wouldn't let me finish asking my question (which is particularly bad for a service dealing with autistic patients as when interrupted I lose track of what I'm saying), then hung up on me and set my number to go straight to answering machine. I later got a call back from the staff member my appointment was with confirming that I did indeed have an appointment that day, but was less able to communicate during it due to the stress of the earlier phone interaction. |
| I was in need of talk to a doctor about same blood in the urine and same little cramp in my uterus....I was refer to talk urgently to a doctor by a Walking in Edgware with a letter and a proof of slip that I had moderate blood in urine....I talk to a receptionist of it and show the letter...But they refused to give me an urgent appointment....So I was force to go to Barnet hospital that refer me to urologist care... |
| Dear Hilary, Anna, all at St Luke’s Single Point of Access (SPA), all at St Luke’s day services, Alison, Dr Holland, Dr Phoebe and everyone else who looked after my mother during her recent illness. Please do pass this on to anyone at St Luke’s who I have missed. I will also send some thank you cards, again, hoping they will be shown to everyone who looked after my mother. I would like to thank everyone who supported my father, Mark, and looked after my mother, Helen, during her recent illness. You probably know that she died in January at home. I am sorry it has taken so long to thank you, but practicalities have taken up a lot of time. She received the most amazing care from the NHS in her last few months, but particularly in the last week. There was always a nurse available to answer the SPA phone line, ready to give excellent advice, or to contact an appropriate service to help my mother. She had support from St Luke’s day services, the Community Palliative Care team, Dr Bleehen (GP at the Pinn Medical Centre), a visit from an out of hours GP, the district nurses, and the ambulance service. There was also a very kind and helpful private lymphoedema nurse, who visited her at home, and the Caremark agency (not NHS), who provided some really caring carers. This was the NHS at its best! Even after my mother had died, you were all so caring and helpful, taking time to talk to all of us, even when we felt your time might be better spent with those who were ill. Once again, thank you so much for all your help and support over the last few months, and just to let you know that we are all coping well. I am sure this is largely due to the fact that her last few weeks and days were peaceful and comfortable due to the amazing service we received from you all. |
| I am writing to convey my appreciation for the exceptional nursing care provided by Calvin. Throughout a five year period of an extremely tempestuous time of my mother's rapidly declining health, my mother has experienced Calvin's practice of nursing within a number of different demographic locations. These include: sheltered accommodation flat; psychiatric hospitalisation, Residential home and Nursing Home. It has to be highlighted that from the outset when we first met Calvin, her nursing practise was vividly characterised with enthusiasm, kindness, respect and a genuine pragmatic concern for my mother's psychological, physical, social, environmental and medical well-being. Calvin's effectiveness as a psychiatric nurse becomes most evident by the way she attentively listens and offers encouragement to my mother. Calvin has a repertoire of both "formal" and "informal" approaches to communicate effectively with my mother. I highlight these particular factors because often my mother can be resistive to care, shouting at staff and refusing essential medication. During these extremely challenging times; whenever the nursing staff at Rosedene are unable to effectively cope with my mother's challenging behaviour, Calvin becomes an indispensable asset to both the medical and nursing team. It has to be emphatically noted, that Calvin's effectiveness as a nurse practitioner has been achieved through diligent building and nurturing a strong rapport and trusting relationship with my mother. So, whenever my mother exhibits challenging behaviour or becomes extremely agitated with carers and nursing staff attempting to deliver care, Calvin's visits and understanding approach demonstrates her medical awareness and psychological insights into my mother's complex health needs. She has the ability to appropriate and apply her valuable interpersonal skills, which incorporates both patience and perseverance in her vital role to ensure that my mother remains consistently concordant with prescribed mental and physical health medications. Calvin also ensures that necessary medical reviews are carried out and inexperienced nursing staff at Rosedene are consistently following the correct nursing procedures and medical protocols. I do strongly believe that without Calvin's specialised support Timely interventions and monitoring (when necessary) my mother's health needs, my mother would be at risk of not having adequate specialised psychiatric care. Particularly during a time when senior management and nursing staff at Rosedene have been rapidly and constantly changing. I have been extremely impressed by Calvin's holistic approach to nursing and her detailed and coherent delivery of care. There is no doubt that Calvin has a wide range of personal and professional skills, which have equipped her well while navigating my mother's challenging and complex health needs. It also has to be highlighted that Calvin always remembers the importance of the anniversary date of my mother's husband's death. This is another graphic illustration of Calvin's genuine compassion and conscientious duty of care. Her recognising my mother's individuality and uniqueness means that Calvin has a comprehensive understanding of my mother's essential health care needs. Equally significant to note: Calvin fully understands the importance of working in 'partnership' with both 'patient', other 'caring practitioners' and relevant family representative'; to ensure that her care strategy and method is both appropriate and effective. This important 'partnership dynamic' has been graphically illustrated throughout her nursing practice. Although her principal focus is 'always' on my mother, Calvin regularly enquires about the well being of myself and immediate family members. Calvin has binocular nursing care vision! She always has one eye on her patient and the other on the overall picture! Therefore Calvin gains valuable insights to mother's care needs and well-being. Indeed, Calvin is to be commended for her effective and efficient approach to psychiatric nursing. From my observations and experience she has been consistently reliable, supportive, co-operative, sensitively caring, well-liked by my mother, punctilious in all matters relating to safeguarding. She is loyal and committed to her vocation of nursing and conscientious in her duty of care. Calvin -thank you for your care and for all that you do |
| I have stage five kidney disease and self-care at home where I have hemodialysis 4 times a week for 3 hours each time. I see my consultant every three months and between those periods I am under the care of the home therapies team based at Loughborough Hospital, Leicestershire. Following my last three consultant appointments my consultant as written to my GP requesting that they amend several items on my repeat prescription, however, they are failing to do so. I have written to my practice manager and had no reply and complained to The GMC via email and had no reply. I really do not know what coarse of action to do next. |
| In the past, I have required treatment for lower back problems, foot issues and now currently physio following a broken arm. The physio team are patient, polite and totally professional at all times. With early treatment and suggested continued home exercises I've been able to return to my daily routine pain free. The Back Class was very informative and helpful, I would highly recommend this course if offered as being helpful for back problems. With a broken humerus I am again under their care and although progress, on this ocassion, is slow I have already seen an improvement and my confidence given a necessary boost. A very big thank you to my doctor at the Oakham Practice for the early referrals and the Physio Department based in the Rutland Memorial Hospital for their treatment. |
| Tipped up with what I thought was a sprained wrist - was seen within an hour and sent immediately for X ray - well, walked 10paces round the corner - immediate X-ray , immediate results showed a difficult break which required orthopaedics to look at it. I elected to go to Peterborough where I was seen, manipulated, plastered and sent home. It did eventually require a metal plate which was inserted the following week. Thank you RMH, God bless the NHS and happy 70th birthday... |
| I arrived at the Oakham MIU at 8.30, as the website stated it opened at this time. The very helpful staff explained that although the unit is open, the first appointment is not until 9am. They have tried to change the information on the website but their requests have not been acted on. Annoying as I only came to Oakham MIU (rather than Stamford) due to the earlier opening time. |
| Had cause to visit this unit earlier today and cannot praise the staff enough. Efficient and speedy service. Thank goodness for the NHS! All dealt with within an hour as a walk in patient. Thank you |
| We visited a past work colleague who was recuperating after surgery in Leicester. Finding the hospital is not difficult, it is reasonably well signposted within Oakham, but we were surprised that it was not identified on our generally well-up-to-date on-board GPS. So, if you are unfamiliar with the area we suggest that you make sure that you enter the Post Code in your GPS and keep a good lookout for the entrance to the car park (the hospital is screened by trees and is not a tower block!). We had no information re where our colleague was located within the hospital. A name was sufficient at Reception to readily elicit some directions, and this was subsequently repeated with another member of staff as we homed in on our destination. Conclusion – the staff know the patients. Everything we saw during our visit was fine. The car parking arrangements are an added bonus; the polar opposite of many hospitals. |
| After telephoning 111 service at 11.30 am on a Saturday, I spoke with the doctor on duty. He was extremely knowledgeable, empathetic and helpful. I had to travel approx 14 miles, but this is far less than the 20 mile journey to my local large A&E hospital. I was duly offered a appointment for 12.30pm. I arrived 5 minutes early, and the Doctor was waiting for me in reception ! Having been called in immediately, a thorough consultation and examination was then conducted. I had been in extreme pain since 2am, and he was exceedingly gentle and thorough......prescribing a variety of medications, which my partner was able to collect from the pharmacy situated next door to the hospital immediately. Arrived back home around 1.30pm.....WOW ! My pain has now been alleviated. I cannot speak highly enough of this service....... thank you, thank you, thank you. |
| My daughter , a boarder @ Oakham School was sent by the medical centre for the school to be seen by the hospital. The problem is a rash covering the whole body. The girl is to start ifnal IB examinations in two days. The Hospitel turned her down for adminsitrative reasons twice today , e.g. like not being registered which is ridiculous for a boarder girl, where the school ensures that they all are registered. Finally being attended by a teacher an appointment materialized. |
| Seen shortly before appointment time. The dermatologist was very approachable, professional and reassuring, very clear on further treatment and timings. Worked as a team with other colleague. All good , thanks :) |
| To all on Rutland Ward. Words alone cannot express my thanks for the wonderful help and support you gave to my Father during his time at the Oakham Memorial Hospital. There was always a smile on everyone's face, a joke to be shared and a laugh to be had. Thank you so much |
| Everyone is awesome and fantastic, I wouldn't be alive right now without with help and support they have gave me. |
| Ashgreen are a life saver they have saved my life lots of time's they are great with me and others. Ashgreen is my safe place. |
| 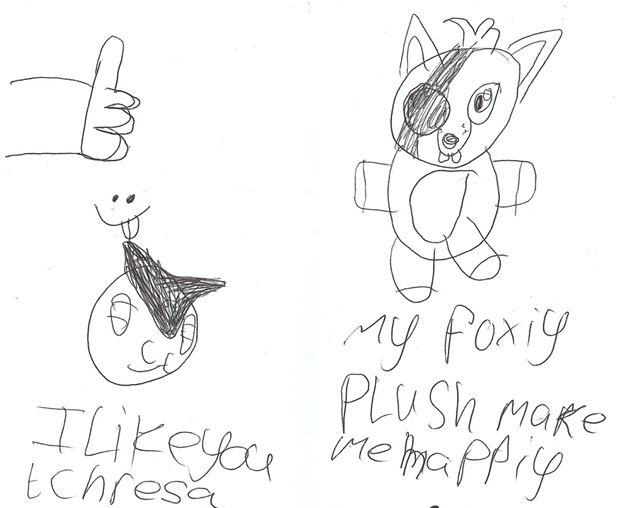   \|  \| \| --- \| |
| I'm happy with all the support we have had, I have been fully explained things and listened to and happy that my son feels comfortable and able to speak about his problems |
| We met Diane a couple of years ago in a very difficult time in our family. Our autistic son was experiencing a stressful time with changes in our family life and the arrival of his sister. Our Son was very stressed and violent. Diane was able to spot very quickly some of the key triggers that were causing these violent outbursts. She was able to advise and help to put into place a number of strategies to calm him down reducing his outbursts and anxiety. Diane also put us in touch and organised appointments with other professionals (e.g. psychiatrist) who she thought would be able to help. This proved to be very successful and we wouldn't have been aware of this service without Diane's input. Diane liaised with staff at school to advise on how best to support our son in his difficult time. She attended the meetings we had with school and she was very good at pointing out what was causing problems at school and how the school could deal with it.Diane has continued to support our family as his behaviour improved and helped us with further advice and strategies to help him to deal with day to day life.We are eternally grateful to Diane for all the support she has provided to our family during the last two years. |
| It was so nice for our daughter to be put at ease. Thank you. |
| Made my daughter feel relaxed and looked forward to her treatment. |
| The treatment at Park House Dental Clinic (although a bit unpleasant) was made fun by Monica and the dental nurse who encouraged and praised consistently. Thank you for making the treatments less scary! |
| Fantastic service at Park House Dental Clinic. It really helped restore my daughters confidence after having lots of toothache. |
| The dentist is very gentle but there is no air conditioning in any of the rooms and it is warm. I feel clammy especially in spring/summer. |
| Everyone at Park House Dental Clinic has been kind, generous with their time and super-professional. My son came here with a number of problems - and he had been made anxious and afraid by other dentists. You made him feel safe and involved in his dental treatment. Thank you for that! |
| Monica was very patient, caring and smiley throughout the process. All the procedures were explained to my son in a child friendly way which enabled the experience to be a happy one. Thank you also to the other staff here at Park House as they have also been very pleasant and approachable, Many thanks |
| The dentist and nurse at Hyson Green Dental Clinic spent so much time patiently gaining my sons trust and have just managed to scrape off a massive build up of tartar. If I wasn't in the room watching I still don't think that I would believe it. I honestly didn't think anyone would be able to get near my sons teeth, he has so many difficulties. Thank you so much. |
| My son's teeth were in poor condition however with several fillings and two crowns to protect two of the deeper fillings I feel much happier my sons baby teeth will last until the adult teeth appear. |
| I came today with a very nervous son. The dentist we saw at Park House was fantastic, informative and very empathetic with regards his nerves. They explained everything slowly and addressed him rather than me and this did really help. |
| It's never a good experience when your 4 year old son has to have a tooth out but all the staff at Park House Dental Clinic made it as painless as possible. Monica's treatment of my son was lovely. Thank you very much. |
| Brilliant treatment at Park House Dental Clinic. Made my daughter very relaxed and at ease and explained everything to her before and after treatment. |
| Very positive attitude when visiting the dentist. My daughter always arrived and left with a smile. |
| Treatment was really good at Park House Dental Clinic. Nervous but put at ease and very comfortable with the procedure. Thank you. |
| Before my son came to Park House, he wouldn't even go near let alone go in the dentist chair. However the staff, especially Monica have been amazing and now he looks forward to coming along and let Monica look into his mouth and show how well his teeth are coming along. Due to some problems with his back teeth, he needed some fillings and wouldn't allow this to be done unless he had happy air. However on his last two appointments my son was brave enough and confident enough for any work needed to be done without using/needing any happy air. His confidence has increased significantly and I personally would like to thank all the team -especially Monica for giving my son this confidence. You have all been amazing including Joy for arranging the appointments, letting us know of any cancellations and calling the day before to remind you of the appointment date and time. Thank you so much! |
| My daughter had a good experience of a tooth extraction - which feels like an odd thing to say. The staff were very patient with her and made her feel at ease. She was very worried beforehand. |
| Would like to say thank you to all staff at Parkhouse, they have been great with my daughter. She is happy to come to the dentist. They have spent a lot of time with her to make her feel safe. I would say they have gone beyond what they should do. Thank you to all. Thank you for helping me look after my teeth, I really appreciate it :) |
| The staff are always friendly and helpful at Park House Dental Clinic |
| My daughter attended Park House Dental Practice on three occasions. The service was excellent on all occasions. Monica had a lovely manner in terms of friendliness, putting my daughter at ease, keeping her informed of what was going to happen and why and communicating in a manner suitable to her age. This was a much better experience compared to her normal dentist who were unable to fully extract her tooth and were lacking in their ability to communicate with my daughter in a manner which put her at ease and trusting enough to have the treatment. |
| I arrived with great trepidation as I have an exceptionally nervous child who categorically stated "he was never going to the dentist again". The consultation process and treatment he received was fantastic, caring staff who talked him through the process at every stage. Monica was so caring and patient. My son has realised the importance of having treatment quickly when needed. His treatment was personal, they always greeted him by name and asked him questions rather than referring to me. I cannot rate the staff and treatment highly enough, utterly exceptional. Many thanks |
| Fantastic people skills |
| Thank you for all your help |
| Excellent experience and treatment at Park House Dental Clinic for my 6 Year Old. Monica was very patient with him and went into detail with every step of his treatment process which helped him feel comfortable with everything. |
| My daughter has come to Park House for treatment over a period of months. Staff have been very supportive and caring towards us and always very clear about procedures. It has been made a lot easier for my daughter being able to receive her treatment here. |
| We've had the help and treatment from Monica at Park House for my sons teeth for a number of years. She has always done a lovely job of keeping him at ease during the treatment and establishing the best course of action for his treatment. Thank you to Monica and the team. |
| My daughter was referred for the services at Park House Dental by her dentist as she was a nervous 7 year old patient who was not able to open her mouth for very long. The dentists at Park House made friends with my daughter and put her at ease and in my daughters words "I really liked it, it was really good". The service is fantastic and professional and just what someone like my daughter needed. We are very grateful to the staff. Thank you. &nbsp; |
| My son will let you do anything that he wouldn't allow before. Fantastic friendly receptionist. All really helpful and would recommend 110%! |
| Park House Dental Clinic. Staff are always super patient and make you feel very good. |
| Park House Dental Clinic. Both the dentists were really nice, they were called Lauren and Monica. They explained everything that happened and they were very careful. |
| My daughter was extremely nervous about her treatment but Monica and her assistants at Park House Dental Clinic were great in reassuring her and making her comfortable. She now has no fears when attending. Information and advice has always been clear and well explained. Thanks for everything! |
| They made me feel happy and safe at Park House Dental Clinic. |
| My daughter was terrified of the dentist before she came to Park House. Now she cannot wait to come here. All the staff are brilliant, so much patience and kind. Thank you all so much. |
| Clifton Dental Clinic. Kathryn is a fabulous 1st contact, telephone and face to face. Friendly with patients and "open", also putting people at ease with her pride and respect for who she works with. The patient care for my grandson with A. S. D, ADHD was outstanding. He was nervous and would normally display vocal and bodily tics and jerks. Hannah the dentist and Charlotte her assistant spoke in a clear, honest and reassuring manner. The dental procedure was carried out so smoothly with obvious skill from both practitioners. Thank you. |
| Brill service for my Daughter! |
| The receptionist was very nice. The dentist R. Gembali is lovely and kind and always encourages me in my dental health. The dental nurse Sharo was just lovely, made me feel at ease and made sure my financial details on my debit card remained private. Thank you for always making me feel welcome and your kindness. |
| My daughters anxiety was taken into account at all times at Hucknall Dental Clinic. They took their time with her even when she persisted to have the treatment. |
| Attended with my 8 year old son for a tooth extraction. The chair was broken so we couldn't use laughing gas as planned BUT Hannah K and Charlotte T were AMAZING. Provided my son with lots of advice for treatment options and when he chose to proceed without gas, gave lots of reassurance so he was super comfortable and relaxed - an excellent experience (in the circumstances!! :)  Thanks you! |
| Ginny, the dental hygienist was very professional when she treated me. More importantly to me, she was very encouraging to me about the way I was looking after my teeth and gums. She also provided me with thorough and useful information about how I could remove plaque and what products I would be able to use. Her assistant was also a lovely, professional woman. They are both very kind people. |
| ou were very kind at Park House Dental Clinic. Thank you. |
| All the staff at Park House are amazing, They are so friendly and caring and made everything really easy and comfortable for my daughter. Keep up the great work team, you are all amazing. |
| All the ladies are lovely at Clifton. Put you at ease. Ginny is a very understanding fabulous dental therapist, explaining everything to my daughter giving her confidence.. This is an excellent service. The receptionist Kathryn is very welcoming, friendly and efficient. |
| My daughter had a tooth removed but was scared of needles so we were referred to the dental suite at Clifton Cornerstone. The team made her feel at ease and looked after her throughout the whole process. |
| Lovely warm and friendly service |
| Really amazing service. Thank you! |
| Thank you for your thoughtful caring attitude to Mum. For respecting her views, for giving her time. It has been wonderful to bring her. She has commented "I like those two young ladies at at the new Dentist. They have a lovely attitude". |
| We attend the special needs dentist at the Meadows Health Centre. My husband has a nervous disposition, and the staff are very kind and patient, and explain to my husband. They give him the confidence, and go at a speed that he can cope with. Thank you to them, as I felt we would never get his teeth seen to, and he can cope. He stays in his wheelchair, and they just take the wheelchair up that he can cope with, and never rush him. Thank you |
| My daughter came for a tooth to be taken out. She was very scared. The staff from reception, dental assistant to dentist were so friendly and brilliant with my daughter. Lovely people, thank you. |
| Mary Potter Centre. For the 1st time ever our daughter who is 13yrs old, actually let the dentist check her teeth. All staff were excellent. very calming, patient, and reassuring. Thank you. |
| The dentist that helped to treat my teeth (Monica) made me feel comfortable and happy! All my teeth are now healthy and strong and I was told the best ways to care for my teeth. Thank you. |
| Compassionate and thoughtful. Put my sons at ease and dealt with his concerns. Thank you! |
| Talking through and counting as the treatment is carried out really helped. Being able to ask lots of questions was good. Monica is amazing |
| Couldn't ask for a better therapist, so supportive, helpful and gave me so much confidence when I was at a real low. |
| The staff were outstanding. Monica is a fantastic dentist with young children and made my child feel comfortable and happy. Brilliant service, please keep it open and going for all. |
| All staff were very friendly and helped my child feel at ease. His treatment was always done to a high standard and as pain free as possible. A huge thank you to all the staff especially Monica! |
| My daughter is very anxious but all the staff at Park House Dental Clinic reassured her and were very understanding and patient. |
| Having had a bad experience leading to a two year wait for it to be taken out, this has by far been the best experience for our Daughter. She even said thank you. We can't thank the staff enough. |
| My daughter suffers with anxiety and was unable to complete her dental treatment with our usual dentist so she was referred to the Salaried Dental Service. She was extremely anxious on her first visit but all the staff were fantastic with her. She has attended 5 times in total and was actually looking forward to her last appointment. A huge thank you to all in the department for making what would be a very stressful experience for both me and my daughter alot easier. |
| Monica is great with children, always approachable and professional. Since my daughter has been attending the dentist here she has been relaxed, made to feel comfortable. Everything has always been explained to her so she understands what is going on and what she needs to do. The treatment and care here at Park House is the best we have had and my daughter loves coming here despite what treatment she has. Cannot fault anything or anyone in the practice. Thank you all so much. |
| Treated with great dignity and patience. Good humour and respect by all staff. |
| Thanks to the staff, not only are my daughters teeth sorted she is no longer scared of the dentist. Thank you so much. |
| My 7 year old daughter was referred by our dentist and the treatment received was excellent |
| All the staff at Hucknall Dental Clinic are brilliant with children and made my daughter feel at ease. Brilliant. |
| Everything at meadows Dental Clinic was very good. Everyone treated me well and I was very pleased with this service and the receptionist was very nice. |
| The initial assessment at Park House was very detailed in terms of the process, explaining what would happen, then my daughter had another assessment at Mary Potter, Hyson Green and he explained about "happy air". On the day of the extraction Monica explained what she was doing and talked my daughter through the procedure. Gave us information on the follow-up appointment and eating and drinking. |
| Doctor was friendly and professional, we don't have to wait on our appointments. Thanks |
| The staff were lovely, and reassuring. my son is disabled and he was so relaxed with Charlotte. Thumbs up thanks NHS |
| The best service I have received from a dentist |
| Very polite and helpful dentist at Park House Dental Clinic. Thank you |
| Always had a fear of dentists but have been made completely at ease by staff here. Thanks for all your help. |
| Thanks for your helping |
| Friendly staff and very patient with children at Park House, Dental Clinic |
| Amazing service from all staff at Mansfield community dental. Without being able to use this service I wouldn't get the care I need for my teeth as I am unable to use a general dental practice |
| By far the best dentist I have ever experienced and I've moved around over the years and sampled quite a lot. This dentist can be trusted to be kind, fair and do the job properly. My son has disabilities and taking him to the dentist was a complete nightmare but since coming to the Clifton Cornerstone Dental Clinic he has really enjoyed it, they take plenty of time, interact with him really well and save so much unwanted stress. I suggest the parents of disabled children should be able to be seen here too, it would certainly encourage me to return to the dentist. |
| Excellent as always |
| Absolutely brilliant service here. Made my autistic son feel very at ease. From the first time he came he was very distressed, now after a few visits he will sit in the chair with ease and is quite happy. Many thanks |
| I bring my son with ASD to see the Dentist and therapist, every visit we feel very well informed and all care for billy is very good! My son's Autism gives him a very tricky character but all staff are great with him and even more importantly very patient! Thank you for making such a tricky experience quite simple. |
| Great all round Staff brilliant |
| Very caring staff. Both our children have special needs and all the staff involved are very understanding and patient. I would recommend this place to everyone and never want to go anywhere else. Thank you for being so good at what you do for us. |
| Thanks for good service |
| Excellent - Wish there was more dentists like this. Well done. |
| Received good care + makes you feel comfortable. |
| I had a brain haemorrhage and a speech and language disability (aphasia), 26 years ago, aged nearly 29. I’m very fortunate that I go to the Hyson Green Dental Department and they are so good in terms of my speech and language disability (aphasia) and making it more accessible for me. Thank you! |
| The care I received from Vanessa and Mandy in the Special Needs Dental Service at the Mansfield Community Hospital was outstanding. As a patient who suffers extreme anxiety and fear of all medical services, I felt so supported and looked after. From the moment I walked in to the moment I left, I was extremely well cared for. These medical professionals deserve the recognition for their outstanding work in the dental services. It takes a lot for me to feel safe and trust in something I fear so much but I have every faith in these ladies and the care they provide. Thank you both: ) |
| A caring professional dentist who really goes the extra mile for his patients, he made my daughter feel unhurried, cared for and valued. My daughter has autism and has had a general fear of dentists in her 23 years. Ram made her experience a positive one and she completed treatment. Excellent nurse who reassured her throughout. The receptionist phoning us to remind us is excellent. telephone manner and excellent service. Thank you to all! |
| Excellent service from the Mary Potter Health Centre Dental Department on the day. However, when we received the reminder telephone call we were told that we had a dental appointment but we were not given the time. As we were't in a message was left on the answerphone. The message did say that if we didn't know the time of the appointment to ring (and gave number). However since this is an appointment for people with special needs it seems like this would be an unnecessary complication. My own NHS dentist reminder always gives the date and the time. |
| My son is a different person following help and support from the Criminal Justice Liaison and Diversion Team and Mansfield Community Speech and Language therapist and the mental health teams. Because of the Criminal Justice Teams intervention my son was given access to the Mansfield mental health team who were able to get to the root of his behavioural problems that led to criminality and drug abuse. They were able to refer him for speech and language therapy also which has also helped enormously and through coordination have got my son into an assisted flat where they can all keep an eye on him and continue to give him access to the therapies he needs, and has needed for a long time. My son is calmer and less frustrated and angry, his behaviour has changed so that he speaks more to the rest of the family, bringing us all closer. Without this we would have been driven further apart, I thought it would end in tragedy. we now have a better understanding of why he has behaved the way he has. Catherine in the Criminal Justice team and Gemma the speech therapist have been brilliant. |
| My Named Nurse is sometimes kind to me but sometimes if I am not being good they take my radio and teddy bear off me at Night, why is that I don’t like it when they take things off me. I love my teddy bear and I just dont understand when they take things off me. I think I have a care plan but not sure. I like to visit the shop here the Staff are nice there. I also like all the Staff at the Southwell day centre, I wish I could go more but if I am bad I dont seem to go, why is that? |
| I am a patient within the Learning Disability Service. I like going to Graphics, I can be myself and it helps me relax. I get to learn new jobs such as gluing, laminating, cutting and making booklets. I make things for myself, for presents and for the hospital. It is important that patients have the chance to go to work areas as it is part of their therapy and treatment within a high secure hospital, I think people’s placements at medium secure sometimes fail if they haven’t had the chance to go to workshops as they aren’t used to mixing with other patients and learn new skills.Having vocational areas is helpful as it helps with mental health and behavioural issues and psychological issues which can be a daily battle for some people. I have developed positive relationships with the staff in Graphics and get to meet other people like visitors who come to the area and have a good relationship with office staff who come into the workshop. I like the relaxed atmosphere, staff respect me, I can speak my mind and are accepting of patients choices and don’t discriminate. Overall the message I want to say is that going to workshops means I can be myself, it makes me feel normal and helps give me mental stability and achieve my goals.” |
| I am a Patient from the Learning disability service. I have enjoyed doing the Interviews here at Rampton. I think this experience helps give me confidence and help me to learn how to be polite and respectful. I get to be involved with Staff who could be caring or working with Patients here at Rampton. |
| Coming into Rampton from a journey that’s been disruptive and arriving at Rampton opened my eyes to what the High Secure Service could offer me. Rampton Hospital Learning Disability supported me in my transfer from MSU and now I realise that I can control my behaviour, have treatment, medication and move back to medium secure. High secure services saved my life and supported me through the bad times, showing me that recovery could be possible. In the past staff couldn’t get near me, now I am open to being cared for. Some days I need reassurance, other days I can cope. |
| 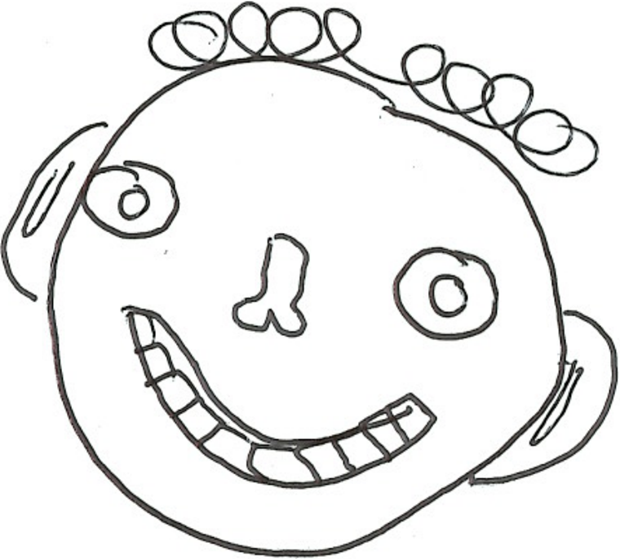   \|  \| \| --- \| |
| IDD Occupational Therapy - I feel I had to wait a long time for my first appointment - I understood my OT care plan - OT has helped me I enjoyed attending the gardening group, we played some games, made wraps and I liked meeting new people - The OT service could be improved by: -being seen quicker -the group running again for others |
| Thank you for all your help and support Rob, |
| My care Services was fantastic. I was listened to, not judged, talked about safety. Fantastic staff. |
| Speech and language therapy (signing and speech session) really helped me a lot and its really make me happy. I really enjoy it and people (my friends, family, girlfriend) will understand what I'm saying and I can also teach them how to sign in Makaton Thank you to Charlotte and Amy for being the best |
| In SLT I could talk about whats on my mind in a safe environment, while also having a laugh. It helped me talk about difficult topics, and understand my issue to confront it |
| 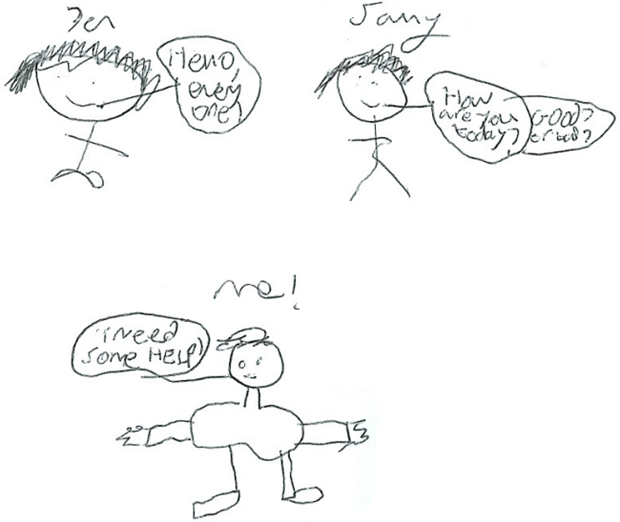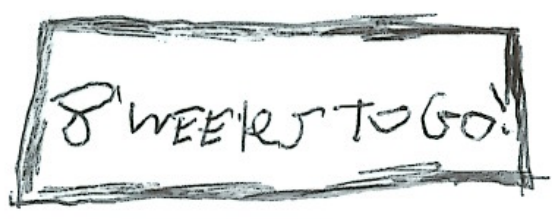   \| I'm really enjoyed this experience, but, some people were really struggled, so, I'm helped them! Jen and Sally were talking to students at Portland College and they were brilliant talking to us everyday at "speeches and languages" sessions. And, what I really like to say that next year hopefully might come back and joining us again with some more games or just a sessions every day in 8 weeks or so again! \| \| --- \| |
| 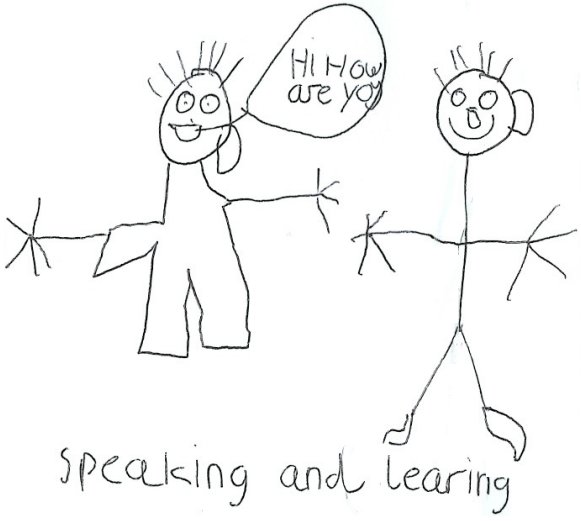   \|  \| \| --- \| |
| 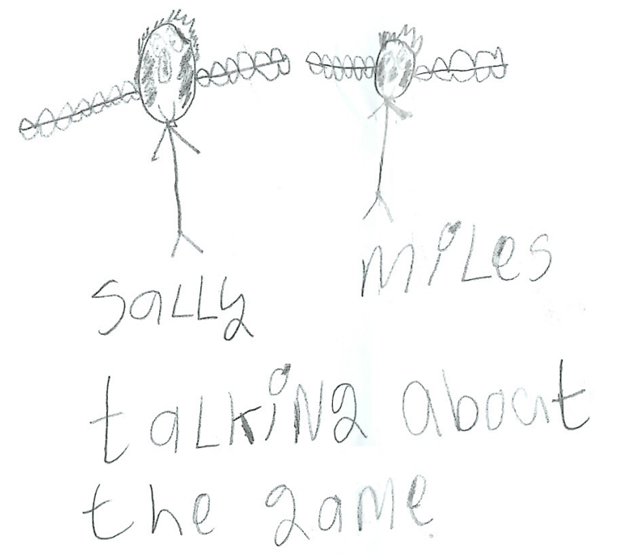   \|  \| \| --- \| |
| Good. Very knowledgeable, friendly, compassionate team. Well led and well trained and very experienced. Accepting of all kinds disabilities, focusing on the ability rather than the deficits. Bring the best out of an individual making them feel important, valued aqnd safe and secure. As a mother I felt very reassured and was treated with respect, compassion and understanding. My requests and comments were always listened to and discussed. Only some minor areas for improvement such as a more reliable telephone system for contacting care managers out of hours. If the office was not manned, the mobile numbers were out of reach of a signal. This caused some anxiety at times in not being able to reach someone. A few occasions of the internet being down. Residence could not get a phone signal. 100% recommendation and a million thanks for the opportunity and experience |
| Good. Alright, get on with everyone. I like going out with you (Health appt). I like going with somebody, I like someone with me (Health appt). Makes me feel better. I'd get lost without someone, its a big place. |
| My son has been living in a supported living complex for just over 4 years. He has become increasingly isolated during that time despite expressed concerns about this. Last summer his behaviour escalated further and finally he was referred to the ICATT team The nurse, speech therapist and psychiatrist from the team were all involved and over the last 6 months have assessed my son and dealt with his situation and anxious behaviour. Supporting the staff in the complex caring for my son has also made a huge difference. Supporting him in starting a small dose of medication to help reduce his high level of anxiety has helped more than I could ever have imagined and I’ve finally got my son back. Thank you Carla,Richard and also Nj for you kindness,compassion and fantastic input at (in my eyes) a critical time for my son. I’m truly grateful. |
| I would like to thank the staff part of the ICATT team from Highbury Hospital, in Nottingham (Steph and Adam) for their care and support of my son; giving him the confidence to talk about his feelings. He was looking forward to their every visit to express himself, something we have never seen him do before. Through doing this, they pulled us out of a very difficult crisis and placed us on a path to recovery by giving us strategies to manage his behaviour, support and advice which has allowed us to restore the order, which we have been seeking and pleading for, back to our home. Thank you so much. |
| IDD OT Healthy Lives group It was ok but not my thing |
| My wife is suffering from acute cancer disease at much advanced stage and recently shifted to her home. The support being provided under the district nurse Mrs. Durall and Billsley team is exemplary. She and her team are looking at the minute requirements of my wife to make her comfortable. She is always accessible to address any issue relating to her support. She even got involved in training of the carer team to deal with their caring responsibilities. Hats off to her and her team. |
| I have received incredible support from the Macmillan nurses, I cannot thank them enough for their kindness and warmth at this difficult time in my life. Fay and Rupinder have guided me through all the services I can get to make things easier for me and have made arrangements for social services to deliver equipment to the house to help me move around independently. They have made sure my emotional and mental well being is supported and have kept in constant touch with me. They have honoured and respected my wishes, I must say they are doing a fantastic job for a person like me, their kindness and words of encouragement keep me going. I also want to thank Barbara, we have not met but have spoken on the phone, she has given fantastic support, doing all she can to help me. Words are not enough to express my gratitude to the oncology department and BRI, who referred me to the nurses, and to the nurses themselves. |
| My story is about a bad experience I had when I went to Shipley health centre dental department the appointment was made through 111. It was a simple tooth extraction but lead to being a very bad experience. There was more damage done to the area surrounding the tooth which made it very sore and painful had to go to A&E for treatment aswell they took the stitches out which weren't helping. I had to take a lot of painkillers and antibiotics. |
| Below is a summary of some feedback provided by a service user who completed a feedback form provided to them by the Community Learning Disabilities Team following a period of care from that team: I was happy with where I was met for my care. The place I found the best to meet was my home. I think I was given clear and useful information, especially the advice around staying safe on public transport which I found the most useful. But I still feel like I need some help with this. I felt like I was treated with respect and I am happy with the help I was given. I would be extremely likely to recommend this service to friends and family if they needed this care or similar treatment. There is nothing I can suggest that you could do better. |
| Below is a summary of feedback from a carer of a service user who had received care from the Community Learning Disability Team – They completed a feedback form talking about the experiences of the service user: I would be likely to recommend this service to friends and family if they needed this care or similar treatment. I was treated with respect and I am happy with the help I was given. I was happy with where we were met for care and feel I was given clear and useful information. I don’t have any suggestions for any things the team could have done better. |
| Below is a summary from 3 carers who completed feedback forms sent to them following a period of care by the Community Learning Disability Team. They are talking on behalf of the service user that they care for and how they feel they experienced the care from the Community Learning Disability Team. They would all be extremely likely to recommend this service to family and friends if they needed this care or similar treatment. They felt that the service user had been treated with respect and they were happy with the help the service user had received. They were happy with where they had been met for the care provided by the team and felt that they had been given clear and useful information. They didn’t have any suggestions for any things the team could have done better and one commented that it was an excellent professional service. |
| The speech therapist was very helpful and the suggestions they gave me were very practical. Jo the physio and Chrissy both gave practical suggestions especially the kettle tipper - which has been very helpful. Just the advice to keep active bending and stretching were good reminders. |
| I am a man in my sixties with severe cerebral palsy. I live independently and employ my carers and have done so for nearly 30 years. Apart from physical difficulties my biggest problem is severe anxiety. To this end I use neuro out reach services, including physio, occupational therapy and psychologist support. These kind of outreach services are vital to people like me just trying to make the most of life and long may they continue. |
| We both found the staff very helpful Jo and Grace were very caring but flexible with their programme. They left behind them memories of two cheerful but professional young ladies. |
| The name is so evocative of its activity - "enablement service" - Brilliant! All therapists were highly competent, professional, and friendly. Having Parkinsons disease is quite threatening, so to be reassured and helped has been very positive, Thanks! |
| "ARC" was extremely beneficial: the physio sessions + walking practice with Tamsin, especially. I also joined the Hand Group; warm water + massage prior to completing various tasks helped to improve hand functions. After I left ARC, Tamara + her team assessed my abilities + introduced new therapies: bands, ankle weights, putty, showing me how they could improve mobility. Hydrotherapy sessions stopped after only three, unfortunately, due to budget cuts. Both of these services have made a difference to my progress + to my mood, by supporting + encouraging me to continue the exercises. |
| We waited each week for this lovely friendly lady to arrive. She saw us through exercises and arranged for a "three wheel walker" and a wheelchair to be ordered &amp; delivered. We can now go out with friends (when the wheelchair goes in the boot) and look round shops and visit cafés. Our lives are enriched due to her care and consideration. A big THANK YOU |
| I have used the Neurological Enablement Service at the Michael Carlisle Center. This service was very good and was a revelation for me. I just wish I had known about it earlier. The Neurological advisers should have a notice board or newsletter to inform people about this service. Everyone at the service was very good and supportive. I have been ignored in the past but I was listened to here. Unfortunately the centre is quite far from me and I cant travel on a bus. I had to get taxis. However once the advisors knew about this they arrange to visit me at home. The staff are quite young and they have very modern ideas. I find some older staff are a little set in their ways. |
| Jo did a very good job, she was very pleasant, very patient and was very encouraging for me. Many thanks to Jo & your department! |
| Having suffered from nerve damage to my feet I was referred by my neurologist to the Neuro Care Team at the Graves Sports Centre. Following my initial visit to the sports centre the therapists, Tamsin, Amy and Grace came to my home giving me exercises to do and checking progress. The care and attention has been faultless. When I was referred to them, I was unable to walk without bumping into furniture or able to stand without support even to brush my teeth. With their help I can now stand and walk unaided, even managing to walk across grass in Graves Park without my canes. I'm now looking forward to walking in the Lake District and building up the distances gradually and cannot thank the Nero Care Team enough for all their support. |
| The staff have responded rapidly to my wife's health needs. For example, when my wife developed a chest infection we received a home visit from the physiotherapist the same day. We have had our house modified to meet her physical disabilities and we have had excellent speech therapy making use of my wife's ipad. All the care has been well coordinated and I feel fully supported. |
| The experience was of no help whatsoever. I already know that the cough I had was nothing to do with eating or drinking. I have now received a copy of easy eating I am no further forward it to what this cough is about. I have had chest x rays and it is nothing to do with that. |
| The Neuro enablement service became involved with my husband when he was diagnosed with a Glioblastoma brain tumour. The team, especially Rachel the physiotherapist. Have been amazing in their support of both my husband and myself. I felt that I could contact them for support and advice at any time. Rachel always seemed to know before I did what help my husband needed next which enabled him to stay at home in comfort until the end of his life. Nothing seemed too much trouble and Rachel gave me the confidence to cope with any situation that arose. She was sympathetic and supportive always I cannot thank the service enough |
| My son is 10 years old and was referred to cahms by is school due to is behaviour problems 18 months ago. unfortunately I would not recommend this service to anybody due to the mistakes they have made they have made my son worse none of the staff who as dealt with my son knew what to do and he as been passed around from pillar to post even the head person could not keep a appointment with me. I was rung at 8. 00 am and appointment cancelled due to bad weather even tho everybody knew it wasn't going to get any better the day before so why he didn't call me before is strange. |
| I received outstanding service from this doctors practice. An automated blood pressure, height and weight machine has been installed within the practice enabling me to call in at my convenience to have readings taken. The machine was very easy and simple to use. This facility enables me to drop in any time that the practice is open, most importantly after work, and not restricted to a specific time. I could then hand in my results from the machine with a prescription request for my medication. My results had been checked by a nurse and my medicine was ready for collection from my nominated pharmacy within a few days. This service is fantastic. It gives me flexibility to drop in at my convenience. It also frees up precious NHS staff time and appointments for other patients. Thank you Mill Street Medical Centre. |
| Poor service, misdiagnosis and inadequate supports - 6.5 years of my life wasted. After being misdiagnosed with bipolar in 2015 I requested a further assessment as I suspected I had adult ADHD. After waiting over a year and a half, during this time I lost my job due to the side effects of the medication I was on for a condition I didn't have. Once reassessed and ADHD confirmed I was seen by the ADHD services once regarding medications and discharged back to my GP. After taking a reaction to the medication I requested to be re-referred to trial a new medication (as my GP couldn't prescribe). After a further waiting period of one year I was finally seen. During this time I was seen by a private psychiatrist who also diagnosed dysthemia (severe depression which was treatment resistant). I spent all my savings just to access treatment. The private psychiatrists reports were never used and not accepted by the GP practice - further delaying my treatment by 1 year. During this time I had to move house and my marriage broke down due to the total decline in my mental health and depression. After 18 months I am still awaiting treatment for my ADHD. During my last meeting with a psychiatrist they refused to prescribe due to the chaos in my situation. They actually wanted me to get my life in order to prove that it was ADHD! ! ! The psychiatrist's knowledge of ADHD was shocking considering They were working in this particular area, I challenged this strongly and insisted it was discussed by the team. The decision was overturned and I am now awaiting my next appointment to start treatment. My son has ADHD and the support and understanding within Ryegate is incredible and I have a lot of input into his care and treatment. Through SPA it feels as if I'm being treated as a mental health service user not a Neuro-developmental service user. Absolutely appalling experience which has had such a detrimental impact on my life - I've lost 6. 5 years of my life due to the absolute incompetence of Sheffield Mental health services, GP and Neurodevelopmental services. This has affected my son which is completely unacceptable. I've asked if they'd find this level of service acceptable if it was a member of their family, uncomfortable silence or deflection by all.. However the reception staff have been amazing and really supportive, professional and efficient, it's just a pity that it's not seen in the actual medical team. |
| After the my operation all was well until 6 days later in the evening I noticed a floater in the right eye. I phoned Probus early next morning and was told to go to Probus clinic at 11am. I saw the doctor who seemed very concerned when he examined my eye. He contacted Treliske hospital and I was told to go to the emergency eye clinic. I was examined thoroughly and was then told that they would operate and I may lose my eye and sight. The team were all so caring and kind and I went to the operating theatre. I had a long operation with key hole surgery, the consultant and the doctor monitoring me were excellent the an held my hand throughout. After the operation the consultant explained that he did all he could to save my eye as it was detached from the retina. He used laser treatment to try to connect it. He said that I will probably lose the eye but we will wait to see over time. I was then sent to the recovery ward where a lovely nurse looked after me when I could not stop crying. Then my husband was allowed to see me. After a while we were given lots of medication and I was told that I will have an appointment every day until further notice. I saw so many doctors, nurses and other staff during this time and every person was excellent in every way, they were so caring, professional and they all made me feel better each day, Nothing was too much for them that includes the reception and pharmacy staff as well. The consultant is pleased with my progress and if I carry on like I am by April I should keep my eye. This is thanks to everyone involved in my care. |
| Although the Somerset Partnership Trust like to portray the image of a wonderful and idealistic service, I feel the truth is far from it. I've been under their care for a long time to discover what I feel is incompetence and corruption within the service. I feel that cuts to services has led to failing of patients and lack of resources. I feel I have been neglected and failed many times. I have found that when I have complained, then the service is biased and do not like to admit mistakes. I had a very cold and distant psychiatrist who I felt failed me and I went to a private doctor as a result. The psychiatrist had very little compassion I felt I was treated like a statistic. |
| Office staff do not listen to our personal needs/requests. Carers are constantly arriving at inconvenient times. No matter how many times we contact them, they seem un-willing to listen to our concerns and nothing changes going forward. |
| My father was allocated to the end of life fast track team after wishing to come home to spend his last days. The service from start to finish was excellent the high key trained staff provided excellent care to my dad up to the last day. The management team were excellent and always on hand for support and advice and couldn’t do enough for us. They accommodated our preferences and sad wishes. Cannot say anything other than excellent. They gave our dad the most support and compassionate care we could wish for and we couldn’t thank the team enough. |
| I am very delighted to have Blessings care for me, I get to see the lovely girls and chat with them while they help me. The office lady is nice and polite. She listens carefully and is always happy to help. Overall Blessings is excellent |
| I am very satisfied with the care I get. The girls are nice too and happy to do things for me. They always ask what I like and all of them are nice.  They take suggestions on board and do things accordingly. |
| An excellent team of staff who manage to communicate and care for my mother with exceptional ability. Always on time and very professional. |
| They have a friendly approach and the ability to put clients at ease. Carers are tactful and sensitive at all times. Carers respected my mom and our family. They have high level of patience as handling my mom is hardwork and often stressful. I am very impressed with care2u. |
| When it comes to finding a carer for someone close to you, you want to get it right. If you're on the hunt for a high-quality carer, try care2u. One of the care2u carer's saved my dad's life when he had a nasty fall from bed by calling the emergency help on time and keeping him warm until the help arrived. She didn't move him but put a blanket on him, put a pillow under his head and phoned me to come asap. Thanks care2u for making our life easier. |
| When my husband got diagnosed with a terminal illness I became he’s primary Carer. My children insisted that I need some assistance with this and we decided to meet with a couple of local care companies. Out of the companies we met with Care By Us was who I felt most comfortable with.  The carers were friendly, caring and empathetic. They put all my initial reservations at ease, as my husbands condition got worse Care By Us support increased, we progressed onto a live in Carer who was an absolute god send, she remained with us for almost 3 years and I couldn’t have been happier with the level of care received. My husband was also very happy, content and comfortable until the very end.  I would highly recommend Care By Us they have an exemplary personal touch and I’ve always been dealt with in a professional and friendly manner by the carers and the office staff.  I would like to take this opportunity to thank all Care By Us staff for making an awful situation manageable, I really couldn’t have done it without you. |
| Are is absolutely appalling disgusted with the way they have treat a loved one. I have tried contacting managers on. Numerous occasions to complain but they never get back in touch. They don't treat patients as human beings there just numbers in there eyes and all they care about is money and ticking boxes. I know few of there carers work over 67 hours per week treat the workers like rubbish as well. This firm needs closed down... |
| I have great support people, but there is real concern from senior staff and support workers that the culture of the company is not working. I have been with Crimson Hill for 2 years now, and when things go wrong, the 2 joint directors who are related ignore complaints made. Some really good support staff have had to leave because of concerns. I put in a complaint 8 weeks ago and again 4 weeks ago because I was left without care and support due to staff leaving and I almost lost my life. They did not seem to be bothered about this and the social worker had repeatedly asked them to restart my hours. They do not listen to the county council who pay this private company, even though they do not provide my commissioned hours, it's not the way to treat people. I have had only limited contact from a support worker by phone. I have had no response at all from the director since December. She will not answer the phone or emails. I am at a loss at how this company is still in business. They need to respond and communicate better. My experience since December has been very poor. I hope they start to listen to concerns and complaints in order to get back to helping people. The company had a good caring reputation back along but lately for whatever reasons they do not seem to be maintaining this. I hope, they can deal with complaints to put things right in a more orderly fashion and have a complaints policy made up so clients with learning disabilities and mental health needs can be helped without fear of not being listened to, and to be treated with dignity and respect. |
| I would not recommend this agency for care in my view. Lacking leadership  Lack compassion  Lack of communication  Lack of support staff Based on my own experience 0/10 |
| I experienced a bad attitude from some of the staff at Crimson Hill Support Services Limited recently. I asked them advice on behalf of one of their service users who was terribly unwell waiting out all night in freezing temperatures. They were unwilling to help her, were totally unhelpful and some of the attitudes from the managers that day were considerably hard to understand considering they were from the caring profession. It made me worry, although we tried to take care of her as best we could. I would not expect this of a private company and was pretty shocked by their lack of kindness. |
| I feel unheard and I listened for the lack of thought or care provided by Crimson Hill Support in North Petherton. My support workers are wonderful, but when I became really unwell lately, they withdraw my allocated support hours without a reason. My support workers raised concerns with the private support company and neither of us were listened to or given any proper answers. I have deteriorated badly since they pulled my care leaving me to struggle and manage without any explanations from the care company. To leave people unable to cope to the point of crisis is in my view really poor management. The company are employed to take care of people with learning disabilities and mental health care needs, but just seem not to listen or want to put things right when things go wrong. They seem to blame the person, rather than meet to discuss ways to amend. I would not recommend this company to anyone I know, until they put right, sometimes serious concerns. The support workers I spoke to feel they are unheard, and not taken seriously and ignored making them wish they could leave. It is unsatisfactory. I hope this company can learn from listening in the future, to help improve patient experiences. |
| I felt very let down by Crimson Hill Support services limited recently. They did not give my mum her allocated hours when she became unwell. They refused to explain why. The director and manager would not explain why they had let her down, and that they were awaiting a meeting before continuing her care even though they were still contracted the hours. The last 10 weeks have been a massive struggle with no end in sight and no explanations from the director or managers. We gave up calling and asking as they just wouldn't respond. After the meeting they were supposed to answer our questions and start support but theh wouldn't communicate or give answers. Their lack of communication and lack of care made our mum even more unwell. We now find it hard to trust them, as there was no reason why they did this to us. We feel they should listen more and take on board what worries family have and what worries care support workers have as they are the ones who support most. |
| I was a service user being supported by Crimson Hill Support. They are a private company run by two directors who are related. They look after people with learning disabilities and complex mental health needs. Recently due to unknown reasons, they did not supply my assessed hours. The social worker repeatedly told them I needed my care. Since December the director has refused to reply to my formal complaint and is always out when you ring or go over to the office. Her car is parked outside and the support workers who are brilliant wont get listened to and feel helpless. The support workers, the good ones leave because the management think they are above everybody and do not get listened to either. I have support workers who come around in their own time to help me but were told by the directors, that if they did this they would be sacked. The company do not listen to anyone or respond to formal complaints. They cut your support and dont give adequate reasons but instead ignore. I went to their office, but they locked the doors and refused to answer the phones. I nearly died a couple if weeks ago without my support from them. They ignored this. No compassion care or leadership. The culture is bad to say the least. They told me if I came to their office to ask for help in the future, that the NHS told them to call the police to arrest me. I am very unwell at times and do not need this. I have not done anything wrong I just want the director, who I deal with to answer my messages and my formal complaint as to why they cant put things right. We used to get on really well, I was always taking them over presents and had a good relationship with all of them. They have definitely gone down hill as a service since they had management changes. |
| My mum used Crimson Hill Support as she had complex needs. The service was not great. We feel they have let her and us down in respect of her care from them. She has not had the allocated hours and although they always have not got enough staff, we were left dangling with no communication. Weeks and weeks without care or no contact. It was not good. Over Christmas and New Year and all of January and February, no answers to our questions. We gave up as there was no point in contacting them. The managers are away. No complaints procedure. The secretaries and other staff kept promising to pass on messages but it made no difference. If you complain to this firm they think you're challenging them. I wouldn't hold much hope in this care agency. |
| I had been poorly in December and found that the compassion was withheld. I was denied treatment for being too unwell. They said they were waiting for other professionals to help them, but I believe this was an excuse because there was no other professionals involved. I was waiting with no help even though I was supposed to have it. Crimson Hill managers decided to halt it continuously with no promise of when they were restarting it. We were not kept informed. They did not give any indications, just would make it up. To date, the director still will not communicate to us any plans or weather she will let happen again or why. I still have not got full hours. They have their own rules to suit. They have no polices online or in paper form to tell you how long a complaint will take. They have not kept their promises. They stall. I am not impressed by the way they manage the company. It has shown a lack of compassion and understanding which I think a care company working for people with learning disabilities and mental health problems should acquire. I haven't been impressed with lack of communication skills. It may be due to the large quantity of staff who are leaving and starting, I realise they have problems. But even so, they should be willing to talk. |
| Over the last 3 years I have been detained in 3 psychiatric hospitals and before that I have been in and out numerous times due to my weakness for drugs and non compliance with medication. I would describe some of my behaviour as evil in terms of aggressive and abusive behaviour toward the staff and patients in many hospitals but more so at Cygnet Derby Low Secure before I was moved to Cheswold Park Medium Secure Doncaster. It was easy to obtain drugs in Cygnet Derby and to a certain degree I sincerely believe it was turned a blind eye to. Personally I feel that as concerning as that sounds it is part of the attitude I have today in my acceptance that I need to abstain from what I have been advised for years causes me to become ill. At Cheswold Park under one of the consultants I was given a medication free trial at my request and became ill. After a brief time in seclusion I accepted I was ill, recommenced medication and after a couple of changes and reductions at my request and increases with the Drs advice I made the turnaround that enabled me to move to Low Secure at St Andrews Northampton. I put my current attitude down to such person centred care from the MDT at Cheswold Park which allowed me a medication free trial that I have requested for nearly 10 years beforehand. After that I feel far more confident that the medication I am prescribed is beneficial to me as well as the advice to stay away from illicit substances. At Cygnet Derby I wrote a book and used my benefits to save up and start the process of having it self published. I'm still on benefits as the book isn't successful although I'll keep trying. At Cheswold Park I was enabled to finish having it published through use of the Internet and communicating with the publishing company. I was told at my assessment before coming to St Andrews that I would be able to use the Internet to continue communicating with the publisher of my book and potential future publishers. When I arrived I was told that it wouldn't be possible because of Internet restrictions. I have Internet access now in the form of a smartphone although not by simple reasoning. I had to write a complaint and a smartphone is better than nothing but I was still misinformed otherwise I wouldn't have accepted the bed here. In some ways, St Andrews Low Secure is more restrictive than Medium Secure at Cheswold Park and definitely more so than Cygnet Derby. Not in terms of the availability of drugs but in terms of Internet restrictions and Section 17 leave too. To summarise, what has mainly worked for me is that my opinion in regards to my treatment mattered at Cheswold Park. My request for a medication free trial, to have my meds changed and reduced led me to no other choice but to listen to the RC and MDT's advice and opinion. Being able to get drugs at Cygnet Derby and messing myself up so badly to be moved to Medium Secure has given me experience of being almost nice to be around since being clean as well as occupying myself productively, along with the embarrassment of so much of my past behaviour. Although I can't condone being misled into coming to St Andrews, intentionally or not, or the restrictions, I have been able to put a lot of what I have belatedly learned into practice. I haven't been given any risk behaviours for being inappropriate, overfamillier, rude or insulting in any way. If anything I have learned a lot about the benefits of patience and tolerance and dealing with my frustrations in a positive manner. Whatever the intentions of any hospital it is better to get out and stay out. There is far more to be achieved, far less restrictions, necessary or not and although I would prefer to be at another hospital than here that hasn't misled me or is so restrictive I'd rather have my freedom. |
| I had a biopsy on 12th May and was told my results would take around 4 weeks. I have called every week since week 4 only to be told they do not have my results and to "call back in a weeks' time and we should have them", which they have not!! The issue I have is getting worse and has started spreading over different areas of my body, which is causing me much distress. I have to call back this Friday 13th and I guess I'm going to get the same answer....!! |
| Called dead on 8am, 30+ people in the queue. Was cut off after 20 minutes. Called again and used the request a call back option. Didn't get one. Called 111 who also tried to call the surgery and couldn't get through. Surprise, huh? Was sent to the surgery by 111 woth an urgent request to be seen, casenoted had been sent. The receptionist refused to look at the case notes, or even ask a name. Was very bluntly told no appointments. Go to urgent care. But you need a GP referral for urgent care. Go figure! So now, 7 hours after first calling, my 3 year old still needs to be seen, everyone has refused so far. So now I have the choice between waiting for MEDOCC to open or wasting precious a&e services. Absolute shambles and I'm completely fed up |
| Mum been here six weeks now, cant rate this place highly enough, staff superb nothing is too much bother always willing to help or answer questions. Care is well planned and executed and mum always says how lovely all the staff are and how helpful. Food looks good and is always eaten with variety and sweets after lunch and dinner, and drinks are brought round on a regular basis. Very happy with the home, staff and management. |
| Several abrupt carers but also some pleasant ones. Care Home Manager nice but the place needs an overhaul and some attention from the owner. You can’t expect the most from your staff who work hard, if you’re not bothered. Happy to take the money but definitely no frills here! Look elsewhere. |
| The staff are rude, and are inappropriate with clients, smoking with them. Bad care/service overall. Do not recommend this service. |
| I have had a few carers from ena and gotten on with some more than others. However, the level of care and support I receive at home is commendable from them. I feel supported at home, especially as each of the carers I have had have been competent to care for me at home and my care co-ordinator is a nurse who has often been an immeasurable support to me. I would recommend ena to others. |
| Dad has been living on his own and took a nasty fall over summer. Hospitalised which seemed to take a knock on effect. Trying to get him home was a headache but found the team at ENA who were very supportive. First few weeks felt like was constantly ringing them. After a while it has settled and although dad has taken a while to come around to the idea and it has taken a few carers to suit him, he is doing much better,.  Having someone live with him has improved his quality of living. Couldn't recommend ena enough. |
| Without exception my husband has experienced nothing but kindness coupled with exceptional care of his person, he looks forward to chatting with his carers. |
| Very friendly and supportive staff and nurses. Providing care for relative with dementia. Would certainly recommend services. |
| Always ready to help at the drop of a hat and very caring. The lady who comes to help my mother is extremely good and works very hard. My mother feels very comfortable with her. we will be increasing the amount of care over the next few months and I know we will have the same experience. They have made mum more secure and are enabling her to stay in her own home for longer.  Every Care also looked after my father 6 years ago before he died and the experience then was exactly the same, kindness, care and time, the 3 most important aspects. |
| Over the years its clear that Everycare have a good base of efficient friendly and caring front-line staff.  Most importantly they always make the effort to support understand and respect my Mother, who, as we all do, get older and sometimes become frustrated that we can no longer do what we used to be able to do. |
| very sensitive carers who have been coming to take my wife, who has dementia, out for walks or visits to a garden centre or for coffee, and prepare lunch and supper etc. |
| Everycare Central Surrey have looked after my father for six years now. In that time we have experienced their skill at caring for and bringing my father back to good health and happiness. They combine excellent, genuine care with continued organisational excellence which I believe is not easy to strike such a great balance. I couldn't recommend the care manager and his wonderful team more highly . |
| My husband has early dementia and Everycare have been very accommodating in helping with his care, taking him for nice outings and making life a little happier for him. The carers are very sweet and caring and seem to have an infinity with someone who has the sad disease of dementia. |
| We are very satisfied with the help we receive as they are happy to adapt to our needs, and my husband enjoys his social outings. It has a family feel . |
| Very willing to adapt to our needs. Efficient service with dependability and very friendly and caring help. Always easy to contact. |
| All the staff from carers to administration and Managers are a credit to Everycare, Merrow...at all times they are adaptable; cheerful; courteous; emphatic: enlightening; friendly; helpful; genuine; harmonious; kind; patient; positive; reassuring; thoughtful; professional; understanding; versatile; warm and wonderful throughout. Mum has a regular morning carer and the brilliant team of carers always cheer her up. I highly recommend Everycare!! |
| They can be flexible if needs be, do anything required, always friendly and supportive. They tend to figure out what you need before you ask, are proactive and enthusiastic about going the extra mile. Timekeeping is taken very seriously and usually if they come late, they stay late. They always let you know when they will be late, because they are excellent communicators and very reliable. |
| I found the staff very caring. They were always very prompt and if there were any problems and I was always informed. |
| Care plan not adequate, assessment is for physical problems only. Don't have any provision to record how to deal with a persons mental health problems i.e. if they have memory problems. Care plan didn't include everything. Have been having to leave notes all over the house because of this. Have been having problems over the cleanliness of their work. Don't really know how else to phrase this. The owners appear to do what they want. Some of the carers appear to be doing the work while others don't seem to be doing what they should. They appear to take advantage of the person they care for if that person isn't able to remember. When there is serious issues I don't feel that they take them seriously. I'm so desperate for the help I have no choice but to use them because they are the only company employed by Social Services. I don't understand why they advertise that they do dementia care because they simply have no understanding. I have no idea how the CQC can even give them a rating of 'good' it seems a very strange appraisal of them. |
| the care people who come to my mum are very friendly and cheerful.They are always helpful and treat my mum with respect. |
| My brother used Helping hands as they said they could meet his requirements. Very sorry to say this "family run business" did not care about my family or him the Terrible management the lack of care coordination and of person centred care .He was always an   afterthought the last to know he had very little say in who he worked with and the matches were shocking. this company would hide behind newly created then scrapped policies and the care plan which took 9 months and was still not correct after he left. The training which is apparently ..award winning after speaking to carers when visiting him was not there .As carers were clueless about his conditions and he felt like he was being treated as "different" and odd not nice to cope with mentally as well as his a progressive illness ,really shocking lack of professional standards .staff would talk so much nonsense unrelated to the roles .some very far right opinions which perhaps he could of coped with if he was not anxious as this was not taken seriously by the manager. .Hygiene was an issue constantly finding staff had not cleaned properly . greasy forks spoons plates , alarming the state of the bathroom the carers would leave it in.emails were sent out by the manager but when new staff began it was not relayed by them to new carers dire communication. .although from the start Helping hands were Informed little to no effort other than a few fact sheets as an after thought after we expressed our concerns as a family. no continuity of care .carers would often assume he lacked capacity as not read the care plan fully which was never updated in his home fully and branch were not supportive. .the senior manager i can only describe as uncaring abrupt and target driven JHB type. terms and conditions policy are definitely worth reading as well .  .discussion of other clients and staffs own personal and health problems was routine and he found distressing . . The only thing i can say that was good about helping hands was the marketing .I would Avoid this company at all costs. |
| As a current client I am not impressesd. My experiences match those negative reviews expressed on this site. I am very vulnerable, but this makes no difference to my local branch. The complaints process is very difficult to navigate, and nothing seems to get resolved. After 11 months with the service I can only say:expect confusion, expect no shows, different faces everyday, cancellations on the day and little concern about client views or needs. |
| My mother used helping hands for about three years. I Visited three times a week very often she was lead in a bed of Wee. Very often they haven’t fed her properly or given her medication. She had 24-hour care on occasion I found the night carer asleep on the settee with a pillow and duvet and in her nighty,(what a joke).Also the management is very poor just backs the carers they said i shouldn’t take photographs of the carer sleeping  Even though they should be working. If you love your family don’t use helping hands. |
| Our family have a combination of both hourly visiting carers and live in care from Helping Hands to look after my elderly sister. The teams have worked tirelessly, often going above and beyond to ensure that she is as comfortable as possible in what at times have been difficult and challenging circumstances. The services work well together and we have clear and regular communication with the relevant managers.We cannot thank them enough and would highly recommend Helping Hands to other families who find themselves in need of professional help and peace of mind. |
| My siblings and I were very pleased with the care provided by Helping Hands both as an organisation and in terms of the carers who looked after our parent. There was a comprehensive assessment both before my parent came out of hospital and on the day they returned home,when the care package started. The manager also kept in contact with us and the carers during the contract.  I was impressed with their professionalism and with their courtesy, tact , compassion and hard work. We had 2 live in carers who alternated between them for several weeks at a time. They both worked hard to establish a good relationship with my parent and also to establish a good working routine. My parent's needs were quite considerable as they were bed bound and terminally ill. There were multiple medications to be administered daily both routinely and as required. They took care of all their physical needs and also provided good company to my parent which helped keep their spirits up at the most difficult time. The carers were helpful, responsive, sensitive and compassionate. They knew when to step up to the plate but also when to step back and allow family members to help. We did make sure that the carers had our phone numbers and that they would call one of us if they had any concerns, which they did, when they needed to. They were also professional in dealing with problems as they occurred and also in liasing with other health care professionals. My parent had many friends visiting during the final weeks of their life and the carers were always friendly and hospitable, many of the friends commented positively about the carers to us, and one couple asked for the name of the agency as they had been impressed and wanted to recommend them to someone. We really couldn't fault them, and I would not hesitate to recommend them. |
| Helping Hands have been supporting my grandparents for a few months now and they've been nothing short than outstanding. What initially started off as extra support with cleaning, washing, changing the beds and making them meals, has now turned into daily support for their personal care and helping them to get out and about in their local village. It's taken a huge weight off the entire family and we can't thank the team enough. My grandparents always sing their carer's praises and it feels like they've gained friends too.  And it's not just the care that's been second to none. The whole process from looking on their website and getting in touch with them on the phone, to having a meeting with a manager has all been done with such professionalism, kindness and ease. Even when my grandparents added extra calls at the last minute for more support, the team always accommodated them and always go above and beyond. Thank you Helping Hands for everything you have done. Knowing that my grandparents can stay in their own home rather than move into residential care means so much to all of us. |
| Instruction for care visits arranged by myself at very short notice - all accomodated. Good communication, easy application forms and financial arrangements. My relative is very pleased with the friendliness and efficiency of visiting carers. |
| Live-in carers varied considerably. Personal care, which was complex, was always good. The additional support eg. getting meals, help with light housework, was variable. None of the carers was able to drive and one didn’t cook! There were difficulties with privacy for my wife and I to be alone for a time in the evening despite requests for this to happen. Over a period of two and a half months, we had three carers due to a planned holiday for one and a decision to return home by another.  The carer who lived in whilst my wife went on holiday for two weeks was excellent in all respects. |
| Helping Hands came to the rescue when two other care companies had withdrawn in succession, from providing support to enable my parents to remain living in their home. We were recommended HH by our local community dementia nurse and a representative from the Alzheimers Association. The Gloucester office set up a meeting during which detailed care plans were drawn up for both parents- this had not been done by either company we had previously employed. The carers who call, whilst inevitably having different skills and abilities, are capable and show initiative in observing when something needs doing without being asked. I provided a detailed list of how the household runs to help them settle in to the routine, though some of them had not had time to read it before starting, all soon caught up on what was required. We fitted an outside key safe and each carer lets themselves in to start their session.  The communication between the co-ordinator and our family has been excellent, with a weekly list of who will be calling sent to us all. Any enquiries we have had have been dealt with very quickly. There is no doubt that without HH twice daily support our parents would not be able to stay in their home. It is hugely reassuring to know that as our parent's needs become greater, they will have compassionate, understanding and capable help from HH's carers. We are all very relieved and extremely grateful. |
| My carer was friendly, kind and very helpful. Being disabled and virtually house bound it's a great relief to have someone call in and check up on me to make sure I'm ok. It's also a great relief for my wife when she's away visiting her family. The services provided by your Lincoln staff are impeccable and I shall definitely use your services again so we continue to have peace of mind. They are also extremely prompt and always arrive at the agreed time. I enjoy our brief chats and their attention to detail. |
| Moved my mother from live in care - excellent- to day care - truly appalling. Unreliable, dreadful time keeping if they turned up at all. Would not recommend this company at all. Unreliable, unhelpful, uncaring I could go on. |
| Having chosen Helping Hands to help with 24 hour live-in care with Mum 18 months ago, we have nothing but admiration for them all. They are very good with communicating and organising care, making sure that all the carers are a good match to Mum. Helping Hands obviously train all the carers the same way - they are kind, supporting and gentle. I feel totally relaxed now, knowing that Mum is being cared for whilst I am able to get on with my very busy life, but get to see Mum now for the quality times. |
| We have had hourly carers in for my great auntie for about two months now. Every carer who has come in, have been on time, very polite, caring and gave my great auntie lots of choices for meals and nothing was too much for them. I would 100% recommend Walsall branch. |
| Our daughter recently moved into High Hurlands and we have been extremely happy. Her transition was planned very well and all the staff were so welcoming, caring and helpful to us. Her room is beautiful and all decorated before she arrived. She has a full week of activities and day trips out. The care she receives is first class and she is so happy since moving into her new home. Communication is excellent with the nurses/care workers and we are always made to feel very welcome when we visit or phone or email. We are always told about appointments and updates on how they went and we feel very much a part of her care still. I would definitely recommend this care home. |
| My daughter has recently moved to High Hurlands residential care. The move went very well and I feel that staff facilitated this move extremely well. I feel very welcomed when I visit and I feel that my daughter is receiving an exceptional level of care. The staff are in constant communication about my daughter and I feel enabled to approach them about any matter relating to my daughter's living situation and daily care. Overall, from the short time that she has lived there I only have an extremely positive view of her care and living situation. |
| Our daughter has lived at High Hurlands for 3 years. This nursing home provides excellent care, communicates effectively with us about our daughter’s progress and has kind and considerate staff. Our daughter recently attended A&E in the early hours of the morning. A member of staff from High Hurlands stayed with her until 5 am and made arrangements for another member of staff to return in the morning. This was despite the fact that I was in attendance and demonstrated the care offered by the team at High Hurlands. I would have no hesitation in recommending this nursing home. |
| High hurlands provides at most care for  special care children and Adults. They’re professional in their management and also whom they take for employment and their training is vital before hand and is not taking lightly. All the stuff are caring and hard working and always so welcoming. |
| My son has been at High Hurlands since 1984 and he has received the most amazing care over the past 36 years. All the staff are fantastic and the facilities are superb. Every resident having their own bedroom with overhead hoists if needed. The facilities include Hydrotherapy, swimming in lovely warm pool, pottery, arts & crafts, music, very large gardens with beautiful views of the South Downs.  Very well maintained buildings and all kept scrupulously clean. Highly recommended for anyone with severe learning and physical disabilities. |
| High Hurlands is now a home from home for my daughter. I visit every week, and I know she is being well looked after. Communication is excellent and if I have any concerns they are always acted on |
| This home is very welcoming and extremely well run and all the staff are very kind and helpful and the home is always clean and organised.  Our daughter has been here for 5  years and she is very happy here and her care has been consistently of a high standard.  We are kept informed of any changes and have regular reviews and contribute to her care with our views being considered.  My daughter enjoys all the varied activities that are provided including the on site facilities including a sensory room and hydro pool along with an art and pottery room.  What is also great is that she has opportunity to go on holiday in a small group every year. We are very happy that she happy and we can visit when we like. |
| I am 62 and 95% bed bound and I have Bipolar. I self harm and I have also tried to commit suicide a couple of times. About almost 6 years ago, I was assessed my Brighton and Hove council for a care package. I have 1 call of 90 minutes a day. By an a care agency. I also had a 1 hour shopping call and a 2 and a half hour domestic call per week. It worked very well. I also have COPD and a skin condition, where I bleed. So, infection control is vital. For chest infections and skin infections. Any way, I hadn't had a review, until a couple of months ago!! Yes!! 6 years! I had a student social worker who did not make a note off anything I said!! No notes at all!! I am hard of hearing, which I told them. They were too busy talking to someone from my agency and I could hear nothing that was being said. Next thing I know, a letter arrives informing me my shopping and domestic calls were being taken away. With 2 weeks notice. I could employ a cleaner. What with???!!! And I could shop on line. fraught with issues! waiting for rota and then trying to book a delivery slot when my carer would be here to take delivery and put food away. I lived on what was in the fridge and freezer, then I tried. Impossible, no available slots. Meanwhile the house is getting dirty, as is my bedding. I, took photos. so far I have had 2 falls and broken my ribs and had 4 chest infections. I said it was a neglect safeguarding, Issue AGAINST the council. Of course, they didn't agree. They don't care. They expect my carer to do the housework. They do all my person care and cook my meals, they are not superhuman! Council have said that, Now I am supposed to go to the Ombudsman. I have no one to help me. My head is spinning and my mental health has taken a nose dive. On top of this I've just had to have my 18 year old cat and 14 year old dog put to sleep, because of severe illness. I, just feel I can't cope.. [Very unhappy with 'Daily living support'] [Very unhappy with 'Involved in decisions'] [Very happy with 'Care staff'] [Very unhappy with 'Being listened to'] [Very unhappy with 'Pain relief'] [Very unhappy with 'Cleanliness'] [Very unhappy with 'Staff attitude'] [Very unhappy with 'Access to outdoors'] |
| Farndale has cared for two of my family members and they have given an excellent care service. They really care and we feel we are a part of the Farndale family. I can Highly recommend this fantastic service. |
| This wonderful team should be highly commended for providing an invaluable service to my Dad after hospital discharge. They are a fabulous team of ladies who care without being patronising. My Dad has recently spent his 90th birthday in hospital but is fiercely independent. They are enabling him to regain that confidence with respect and dignity. I can’t thank them enough, true angels in disguise. |
| My son needed a new boot for his fractured foot, because of being isolated due to the coronavirus a staff nurse at Salisbury District Hospital kindly offered to drop at our home. What an incredibly kind thing to do, many thanks |
| his is an outstanding care home (and I have seen many in my professional capacity). From the moment my mother arrived she was treated with respect, dignity and above all kindness and tenderness. In the twenty months that she lived at Antokol, I never witnessed any of the staff treat any of the residents with anything less than gentleness and attentiveness, despite the fact that some of the residents have dementia. The residents are treated as individuals and activities are tailored to their particular needs. There is an atmosphere of calm efficiency fostered by the excellent leadership of the manager. The home has mainly Polish residents but the English residents and their families were equally happy with the care and had chosen the home for its caring ethos. In the last days of my mother's life the staff treated my mother and us with exceptional solicitude and care and we could not have hoped for a better place for her to end her days. |
| MY FATHER SPENT THE FINAL 18 MONTHS OF HIS LIFE IN THIS OUTSTANDING CARE HOME . THE " TRUE CARE , FRIENDSHIP AND LOVE " GIVEN BY ALL THE STAFF WAS WAY BEYOND ANY PRICE . WE EXTENSIVELY RESEARCHED BEFOREHAND AND WITH DADS FULL INVOLVEMENT AND AGREEMENT CHOSE THE ANTOKOL CARE HOME . THE SERVICE EXCEEDED OUR EXPECTATIONS . THE CARE GIVEN TO ALL RESIDENTS IS BEYOND MEASURE , EVEN IN THESE VERY CHALLENGING TIMES . WE HAVE NO HESITATION IS RECOMMENDING ANTOKOL AND THIER AMAZING STAFF . CARE , WAY ABOVE ANY PRICE . THANK YOU FROM THE BOTTOM OF OUR HEARTS . XX |
| Extremely difficult to get an appointment here when you need one.  The one time I've been ill over 2 years, was given a course of antibiotics that was too short, resulting in illness returning immediately. The Dr I saw in subsequent 2nd appointment was condescending, and offered no further advice for pain relief (for a condition that has now persisted for over a month).  Receptionist on desk was being loud & rude to other members of the public regarding prescription issues.  Poor communication - patients are not informed that their own GP has left the practice.  Complete disregard for the welfare/health of the people who are patients here. (which is the norm for this practice) |
| This used to be such a lovely surgery, where the Doctor actually knew you, your medical history and cared. It has become so hard to get an actual appointment (let alone with a GP you actually know. The experience of visiting what is meant to be 'my' GP has become ridiculously difficult and impersonal. I feel genuinely deterred from attempting to go unless necessary and on the few occasions I have, the appointments I am offered can be weeks later, which, if unsatisfactory you are told to go to the walk in or A&E. It seems as though this practice is invalidating itself. Whilst staff seem pleasant enough, the point of the practice is to see your 'own' GP in what is a timely manner. I have certainly not been told (or written) to regarding GP changes at the practice or any new staff, which leads me to believe the practice is genuinely not interested in what the patient experience is at all. |
| My relative was cared for at the end of her life in a very caring, kind, compassionate way. We cannot thank the staff enough for the wonderful care and support they gave to our relative and us as a family. The Home is lovely from the moment you walk into reception and greeted so warmly. All staff work so well together in whatever role they have, to do the very best for people living in the Home. We highly recommend Romford Care Centre for anyone looking for a care home. |
| Warm and friendly staff who evidence kindness, compassion and respect  Contemporary environment  Great social occupational activity on the day visited excellent customer care |
| This service is excellent. The team are amazing and go the extra mile for the people they support. Certainly a bright future with autism research too. |
| Holly Bush and its staff provide the greatest standard of care that we could possible want. You all went over and above in changing schedule to accommodate our son and also making us feel reassured and relaxed that we were leaving our son in your excellent care. Our son has many complex needs and for us personally we struggle to leave him in anyone's care as we don’t always feel comfortable but you have changed this for us and we will always be so grateful that we were able to go away and not worry about our son's safety and wellbeing which improved our holiday.  Your initial visit to our home and access to our son’s needs put us at ease. Your professionalism was outstanding but also personal. When we bought our son to you, you were prepared for his specific needs and were more than happy for us to go through his routine in minute details. Your staff are so caring and provide a personal experience so wonderful not just for our son but something we felt ourselves. They were polite, responsive and courteous and the management also took the time to speak to us when we visited and were interested in any comments we were able to make. Our son was so happy with you that he is looking forward to returning. |
| I visited my sister during the nursing home’s Christmas party and it never seems to amaze me how much effort the staff put in to make this day very memorable for my sister. There are plenty of staff available to meet the needs of my sister and the management are always available when I need them and are quick to inform me if there are any issue regarding my sister. |
| I was supported to gain more confidence in my everyday life. I had been at such a low point in my life that I didn’t want to go out, see anyone, or even stay alive on some occasions. The help I received was invaluable and helped me to open up and see a more positive future. I was shown tools to work with that enabled me to start to change my life around. I also received help and support to be able Try to gain employment. This was supposed to be the main focus but I wasn’t in the right place mentally to be able to do this at first. The service has been a very positive experience for me. I felt that Jennie in particular has been excellent. She is so compassionate, non judgmental and shows such empathy towards what are at times very difficult emotive problems to deal with. Her problem solving ability has helped me to move forward in life and I would like to say a special thank you for the help provided to me. I would recommend this service to others as long as you are willing to engage and help yourself too. On the down side, other issues were brought to the forefront which needed to be dealt with but because it didn’t involve actively seeking employment at this time, the service has been ‘pulled’. I now have new major issues to deal with though and feel that my support has been ended too abruptly. I am now left to go ahead to manage alone and it’s looking very dark ahead indeed. |
| It's better than job centre because it is more specific to Autism. My coach has helped my write a better CV and cover letters and helped me get to an interview. She talks to employers to inform them about Autism and adjustments to work in the workplace. She organised a social group because I wanted more social interaction and I really enjoyed this. |
| It's a really good programme. It is opening up opportunities for my son in the world of work. I feel as though we are in a place where he can succeed in learning new skills. There is a good level of care and understanding. |
| I get good help and advice about looking for work that is suitable for me. My coach has helped me fill in application forms and online tests for different employers. We have updated my CV and written different cover letters. My coach helps me to job search and we look in different places online. She has taken me to Job Fairs. I feel as though I have been supported and am more confident in job searching and applying for jobs. |
| The services from the company and by all the staff were helpful and useful for myself and others in the future to come. Telling staff about the good news about the interview at a bakery was successful and offering me a 2 hour trial tomorrow. |
| Autisum Plus have been really helpful and I feel like I'm being looked out for. The service for me doesn't need improving. I feel very satisfied with my service so far. |
| My experiences with BBO have been good. I have enjoyed working with Alex and Lydia. Unfortunately another member of staff left half way through but Alex has done well at stepping up to the mark. |
| I would like to say a massive thank you to BBO and SYHA/Crisis for literally saving my life and restoring my faith in people. Liz was my first health and well being support work and she was amazing as she left her replacement was Alex. I said I did not want to see her at first as it takes long for me to trust people and had made a bond with Liz and this was very unfair of me as Alex is great. I believe BBO is a life changing opportunity for people who need a bit of help and reassurance. Through BBO I also met Lydia who is a job coach and brilliant at what she does she has helped me be confident about my achievements and helped me believe in myself as I have very bad depression and self confidence in myself. Thanks BBO. |
| Hi just like to say thank you for everything what lisa & emiley have done theve been a big support & help thro my tuff times they are a credit to the company i always felt relaxed when i meet them i would like to say a big thank you |
| Emily and Lisa are helpful, they are trained to help me back into employment. I feel better for meeting them. I would feel helpless if I had not had their support. I am on a COSHH course thanks to Emily and Lisa. Emily is going with me to the enrolment as I wouldn't be able to go on my own. Lisa is applying for jobs for me. They make me feel more positive. Thank-you very much to them both. |
| Thankyou Emily for being kind and lovely and making me feel better. Emily has helped me with my mood and anxiety. She has helped me get out and about and I am now going to volunteer at a food cafe while I am finding work. I met with Emily today and bumped in to two of my old friends at the same time, it made me feel so great. Lisa is helping me find work and I had an interview. She is helping me enrol on to a teaching and education course which would be so good for me. They have both been so supportive and it's been nice having support from people when you feel alone. |
| To whom it may concern I went to the job centre with my grandson and a lady came up to him saying about being able to help to get a job. I thought she was only saying things and would not be able to do anything. How wrong I was! She has been brilliant, got him a job helping him all the way. I know now its Lisa and Emily. And he can phone her with any problem. He has now got a jog and work clothes. I hope this scheme carries on to help others alike my grandson. Thank you to Lisa and her Team. Well Done |
| I have been visiting All About You (BBO SYHA) in Sheffield for approximately 2 months. The help and support I have received from Lisa has been amazing, what a thoughtful and caring person she is. I am lucky to have such support from a great team. I am always made to feel welcome and am listened to intently with no end of suggestions of how to progress into employment,. Lisa is in regular touch via email with jobs she has found on various websites. I would be extremely upset should this service not be available, I have been given faith in myself to have a can do attitude again, something I am reluctant to loose. [Very happy with 'Keeping in touch'] [Very happy with 'Seeing the whole me'] [Very happy with 'Finding your way'] |
| I started the BBO journey at the beginning of 2018 with my work coach Kathryn. She has helped me to build my confidence in looking for a job. She has attended job fairs with me. We have updated my CV and written cover letters. This programme has really built up my confidence and helped me find work. I am more confident in the workplace and happy. I feel happy and comfortable with the support I have received from Kathryn. |
| well the first day i cam i really wanted my cscs card for work and same day i came i got the course so i am really pleased and happy with the result i am doing it tomorrow lisa sorted my bus fair out i have a job interview when i finish the course i am buzzin thanks lisa i cant belive how quik she was i have not worked for 4 years and now im going to get of esa and get a job bbo syha all about you |
| I met lisa and emily when I was on esa and signed up to bbo. I had not worked for 3 years. they got me a flt licence and I am now in fulltime work as a stock controller. I am in a good place work and personally wise. they have both been excellent best thing I have done signing up to bbo |
| Accessing Building Better Opportunities has been helpful and taken loads off my mind in applying for jobs. Emily is very encouraging and supportive, she is a good listener. I would definitely recommend. |
| Because of a mental health condition I've been receiving ESA for a number of years. I do volunteer, but I've never stopped wanting to get back into employment! I've just needed to find suitable employment as staying in a job long-term has been hard for me. I joined the BBO programme earlier this year and both my Health and Wellbeing Coach Emily and my Work and Enterprise Coach Lisa have been amazing! They've been supportive, enthusiastic, have really listened to what I want to do and have given me the confidence to follow my dream of turning my hobby into self-employment. With their help I'm on the verge of my new venture and I can't wait! |
| I had a referral through Take Notice to Building Better Opportunities BBO. The two workers I see the business coach and the well-being facilitator have been remarkable they really are helping me to achieve my goals of becoming self employed which is amazing. Im actually getting there. They have been so encouraging and supportive all the way. I really have enjoyed working with them and will continue to develop. Sadly it’s just till Xmas but I have come on so much in the last few months. They have really invested in me and I feel well supported and valued because of it. BBO have had such a big impact on me and how I see my future it’s been of great benefit to have their support and progress my hobby to hopefully a business. I really appreciate all the help, the time, effort and resources they have invested in supporting me. |
| I remember it was a super windy day in Rotherham that I needed to hold tight my husband’s arm to keep equilibrium. I was searching for a job and he wanted a change of his own job. This is why we attended a job fair at Rotherham Stadium. With not many options to follow I decided to know more about a table that had a big banner with job opportunities for people with medical problems, either physical or mental problems. It was BBO (Building Better Opportunities) run by a quite big team at that job fair. I was assessed by a woman and she explained me about the project requirements and how I will be helped. To be honest, I didn’t understand much of it at that time. She said also I’m not a fit for the programme but she will do something for me instead, to help. She called Alex. “He will be your work coach.” she explained. “He will help you find a suitable job for you according to your skills.” she continued. I didn’t know what to expect and what to believe. I only knew that they will help me find a job and I need to attend meetings with them for this to happen. Well, I needed to meet Alex. Alex contacted me in about a week. We set up a meeting. We met first in Rotherham and he explained in detailed what will happen. I completed enrolment forms and presented my ID, my passport. I understood more about the project but not completely. It seemed unreal someone will help me search for a job. A job, which was not ordinary or randomly picked but it will be according to my skills, experience and personal needs. I was smiling at the end of our meeting and this filled my heart with hope. Since then, I and Alex met every week in a day we both agreed, excepting holidays. We started looking for certain positions I would agree and they were suitable to my skills. We build my CV and cover letters. We started applying. At first I wanted a part-time job and because I was dependent on bus, I couldn’t go further 5 -7 miles from my home, the options were not that many. Alex was always calm and never rushed things. He was understanding in any circumstances and wanted for me to get a good job that I will be happy to do it. I learned not only he will help to find me a job but will offer me support for the next 3 months in my new work place to ensure everything is going good for me and I will adapt in, properly. Each time we met, I think I began to be more open about me, my job to be and my working needs. I couldn’t think of any job, because I am a person who can’t do a stressful work for long. I couldn’t travel more than 5-7 miles from home because I didn’t have a car or driving licence and my commute with the bus or even train couldn’t be longer than one hour. A long commute and a stressful place to work would have jeopardised my health. I did this before and I had to quit too many jobs. I wanted this to work and I begun to trust more the project, and Alex. Before being part of BBO and meeting Alex, my work coach, I tried customer service roles, 5 times, it was 7 months I resisted the most of it, I was fired because of an absence, then warehouse, I couldn’t cope more than 2 weeks or even self-employed acting as Interpreter it didn’t work, there were too little appointments to interpret for and long distances to cover. I think meeting Alex and be part of BBO was my chance here in UK to find the job I was dreaming all this four years I had been here. Alex was very receptive to my words, my necessities, my story within work, and the experience, which I consider was little. But, above this I discovered my skills were the most important assets to get me a job I would do it without worrying I wasn’t able to keep it. Now I work as a Content Creator. The role is Bank Staff, which offers me a great flexibility, I can work as much time I can without worrying I will be dismissed. And, what is the best part is that I am doing creative work which suits me the most. I love it. Previously I had worked for one year as Graphic Designer in my home country. I was doing banners, flyers, business cards, all sort of promotional stuff. I learned that I enjoyed this job and I developed skills about creating all sort of advertising material. I dared to believe I will do the same work here, in Uk, that I will get a job in graphic design or at least a creative job. I felt was doing well my job at that time and wanted to continue, and even build a career out of it, after moving to Uk. But it was impossible to get that type of job for me without an English certificate/diploma and a portfolio I left in Romania, at my previous employer. Alex saw my potential and encouraged me to fulfil my dream, getting a creative job. It was pretty hard as I didn’t have any piece of my previous works. I could not prove my creativity and ability to work with a computer and software to build products, such as flyers, for example. He told every detail I needed to know about the job and asked to collect any evidence of my previous work to be shown to the managers. The job seemed perfect and I didn’t want to miss it. I offered to do a sample of my work instead of evidence of my previous job. He agreed and sent me text and images for the work I did at home. We strongly collaborated about this. The piece of the work he sent it to the right people and I received good feedback. Later, Alex set for me a meeting with a recruitment manager and we met all 3 of us. I did very well and later I was asked to attend another meeting to sign employment papers. We, Alex and I succeeded. He, as work coach was completely successful, he found me a job which was perfectly suitable with flexible working hours, and I could do it even by home or travel to the company. He also respected all my requirements about the job. And I secured a job which was my dream come true. I will do creative work and I know I am good doing it. I will do something I love. Just now, for me it is perfect. I hope someday will turn into a great career but this depends only on me, now. I am deeply grateful to Alex for believing in me and making my dream job come true. This was in October. Soon I learned of a great turnout happened in my life. I become ill. I was facing hard times. By that time I needed to meet Alex, sign the employment papers but I couldn’t do it anymore. One day Alex called me and I told him the difficult moments I was living. Alex was a great listener and assured me everything will be fine. We talked for more than an hour, he mostly listened to me. My words are not enough to thank for what he did for me that time. He also assured me the job I had now will be waiting for me. There was a two months break. I feared all our work will be for nothing. But Alex was right, my job waited for me and now I am working. I know am at the beginning but I feel that a great work adventure is waiting for me too. I am seriously happy. |
| Using Good Work run through South Yorkshire Housing Association. Had a work coach assigned to me through NHS due to help with my rehabilitation after a brain tumour was removed in October. Alex has help my confidence and motivation in seeking employment. He has also looked at my CV and approached potential employers, helping with applications and interview techniques. He has also helped with moving me towards a new career and I really appreciate everything he and the organisation has done so far. I believe with the help of Good work I will be in employment soon and if not for the Covid-19 situation things would have been sorted earlier but I’m currently in isolation due to the drugs I am on. |
| I have a wonderful care team with CERT (Community Enhancing Recovery Team) - NHS and SYHA (South Yorkshire Housing Association). I love living in the community and feel safe. I now live in my own flat and have recovered and have not been in hospital for ages. I've had lots of hands on support , I feel safe with SYHA support, with Matther my Keyworker, I would like to set up Tenants and Residents Association |
| I have been in and out of hospital for the past 12 years, then 2 years ago I got the support from the Cert team and Livingwell Project. South Yorkshire Housing Association who gave me my flat there support in helping me settle in to my property has been invaluable since being discharged from hospital. They have given me my independence and my life back, I feel as if I'm back now, and with their continuous support with maintaining my property and my mental health its going to be good. |
| My key worker, Beth, helps me to pay my bills I don't have the confidence to ring up to do this and I get really confused and stressed out if I do it on my own. &nbsp; It's really good to know I can call Beth when I need help. &nbsp; Since leaving hospital, I have managed to keep up with my payments which is a big relief. &nbsp; I feel reassured to know that Beth is there to help me with any issues in my flat. |
| What works well is going off the ward, the sessions, the one-one chats when we feel down. What's bad is the number of staff, staff not getting their breaks and there are no kitchen staff. The Managers and OT's are not attending community meetings. |
| Staff are good at listening and learning on what you say and staff are here to help and support you to get out of here. What could be better is staff could give us more opportunities and more things to do and give me or the ward more access. I would recommend St Andrew's because there's more freedom and it's better that prison or young offenders. |
| Don't have fun. There's hardly any staff, the ward gets unsafe which makes me feel worried. |
| I felt nervous but I felt safe. The staff were welcoming and nice. I like my room |
| I wish the rooms would be a little less secure. I know that they are made like that so we don't jump out but it would be nice to see outside, maybe through a vent. And in the common room to open up the window mesh so that we could see more. I just want to see what it's like outside. |
| Acorn ward is really good, it's more good than Bracken Ward I've heard. It's really noisy at night, although it's really calm sometimes here too. |
| I would recommend St Andrew's but what you could do better is change rooms, the duvets are made out of leather/plastic which makes you sweat.I can't use a razor even though I have never self-harmed but they allow me to use an iron. The food is a problem. The people that eat Halal have to eat microwave meals, that's the same for people that eat rice and stuff. The food is standard and it gets boring eating the same food every day.At night-time and in the morning I'm quiet but there is lots of banging but I think some staff are scared of the patients and asking them to stop banging. The patients that make a lot of noise should be on a different ward.If you miss a meal then you have missed it for the rest of the day - you are only offered a bit of fruit and some water.Otherwise it's good education wise. It's good at helping people and making them better and helping them to understand and stuff. |
| It's an alright hospital. The good parts are that you can do a lot of stuff like pool and football. |
| This hospital benefits you because there is a lot more freedom and a lot more to do than prison. You feel free as wellEducation wise you learn a lot. There could be more activities and taking people off the unit more. When I do go off the unit I walk around the grounds and get some fresh air, play basketball and football.There should be more staff. When there isn't enough staff people get silly, they can’t get off the unit and people can't do what they want or what they are looking forward to doing. I would recommend St Andrew's because there is more freedom. |
| When staff see that I am struggling they take me to the quiet room and try to talk to me. St Andrew's sometimes when they investigate things they take a long time and then they don't tell the patient what they want to hear or what they want to know about what is going on. There is not enough staff on the ward. We need more activities like going to theme parks, fun fairs and stuff like that. |
| Not much is good about the ward but sometimes it is quiet. English is a good session. I feel like we need more staff on the ward. We need more activities like more games and stuff to do and more outdoor stuff. The patient phone keeps breaking and the ward keep saying they will change it but they don't. I would recommend St Andrew's but I feel that all they need to do is get more regular staff. |
| It's boring just sitting on the ward. With all the doors being locked it's frustrating not having your freedom. We could do with more sessions like football. The staff look after you and the food is good. It's a good hospital to come to for some people. |
| I would like monkey bars in the courtyard. The ward is brilliant and the staff are helpful. The things we do, like going out, are good.This is the best hospital I have been in and the patient’s are very caring. |
| The food can be disgusting at times because sometimes it's not edible because sometimes it's hard and sometimes you can find hair in your food but we haven't seen that for ages.My bedroom is alright. I don't think I've got any problems with my bedroom, it's my safe place basically. One of the shifts - they left me to head bang and I sat crying. They leave my constant observations all the time. On the other shift the staff help me. If they think that you're a risk to yourself they strip your room but they put it away safe, like in my bathroom. The ward could do with redecorating, like painting the walls and ceiling. We could have a football goal painted on the wall in the courtyard, although we do have a basket net. I think we should be able to paint our bedroom walls any colour although we can have our own posters on the wall anyway. We could do with a patient coat hanger in the corridor.I would recommend St Andrew's because they have helped me through my bad times. When I first came I was a danger to myself and others but now I’m getting better. If someone was poorly at home I would recommend it to them. |
| Animals Music Ice-Cream Good to get off the ward |
| have been at St Andrew's already a year, I moved from JCU to Berry ward, There are more things to do on Berry Ward, I use the art room a lot - they have a separate art room which helps me with my A Level art. staffing has improved since being at Fitzroy. However there is a lot of new staff. I have learned more coping skills since being here. I would like to be able to get out of hospital. It is important to have staff that are permanent rather than agency staff. Staffing during in the day are more regular, staff during in the night are more difficult to get hold of. We don't really get to know the night staff. |
| There are lots of different support networks in St Andrew's and the nursing team work well together. We don't have enough staff on the ward (mainly nights), which means we can't always go out. |
| The ward is quite settled and friendly. The structure is good and it's planned so you know what you've got each week. If you are unwell it's a good hospital to be in. |
| I would like the ward to help me get better things that I like. I would like help to get a new jacket. We play games like scrabble, football, pool and sometimes I go walking around the building but now I've got leave and I can go gardening at Hawkins Garden. I like woodwork and I go to light industry to do this twice a week. I do art work because I like doing art. Sometimes I sit with other service users and play games with them, we go upstairs sometimes and play football in groups of 5 or 6 aside. The other thing that is good is I see my family once a month. I don't like it here because sometimes we get treated like kids - shouted at. It's only some staff I like here that are kind. I get jealous of people eating lots of sweets and snacks and I wish that once a week I could have more than I do but I have type 2 diabetes. |
| They are doing my treatment. It's really good treatment because I help them understand what medication I take. I attend every session, some of them have helped me get a silver Koestler award. There are lots of activities happening on the ward like baking, making masks, movie quizzes and film nights. We could do with some more staffing because staffing seems to be a problem, and we need an extra cleaner. I would recommend St Andrew's if your needs had to be met. The sessions I do are DBT four times a week, light and heavy industry twice a week, animation once a week, music once a week, working at the church twice a week and physio once a week. |
| When you are in the moments when you feel like giving up, St Andrew's provide you with guidance and advice. When things look bleak there are good opportunities such as leave. The ward should learn to do Schema Therapy because when you have completed DBT then Schema Therapy will help you use your skills in times of crisis. St Andrew's is recommended for all different areas of Mental Health. |
| The patient Summer Party is brilliant. I've met all the people and done all the activities. I had a burger and ice-cream, I had my face painted and I got a glitter tattoo. My favourite thing was the ball game. |
| t's really nice here. The staff are very caring, they make sure that they support all your needs. There is a lot of facilities like football, gym and snooker and you can have community access which is good. Staff are there for you when you need them. I wish that they brought smoking back around the site. I would recommend St Andrew's because it's a nice hospital, its one of the nicest hospitals I've been in. My last hospital wasn't very good but this one is. |
| I think it's a really good hospital and I don't like all of the bad publicity at the moment. There is a lot of good things that happen that people don't hear about. Staff are like a family and they wouldn't let anything bad happen to us. I am blind and I have got so much more confidence since getting here. |
| They are always short of staff! I missed trip out to Workbridge as a result of no staffing. |
| the ward today was too short of staff, there were no staff to take me to the computers and no staff to help me when I ask for it. it made me feel s*** and not wanted. |
| Due to staff shortages in Birmingham, they have cut our leave for the past three weeks. Therefore I cannot go on my usual community leave to go shopping. My community leave is now restricted to once a week. They have put other leave on like ‘walking groups’ but this is not the same as my individual leave entitlement. How can they be integrating us back into society, and be cutting back on our leave? |
| I was sent here as my last hospital couldn't handle my challenging behaviour, I had a 6 month stay in hospital in 2013, I've been stable up until the last few months. The staff here are very helpful especially the Doctors, they are listening to what I say and think about how I should be treated, and I listen to them. (it reminds me of the film 'One Flew Over the Cuckoo's nest' ) |
| I was sent here from somewhere else, they are very caring and compassionate, they have helped me on many occasions when I needed them, they have saved me once or twice, they are always there when I need them. they could change their attitude a little bit and the way they approach people. |
| Well done to all the staff who really got into the spirit of Halloween. The whole place was decorated and they masked up with face paints and halloween garb! It makes me happy for Alistair (our brother) and us to be part of the Rix House family.It was really good that staff let service users join in with halloween |
| I'd just like to say what a fantastic night was had by all. Thanks to all the staff who must have worked really hard beforehand to put on such a fabulous night. The turn out was brilliant and there was something for everyone, pass the parcel for the residents and one for the children too, not to mention the raffle (we did win the sweet hamper, yummy!) And of course Sue's bingo, she did have a couple of helpers giving her a hand and I must say she better watch out! None of this could happen without the hard work and dedication that the staff put in, they seriously go above and beyond, I couldn't wish for my brother to be in better hands. A big thank you to all of you |
| My daughter has been in several nursing homes over the years, this is by far the best. I have complete peace of mind that my daughter's needs are well taken care of and that she is happy. The staff always keep me informed about my daughter, if she had a fall and what they have done to make sure she is alright, any activities she has taken part in. |
| The nurses from the team were all so lovely- Abby and Becca particularly in Mum’s intense last few days were so kind and caring. Always felt we could depend on one of the team to support us and with telephone advice from St Christopher’s they managed end of life meds/ syringe pump admirably. Thank you to a wonderful team. |
| Mum was discharged for community end of life care under St Christopher’s but with daily care support under NHS continuing care from Bridges Healthcare who were wonderful - Sue came to meet us in the hospital and was incredibly kind, considerate and caring at a difficult time. All the carers who came were respectful and truly caring- always addressing Mum directly and being sensitive to her needs and symptoms. Whenever there were staffing difficulties (completely to be expected at the start of the Coronavirus outbreak) a manager would step in and come to provide care. Very grateful to the wonderful team from Bridges. |
| I could not thank the Advocacy Health Care profession any more for supporting me on my journey to recovery. I was at Talygarn Ward, Pontypool, under Daniel, he had supported me throughout months on the Ward, having his presence alone at my wards rounds was helpful and supportive, he took all my needs into consideration and asked questions to the Doctors and nurses on my behalf. He waited with me for hours of delayed timings and never moaned once, Very happy positive person with excellent knowledge of Mental Health Law that I did not know. Daniel deserves a promotion! ! ! |
| My experiences is that of the meeting with advocate Theresa. Apart from daughter who due to cost and travel distance visits were limited. However Theresa was a regular visitor to me. She spent time with me in which we engaged many and varied topics of conversation. Theresa always maintained an interest in my well being and wherever possible carried out my wishes and requests. Some times personally. Theresa prepared her meeting case notes meticulously. |
| All of the other professionals talk to me and I don't understand what they are saying but Gareth makes it easier to understand. Tank You |
| Great support from Pete (advocate) at caswell clinic. |
| The Advocacy Support Service has been of immense help to me and my family. The information we have been given about the law and our rights have been very good as they were things we did not know. Antonia Woods has helped me and been by my side to support me. She is very professional in her approach but at the same time very caring and knowledgeable and supportive. She listens to my views and is always there if I need to ask her anything. The service is excellent and even the people on the switchboard has been helpful. Nothing could of been better, The help we have received has been over and beyond anything we could of hoped for and will help me have a better future due to this. It has made me feel valued and treated as a individual, also empowered. It has also helped relieve the pressure on my family in trying to get help that we so badly needed. Thank you for all your help |
| My advocate was very supportive. She informed me of my legal rights and supported me during a meeting. after this meeting I was discharged from my section and I was able to return home. Having her in the meeting with me made my consultant listen to my opinon. |
| My advocate visited me at Princess of wales Hospital. She informed me of my legal rights and gave me the confidence to challenge my doctor.. I am very grateful for Caroline's support and services as it has enabled me to become more involved in my care and treatment. |
| thank you for your support. There is one person in particular who I feel has exceeded every expectation in my eyes. Hannah. Your colleague has gone above and beyond her job role after the community mental health have failed to provide me with a fair service. She has been a complete professional within her job role. No request is any hassle to her. And is always carried out timely, and always with a smile. Hannahs job role is to advocate for me. To be my voice. And I can get 100 percent she has given me the confidence to speak up as I feel like I'm not alone. She has also been a good listener. Which is not within her job role. I have been unwell recently and felt that no one would listen. After speaking to Hannah and talking things through I felt that I was able to carry on. I just wanted to say how lucky you are to have this young lady within your organisation. I never write letters of compliments because I'm usually not impressed. I cannot believe the level of good service she has offered. She is a hard worker, someone who can use process effectively but is able to see the human element of things. I cannot thank you enough Hannah for all your hard work so far. At this time you are giving me and my family the solid foundation for us to have a voice. And for me to speak up and be heard |
| The first time I was lucky enough to meet Rikki was in 2014 at the start of the summer. I was a little nervous probably because of the title of the job she held. So I asked her to explain what exactly the role of an advocate was and in fairness Rikki explained slowly and clearly and when there was a couple of points I wasn't clear on she changed the style of the explanations and I knew what was going to be done by Rikki for me. Which again was a breath of fresh air as when we would next meet up, not only was she on time but the information I had requested was neatly put together and [photo copied. If anything wasn't clear, Rikki would spend extra time explaining the layout and what would be the best method of approach. Up until present time and day I can quite 100% honestly say Rikki has been a massive asset to my confidence in dealing with my case issues and figure of loyalty helping me invaluably to give me the confidence, to apply this is dealing with the mental health system. I personally would recommend anyone of either gender to request her exceptional professionalism. |
| I'm very pleased with Rikki's help. Excellent member of the advocacy team. I feel happy and satisfied with all the help and support. Nothing to improve, all ok. |
| Hannah P. of the Mental health advocacy Support Cymru helped me so much to prepare for tribunal. She even asked me how my illness affects me. She wrote a letter explaining my problems. So it could be given to tribunal people in case I couldn't speak. I'm so grateful. It would be good if advocates could accompany clients or patients to tribunal. Especially as some people find it hard to speak. Thank you so much. My money is going to be reinstated. |
| Peter is doing a very good job. He has approached every situation well. I am more than happy with using your service. |
| I feel that Paul, my advocate is very good because he listens to me & helps me understand what is happening especially if there are any changes. He makes me feel comfortable & less worried about things. I am happy with the way the service is at the moment & wouldn't change it. |
| Debbie the Independent Mental Health Advocate and I had a wonderful conversation. A lovely lady. . . We discussed at some length, the policies and procedures. She came to see my doctor. She played an excellent role in advocating and asked pertinent questions. |
| I felt at first no one understood what I was going through. Also felt I was being pushed into decisions that I had no control over. But after Dan came into the ward rounds with me I felt a lot calmer and in control. The advocacy is not communicated enough on the ward as a patient's choice. |
| My experience with Gareth was a massive help with me getting my words across. And a massive thank you to him and the whole team. |
| Whilst in hospital, I was supported by Deborah. I am very much into female empowerment. That's what midwives do. All of the advocates have been fantastic. Dafydd has also been extremely helpful. Thank you |
| Gareth is very caring and supportive, I found the service helpful. |
| I have had a very rewarding and positive experience of Rachael, my community advocate. I was facing several medical assessments -for benefits PIP + ESA, as well as for specialist NHS treatment. My health condition makes consistent cognition difficult + I become exhausted very easily. My memory is also at times poor. Having someone to come with me, whilst I discussed very personal matters was crucial. It would have been inappropriate to take a friend or relative in my case. She has been informed and understanding about my condition, as well as the meetings I have needed to attend. She has also supported me during my recent redundancy + subsequent TUPE + redundancy. She has been utterly dependably, available + has produced typed notes after every meeting which has been invaluable. She has also listened accurately to my needs + produced very professional letters + emails to various figures of authority. She has enabled me to challenge mistakes + continue to support my family through a difficult time. |
| Burhan was there for me; he was a rock when all others could only express concern at my past behaviour. I was on a Section 2. I knew I could take steps to get better. However, it was Burhan who gave me hope, and helped make me feel like I had someone in my corner. He was able to arrange 2 hours unescorted release, which allowed me to visit Caerleon Church and the Roman Museum, and even get a haircut. I was able to stretch my legs, but more importantly feel like there was hope, at a time when I was down. Burhan was there for me at my lowest point. He helped my recovery. I owe him a huge debt, that I can never repay. It was Burhan who helped make me feel like I was worth fighting for. Thank you Burhan. |
| Rachel has treated me very well and has been excellent in dealing with me. I'm only 22. Yes she is the best for me, I think she deserves a special present and, I will get her something special. She has been the best to deal with me, very smart and knows her job. My daughter and I are very grateful for what this lady has done for me. I think, she the best I have ever dealt with, I will always remember what you have done for me and my my daughter. I also would like to speak to her Boss to say something nice to her boss. xxxxx good luck never forget you |
| I had been in a mental ward for several weeks and was not doing so well at the ward round meetings until I met my Advocate named Dan, He helped me by discussing my rights under voluntary status and the laws of mental health, I talked with him about my history and care and how I didnt need to be in hospital any longer and could be treated in the community. I never felt like I was being judged for my mental health and was able to talk to him openly about my current situation. Dan helped me organise my thoughts and wrote down notes to help me structure my points and goals for what I wanted to happen in the ward round meetings, He also let me knew what rights I had as a voluntary patient such as being allowed out on my own on unescorted leave, Dan also ensured that when he was on leave during one week, That I wouldnt be without an advocate or support and passed my notes along to his colleague named Sharon who came to the meeting for that week, The notes Dan wrote helped her get up to speed quickly and she was able to help me greatly in that meeting as well. I was so glad in that final ward round when I got discharged, I shook Dan’s hand afterwards and thanked him for his help, Im back out in the community now and I am very grateful for the advocacy support. |
| My Advocate (Dafydd) helped me whilst I was in hospital, he is brilliant, Dafydd was so good and caring. I found him more helpful than my solicitor. Dafydd always listened to me. |
| Dafydd was really knowledgeable + helpful throughout my experience. Thank you so much for all your help. |
| I was in St Cadocs Hospital and need an advocate. &nbsp; I used the advocacy service. &nbsp; Hailey immediately put me at ease. &nbsp; I felt very comfortable talking to her and what I said really mattered. &nbsp; She offered supported at the ward rounds to assist me if and when needed for which I was very grateful. &nbsp; I felt almost like I had a friend who believed in me and was there for me. &nbsp; Hailey unwaveringly continued to offer support at any level. Anything she didn't know she found out and came back with the information. &nbsp; Nothing was too much trouble. &nbsp; Hailey is a warm kind person and was genuine in her care for me. &nbsp; Excellent service - keep up the good work - you really do make a difference |
| I like the advocacy team because they are friendly supportive + caring and funny at times and make you feel less nervous in meetings. Debbie is my favourite so I must mention her. Thats my short story. (Ex Patient Oak Ward Hafan y coed) |
| 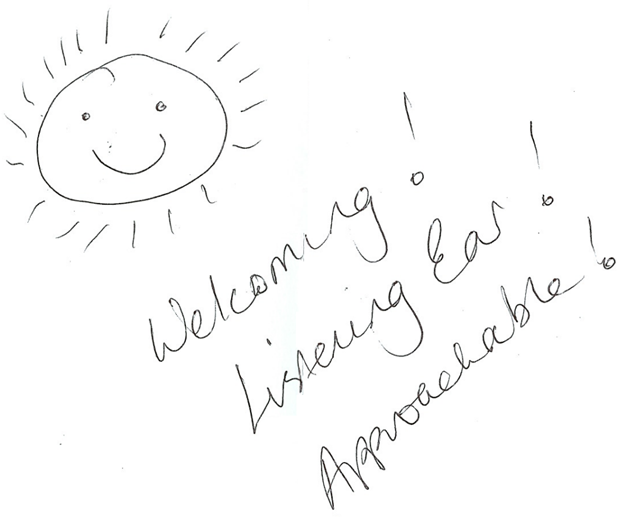   \|  \| \| --- \| |
|  |
|  |
|  |
| I accessed Advocacy Support Cymru and dealt with Mr May. I found the service to be excellent and could not find fault with the way Mr May dealt with my case. I think that Advocacy support provide an valuable service for patients in mental health establishments in Wales. Mr May was very approachable and helped me work through a difficult issue with my care team, I would have found it very difficult to get myself heard without Mr Mays assistance. In conclusion I would not hesitate to use Mr May again and can only thank him from the bottom of my heart for his assistance and satisfactory outcome. |
| Hannah has been very supportive to my son and to myself as a parent. She has put educational plans in place and attended meetings to ensure my son is not disadvantaged and to make sure his educational support is met. It has been a year of battling and there has been hard times but we have got there in the end. My son is doing very well, his self esteem has improved, he is a lot happier and he has reached top of his class in maths, and is enjoying his new apprenticeship. Thanks Hannah for all your hard work, without you we would still be battling. |
| I'm a single lady that has been diagnosed with PTSD's and Boarderline personality disorder. The support I received and still am from Rhiannon has helped me so much it's unbelievable. The mental health service let me down, so I contacted Mind. I was allocated Rhiannon and A complaint was made on my behalf and has now be resolved. Without Rhiannon I do not know what would of happened to me and where I would be now..I cannot thank her enough! |
| My advocate matthew fought for me in a meeting with my care team. I was to tense and overwhelmed to speak. It was good to know I wasnt alone. It felt like my team didnt believe me but he did and I think he got on their nerves but they listened! |
| Lucy, I would just like to inform you that we have received the letter from the concerns team and just to thank you for all the help and guidance you have been providing for my mother. Things are finally starting to look positive 😊 |
| Rachael has done a fantastic job, really professional and I couldn’t ask for more. |
| As a Ward Manager, I would like to say many thanks to Hayley for being the voice and support for all our patients during their stay on the ward. |
| Thank you Sophia for what you have done for my sister. You have helped a great deal. Things are working out for her now although they are taking things on a day-by-day basis. |
| Just wanted to say to Deborah ‘thank you’ for being my advocate during my admission, and for all your support, it is very much appreciated. I hope that you are able to continue to be my advocate now that I am at home. My very good wishes. |
| I feel very supported, by my advocate, Gareth! I appreciate the support of having a _voice_ here at Tŷ Llidiard |
| I want to say thank you from the bottom of my heart to my Community Advocate, Rachael. I know the outcome I have achieved wouldn't be happening if it wasn't for advocacy. The job you do really matters and makes a difference in peoples lives. Thank you. |
| You have cared for me so good, thank you so much for all your help to sort things out for me, you have been outstanding in every way!!! Kind regards |
| (Howell Ward) You're services has been "A one" Bernado has provided the support I needed. I would recommend him to all service users here. Thank you. |
| My advocate is an excellent advocate & accompanied me on two ward rounds. They explained the technicalities and my rights as a voluntary in-patient. During my stay at Tal-y-garn hospital I have been - • Denied access to a bible or any religious or spiritual book; • Denied access to a prayer room; • Denied the right to walk in the grounds, even after the 72 hour monitoring period; • Been ignored by staff when I requested help following a seizure/fit due to acute stress reaction under instruction from senior staff My advocate has helped me by signposting me to the community health council & healthcare inspectorate Wales to make a complaint, and helped me to request input from psychology and social work professionals. N.B. • Prior to admission I had been homeless and slept rough and in the car. • I was already receiving help via home treatment team. • Admitted voluntarily at the behest of my psychiatrist. • Only therapy I received other than drugs was 15 min relaxation in 5 days on the ward. |
| I have had good advocates in the past and Dafydd is up there with the best of them. (He is) friendly, professional and a pleasure to be around. |
| I am writing in relation to your colleague, Ms Sweet. Over the past months Ms Sweet has been assigned to assist and guide my wife during a very stressful and demoralising period dealing with the CMHT. During the early stages of the fact finding meetings with Ms Sweet we both found her to be very thorough with an skilful ability to make sense and document a free flowing conversation with my wife with total professionalism and sensitivity. Ms Sweet always made my wife feel at ease and reassured her that she was available to help guild with any up coming CMHT meetings. In order to encapsulate the totally professional and simultaneous empathetic approach that Ms Sweet gave to my wife's case is shown in the outcome of the long awaited appointment with Dr M in which unfortunately due to work commitments Ms Sweet was unable to attend, but following a meeting with my wife we compiled a discussion check list which gave Louise the guidance and confidence to attend the appointment with Dr M on her own . Following the successful appointment my wife commented that she was only able to see the appointment through because using the bullet points compiled with Ms Sweet "it felt as if she was there with me ". So to close I would like to thank Ms Sweet for her knowledgeable and professional care throughout these testing times and also a appreciation that the Advocacy Support Cymru are there to help which we have no doubt without such an organisation the outcome would have been a lot less satisfactory. |
| Rhiannon, Thanks so much for drafting that letter! It's fab and realy thorough! Its lovely seing it actually, to feel like someone listened! |
| Thank you for all your help Hannah, much appreciated. Look forward to meeting my new advocate but your hard work hasn't been forgotten. |
| Lucy - absolutely fantastic, I am very impressed THANK YOU SO MUCH, nobody has ever done anything like this for me in my life so I have just got used to being and dismissed ignored so thank you so much for everything you've done. |
| Dan, my IMHA has been an outstanding support for me and my family. At every ward round he come and supported me and helped me understand all the processes involved. I also had to make a complaint about an assault against me - his help for me was so critical and empathetic. As well as being knowledgeable and supportive he also has a warm personality and makes you feel as your problems really matter |
| Thank you Mat for all your support and encouragement. Unfortunately the consultant refused to meet with me today, and with support from you and the ward nurses on C ward I decided to leave with my parents. I found the advocacy service really helpful and reassuring and am just really sorry that it wasted time for you when you stayed around on C ward on the understanding we would be seeing the consultant this afternoon. I am going to be getting support from family, friends and the crisis home treatment team. Thanks, |
| I was transferred to St Albans Nursing Home this morning and want to thank you Deborah for all your help. |
| Thanks for yesterday Rhiannon - you really helped and were great in the meeting. I spoke in depth with my therapist today about the meeting and how although I found it very difficult I was able to stay relatively calm and we spoke about how that was largely due to your presence and so thankyou so much. |
| Very helpful indeed and listen to what I and others say because after all everyone has a voice. So thank you very much Rikki for taking your time out to talk with me. So thank you |
| Matt is lovely and really good at his job, and so is everyone because you are independent! |
| I just wanted to let the community advocate know that I have met with the doctor and it actually went really well. He confirmed my diagnosis and things are now looking better in terms of getting a referral for DBT. I just wanted you to know because you helped so much and I really want to thank you for helping me because I would never have met with the doctor if it wasn't for my complaint and the meeting that followed on from it. Thank you so so very much for everything! |
| I really appreciate all your work, thank you |
| I was admitted to Talygarn Psychiatric Unit, Pontypool, South Wales. When I first arrived I was anxious and worried about having my ward round with my Psychiatrist and mental health team. Initially it was a daunting prospect and I felt alone in getting my voice heard and expressing my opinions and concerns during ward round as well as pushing for the treatment that I wanted and believed was best for me. After meeting with Dan, the IMHA, the concerns I had about my ward rounds faded, I felt confident that my views and decisions regarding my treatment would be heard and respected. I can honestly say Dan is the best IMHA I've had, he is extremely knowledgeable in every aspect of mental health, admission on the ward and my care and treatment. Dan has been a consistent source of support and reassurance, he has always made time for me, even with his very busy schedule and work across different wards. From day one, he always fought my corner, always being there when he said he would even when he was sick and should have been in bed recovering, he never let me down. I truly think that if every advocate displayed the same passion and pride in their work and courage of their convictions that Dan does on a daily basis , never faltering or wavering under pressure, then every patient would have the level of support and information required to understand and exercise their rights and have their voice heard. |
| Chris is a fantastic advocate (IMCA) and has really improved my life! |
| I am very grateful for the time and effort Dafydd put in when he was at Hafan y Coed last year. He supported me with issues for court. |
| I've been sectioned for the last 3 years and am soon to be discharged. It's been a long road and I've gone through numerous advocates but my current advocate, Susan, has been one of the best advocates I've ever met. She's been patient and understanding with me and has always fought for me to get what I need. I am so grateful and I would not have been able to go through all the meetings without her. Because I have autism, meetings are incredibly stressful and I can't speak in them at all. But Su was patient and went through everything slowly with me so she knew exactly what I needed her to say on my behalf. I will never forget the amazing contribution she has made to my life and I am so thankful! THANK YOU, SU!!! |
| Thank you Lucy for your continued efforts and support since you have been in post, I have to say its lovely working with someone so keen and committed |
| Thank you very much Rachel for everything that you did to ensure that the best interest meeting went ahead yesterday and for your contribution to the meeting, I really appreciate the effort that you put in. |
| If it was not for Shelly's support at ward rounds, fighting my corner and challenging the doctor and staff, I would have been stuck in hospital forever, she helped get me discharged and moved on to my preferred placement after being detained in hospital for so long and being threatened with being moved to a low secure unit. She is a star. |
| Dan is very good he always gets things done |
| would like to very much thank Burhan for the advocacy support that I have received whilst a patient in hospital. |
| I have never felt supported in all the time I have been detained until I had IMHA support. Shelly was very knowledgeable and gave me a voice, she challenged the consultants who I felt were often fobbing me off, pinning the doctor down to concrete answers and helped with my discharge from TCR after being detained for so long. |
| Before my advocate started to attend my ward rounds, I had no rights or voice and believed I had to do what my consultant said. My advocate made sure I knew my rights, elicited my wishes and feelings, and challenged my consultant about the rationale behind the decision to move me. I am now being discharged home and am very happy. I would not have got this result without IMHA support. Thank you. |
| Since my IMHA started attending ward rounds, explaining my rights and challenging decisions, I understand what is happening in relation to my treatment and care and can input into decision making. I now feel listened to and my wife agrees that we were getting nowhere without Shelly's support. Thanks for the help |
| Thank you for supporting me in my meeting yesterday Rikki. I'm really pleased that my request for a scan was taken seriously and agreed to by the doctor. Thanks for working hard on my behalf. God bless you! |
| Thanks for picking up the case at the last minute (after it had become clear family not appropriate) and the swift response - much appreciated |
| A huge thanks you to Hannah for helping me through this stressful time. You have been a wonderful help and I'm so glad I met you. I wish you all the best for a bright and successful future. |
| Cal was very helpful. |
| I would like to thank Rikki for a detailed and thorough report submitted for a 39A referral, which outlined relevant points of concern. We will take apt actions to alleviate these concerns. |
| Hi Mathew, thank you so much for all your support. You have a great approach to the work you do - personable, calm and knowledgeable. I would recommend the service to anyone. Even the staff on reception are patient and friendly! |
| Good work Roisin, you should have a raise and a Christmas bonus and a company car and free petrol… Just sayin' |
| Lucy did a really good job for me and helped me, can you give Lucy a pay rise for me, she worked really hard for me. They have now moved me, and I would like you to thank her for me, she deserves a pay rise |
| Mr Lerwell was very efficient. He was very helpful and he provided his report in a timely manner. |
| I was a patient on an acute ward in Royal Glamorgan hospital, I had issues around rights, leave, medication and discharge, which Shelly supported me with by explaining my rights, speaking with ward staff and social worker, challenging decisions, and helping me prepare for ward rounds. I've been discharged now and want to thank Shelly for all of her support. Being in hospital has been a very difficult and lonely time and knowing I could call Shelly for information has made a big difference to my time in hospital. |
| Thank you Shelly for the work you have done with a family member in relation to discharge planning. She did not want to engage with the care team until you became involved. You put her wishes and feelings forward, which enabled her and our family to achieve the result we wanted. Having challenged the decision about discharge, she went home with a full package of care. As family members, we would like to thank you as we would not have achieved this result if you had not been at the meeting, you ensured that the least restrictive option was considered. Her husband says that no one had been listening to his wife’s wishes until you were involved. |
| It was very good to have the advocacy support from Dan Richards at Advocacy Support Cymru..... he was very kind and there for me 100%. Dan was even on a conference call when it was my ward round and was giving me some advice when I needed it. Good to know that I had someone on my side. Kind regards. |
| Hello Julie I just wanted to feed back to you once again how thankful AE was with all your support during her complex discharge planning, as you’re aware she required an advocate as she felt her family were not supporting her with her wishes and feelings. A was extremely thankful for all your time and effort in keeping in touch via phone call e-mail & skype during these difficult times, she has consistently told me she had full trust in you as an advocate and felt you were “fighting her corner”. As a DLN I would also like to thank you for all of the support you have provided patients on my caseload. By the very nature of my role and referrals I receive patients can be extremely complex with very difficult family dynamics working alongside you is a great support to both myself and the patients you advocate for. |
| I recieved a very thorough and helpful report. |
| Hi Cal, Thank you for your encouraging words and support. |
| The nurses from the team were all so lovely- Abby and Becca particularly &nbsp; in Mum’s intense last few days were so kind and caring. Always felt we could depend on one of the team to support us and with telephone advice from St Christopher’s they managed end of life meds/ syringe pump admirably. Thank you to a wonderful team. |
| My second youngest daughter goes to Riverside spc. She has a diagnosis of ASD and Adhd and is on medication for her adhd. Which affects her sleeping pattern quite frequently which often leads to late late nights and early mornings especially on school days. She has three other siblings an older brother a older sister and a younger sister who also has ASD. She started going hollybank three months ago and settled really well. She often comes back full of beans and very bubbly. The staff I’ve only met once at a coffee morning but have spoken on the phone and emailed communication is excellent and I don’t know what I’d do without them. I’m very impressed and very appreciative of the staff Thank you. |
| My two autistic children went to have a sleepover at Hollybank. My social worker made provision for my two children to have a sleepover to enable myself and husband as a time on our own. I was anxious initially , as my two children has never gone away from us but the times we had with the staff at hollybank and looking at their professionalism and working gave us some confidence to give it ago. The staff gathered enough information about my two children. They had a great time there and we (parents) were so happy to have taken the opportunity in helping them to be independent Thumbs up to all the staff for making my children welcome and happy in their first night away from home. Thank you very much. We look forward for more time with Hollybank team. |
| My son has been going to Hollybank for some time now, i can say in the first few months i was nervous as there were lots of negative care home stories in the news at the time around people with disability being mistreated. I can now say i have confidence in Hollybank that my son is well cared for , i no longer worry -sometimes i even forget to call and check in-its like he is with family. Staff are brilliant-involves family in care and communicate well with me. It is a great service and was a lifeline when my son was at his worst- appreciated this support soo much. |
| My daughter has been attending Hazeldene community based services for people with learning disabilities where she has been part of "Lunches to go", catering for events within the council as well as providing lunches in the canteen for service users and staff. Whilst being part of a catering team in a working kitchen my daughter has learned a wide range of skills and knowledge of health, safety and hygeine, successfully achieiving her rehis food hygiene certificate last summer. Perhaps the biggest change has been the increase in confidence and self esteem. She really felt part of the lunches to go team and there was a great camaraderie between both staff and service users. With her skills, knowlege and confidence my daughter has been able to move on into her first paid job as a catering assistant in our local primary school. Although its early days she is doing really well and for her to have a paid job within our local community is a wonderful achievement. Many thanks to all at lunches to go and Hazeldene who have been part of my daughter's journey so far, without you she would not have been ready to take this next step. I know it is a time of change for the service, with Hazeldene closing and the new Trindlemoss due to open in May. At the end of last year there seemed to be a lot of uncertainty about what the new service would look like. I hope it will include the lunches to go team and that service users will continue to have the opportunity to learn and develop as my daughter has. My own opinion for what it's worth, while we have been very proactive in supporting my daughter into employment, from applying for the job, the interview and supporting her over the next few weeks while she finds her feet, not all families are willing or able to provide this level of support. In this time of austerity and cuts in many services the council needs to look carefully how they target their resources if they are serious about people with learning disabilities moving on into employment opportunities, whether paid or not. Targeting training to employment opportunities and providing job coaches and support to ensure a smooth transition is crucial. I also believe the council itself needs to take a lead in providing and promoting opportunities within it's own organisation. I will watch the opening of Trindlemoss with interest and look forward to seeing how the service develops and I hope there will be many more stories such as my daughters.  . |
| My daughter moved from Hazeldene to her new workplace Irvine with Lunch to go the team has built her self esteem and improved her skills. My daughter has grown in confidence and I cannot thank the lunch to go team enough. I would like you to pass my appreciation to the team for a job well done. |
| As a parent of an adult son with severe learning disabilities, autism and adhd I was persuaded to apply for tenancy for him with 24 hour. carers social worker has not been available for the last 4 months. I phoned dept to see where progress was going. I got no reply. Son offered a property in Dec so called social work again. To be told have to wait until April for care package to be put in place. so either reject offer of property or care for him myself until April. Not good enough. Disappointed in how the dept have ignored me and family |
