# Supplementary material for: Systemic safety inequities for people with learning disabilities: a qualitative integrative analysis of the experiences of English health and social care for people with learning disabilities, their families and carers
Source: Int J Equity Health. 2022 Jan 28;21:13. doi: 10.1186/s12939-021-01612-1 (PMC8795982; doi:10.1186/s12939-021-01612-1)
Supplement: Supplementary file 2 — Additional file 2. [file 12939_2021_1612_MOESM2_ESM.doc]

**Focus Group 1**

**Interviewer –** So, if we think about a time when you’ve been to see a doctor, or a nurse or you’ve been to the hospital, what sorts of… things come into your head when you think about going to see these people?

**P1 (male assistant) –** I’ve never known them so quiet.

**Interviewer –** Are you happy, are you sad?

**P2 (female assistant) –** [name], what about you?

**P3 (female)–** Can’t think of owt.

**P2 (female) –** Can’t think of owt.

**P3 (female) –** I like to be honest about that

**Interviewer –** At what sort of places do you see doctors and nurses?

**P4 (male) –** At the hospital.

**P2 (female ) –** At the hospital.

**P7 –** Medicals.

**Interviewer –** At the medical centre.

**P7 –** Yeah.

**Interviewer –** Yeah… And what’s it like for you when you go to the hospital or go to the medical centre?

**P7 (young boy) –** Not good. Not, not good for me.

**Interviewer –** Not good for you. In what way is it not good?

**P7 –** Because…when you make an appointment to see a doctor or nurse or a doctor’s appointment you’re on the waiting, uh, waiting for hours and you’re just waiting for hours and you like sit there and they don’t call your name and they go well you’ve missed your appointment or and it’s like well, it’s not my fault I’ve just been running a bit late for something. Just like, they don’t seem it, when they make appointment, it’s like, that’s upsets me because it’s more important for me to see the doctor or nurse. But… like when I went to see the nurse for some blood…

**Interviewer –** Yeah.

**P7 -** …they took my, like, I had to go back again the next day and it were just a waste of time… and it annoyed me mum.

**Interviewer –** Yeah.

**P7 –** Cos me mum made that appointment and they sent it from the hospital…

**Interviewer –** Yeah.

**P7 –** To make that appointment and they didn’t seem it. And I had to waste, I had to get up at half past nine to go there and drive there and then come back and the next day I had to come back.

**Interviewer –** And do it again.

**P7 –** It’s a waste of time.

**Interviewer –** Yeah.

**P7 –** I’m sorry but it’s just a waste of time for me to go to doctors. For my mum, cos my mum gets annoyed because she just gets upset because I’m not seeing the doctors all the time.

**Interviewer –** And so… can you think of a time when you’ve been to the doctor when it was a good experience, what was different about that?

**P7 –** Uh… they helped me… they helped with my, like, get, they helped me more like with my medication or with helping see a doctor. Some doctors are okay and some doctors are not. The doctors that I see are fine and it’s perfect. I get on well with them when I see them and it’s good, they give me good advice.

**P1 (male assistant) –** Did they explain…

**P7 –** They explain what…

**P1 –** In a simple, in a simple format to you.

**P7 –** Yeah.

**P1 –** Urm, like when you were having your blood test.

**P7 –** When they took…when they…

**P1 –** Did they explain, did they explain what they was going to do.

**P7 –**Yeah they said inject me and then, and then I said what are you __(inaudible 4:05-4:10)__ they explained it.

**P1 –** And they didn’t use any long, jargon words?

**P7 –** What do you mean?

**P1 –** They didn’t use, say any words you didn’t understand?

**P7 –** I, uh, no no I, I asked if, I said could you repeat it again and they said they would repeat it again.

**P1 –** Okay.

**P7 –** So has anyone else had, a similar or a different experience…going to the doctors.

**P1 –** Right I’m off. I’ve got rid of them all. Okay.

**P2 (female) –** [name].

**P1 –** You received a letter when you went to the doctors, didn’t ya?

**P3 –** Yeah.

**P1 –** Were all an easy read.

**P3 –** Yeah.

**P1 –** But the pictures, the pictures you could tell that they got them from google.

**Interviewer –** Right.

**P1 –** There’s a, there’s no a, a website for easy read documents to be made. It’s called Easy on the Eye.

**Interviewer? –** Easy on the Eye.

**P1 –** And when [name] got this letter, it told her, it told her when her appointment was, but the pictures, you could tell it were a last minute made up thing. Urm, but it did tell you where to go for your appointment, didn’t it? And what time to be there, the pictures were just… like I’ll just google this picture.

**Interviewer –** Yeah, they’d not thought how…

**P1 –** Yeah, they didn’t…

**Interviewer –** They’d not thought it through.

**P1 –** No, that I don’t think they’d had the training. I don’t think they’ve just been told they have to… have to have it in an easy read format but I didn’t think they’d understood how to actually do it.

**Interviewer –** When you got that letter, did you like being able to have it in an easy read format?

**P3 –** Yeah.

**Interviewer –** And how does that…

**P3 –** When we went to the doctors at the time I told them it wasn’t even that easy on the eye and I, I don’t think there was any training around that.

**Interviewer –** So how do you feel generally about going to the doctors… the GP. Let’s think about if you’re going to the GP. Is it generally good?

**P7 –** Yeah.

**Interviewer –** So tell me why it is good, why is it good?

**P1 –** Put your phone away.

**Interviewer –** What do they do to make it good for you…

**P4 (male) –** Yeah, I am, I was gonna, I’ve been in hospital.

**Interviewer –** Yep, you’ve been in hospital.

**P4 –** Yeah for the few days.

**Interviewer –** Yes and how was that…

**P4 –** And my experience was alright. But some didn’t understand, but some did.

**Interviewer –** And what’s the difference in people that did understand you and the people that didn’t understand you?

**P4 –** I don’t know.

**Interviewer –** Okay.

**P4 –** Just some didn’t understand when I wanted something.

**Interviewer –** Okay. And if we could tell our students, tell our new doctors and new nurses, would it be… what would it… what would you want them to do? Would you want them to… talk to you more?

**P4 –** Yeah and understand…

**Interviewer –** To make sure that you can understand?

**P4 –** Yeah, what they’re doing or saying.

**Interviewer –** How does everyone else feel about that? Is that similar for everybody else? Do you want people to spend time with you explaining things to make sure you can understand?

**P3 –** Yeah I’ve got a question.

**Interviewer –** Go on.

**P3 –** Has doctors and nurses got our, our people with learning disabilities have got our own health passport?

**Interviewer –** Do doctors and nurses know that you’ve got your own health passport?

**P3 –** No, I didn’t say that, have they got it.

**P2 (female assistant) –** Have we got…

**P3 –** No, no I meant doctors and nurses.

**Interviewer –** Have doctors and nurses got their own health passport?

**P3 –** No, for people with disabilities is what I’m saying.

**Interviewer –** With learning disabilities.

**P2 –** So, you want to know if doctors and nurses know that people with learning disabilities have a health passport or if it can be made?

**P3 –** No, no I don’t mean that way, I’m saying, have they got it? Have they got the health passport?

**P2 –** Oh, have we got, like so, if you were to come into the hospital, would I have your health passport? Something like that, like…

**P3 –** No, well…

**P2 –** Yeah.

**P3 –** No I don’t mean, I don’t mean, I don’t mean you, I meant doctors and nurses.

**Interviewer –** Do the doctors and nurses at the hospital would they have it?

**P2 –** Oh, yeah, right would they have it, so the answer to that is that I’m not sure. We don’t, we haven’t learnt about that, so that’s a good…

**Interviewer –** It, I would expect that sometimes they might have your health passport. But sometimes…

**P3 –** If I, if I, if they got any…

**Interviewer –** …They might not.

**P1 –** Apparently, it’s supposed to be all online. Well, you print it all off, fill it all out then take it into the hospital.

**Interviewer –** When you go in.

**P1 –** When you go in.

**Interviewer –** Okay.

**P1 –** But yeah, that would mean then, next time you go in…

**Interviewer –** You’d have to do it all over again.

**P1 –** You’d have to do it all over again, yeah.

**Interviewer –** Okay so it’s about the hospital having an idea that you have done this once and you don’t want to do it another five times, six times…

**P3 –** And I, I, I, I, I, I don’t and that’s why I’m asking.

**P2 –** Yeah.

**Interviewer –** So it’s about continuity really.

**P2 –** Yeah, making it more accessible and… yeah.

**Interviewer –** That’s an interesting point actually. So that when people know you are coming, that you have special…

**P3 –** You see, I, I, sometimes I, I get an annual health check. And the reason behind why I have that is because I was gonna have asthma. I’m still coughing like I’m a gooden as it is now, that’s why I wanted to know if they have got a health passport there, for when I go to the GPs.

**Interviewer –** So when you do go to see the doctor or the nurse, what is the best thing that they can do for you. If you think, think in your head about a time when you went to see a doctor or nurse, it might be recently or a long time ago, it doesn’t matter, and it was really good and you felt that it was a really good appointment for you. What did the doctor or nurse do that helped you make it a good or a good experience for you? So think about it in terms about what they said to you or what it was like when you got there. Those sorts of things.

**P4 –** I’ve got...

**Interviewer –** Go on.

**P4 –** Went to the doctor and recommended me to go to hospital…

**Interviewer –** Yeah.

**P4 –** …then he helped me, get me admitted.

**Interviewer –** He helped to get you admitted, so you…

**P1 –** To the hospital.

**P4 –** Cos I had a stomach problem.

**Interviewer –** Yeah.

**P4 –** When I weren’t in hospital.

**Interviewer –** So he helped you…

**P4 –** Yeah.

**Interviewer –** …to get you admitted.

**P4 –** Yeah, he recommended to get it checked up.

**Interviewer –** Are you hiding from me? Are you hiding?

**P5 –** Are you hiding [name]?

**P1 –** Do you have a good experience when you go to the doctors [name]?

**P5 (older female assistant) –** Just keeps nodding her head.

**P1 –** What about you [name]? What is it like when you go to the doctors?

**P8 –** Mum takes me.

**P1 –** Your mum takes you.

**P8 –** Yeah.

**P1 –** And does the doctor speak to your mum or does the doctor speak to you?

**P8 –** Speaks to me and my mum.

**P1 –** Me, you and your mum.

**Interviewer –** And your mum… And is that good when the doctor speaks to you?

**P8 –** Yeah.

**Interviewer –** Yeah.

**P7 –** I feel like the doctor speaks to me and my mum as well.

**Interviewer –** Speaks to you and your mum.

**P7 –** Yeah. But my mum always asks to speak because, like, certain, there’s certain questions that I don’t know and my mum does more.

**Interviewer –** Yeah.

**P7 –** So, like, like, if I had like, like, I struggle, I fit like epilepsy, so I have to have medication. And the doctor, will, like, take like tell me them and then I’m going mum I don’t know what they’ve said, can you explain? And then she explains to me when your card just says, double check, just to see how things are going and that’s why I always have to take my mum with me, because I don’t like going on my own, because I don’t know what the doctors are saying to me.

**Interviewer –** So, well, maybe one of the things we can tell our new doctors and nurses is that, to speak to you but also make sure that your, anyone you bring with you understands as well so that they can help you if you want to know more afterwards. That…

**P7 –** Well my mum was, I had to go to the doctors on my own because I’d had a rea… a reaction of like spots on my arm and they said it was, I said what would it be and they said it, it’s to do with… I had it all over my body…

**Interviewer –** Yeah.

**P7 –** …Like all me body, all me legs, all me arms, all me back and they had to look and they said it was a reaction of, like, pets, something I’d be allergic to.

**Interviewer –** Animals, yeah.

**P7 –** But it were my mates, dogs, they must’ve had flees and it’s come to me and it’s bop up my legs. So I had to, I had to have cream and tablets, the tablets worked it’s all done now.

**Interviewer –** Yeah it’s all gone. Was it very itchy?

**P7 –** It was itchy all over and on me eye. But it were… I just, I told doctor and they said oh just, they just said it were just starting and they went thank you and so I went on my own and they explained…

**P (assistant) –** Oh God.

**P4 –** They broke the thermometer.

**Interviewer –** So if you can think of a, let’s think some more of a good time when you saw a doctor or a nurse, what else happened? How did that make you feel? How did they make you feel? Happy? Anything else?

**P1 –** Oh [name] what was it like for you when you go to the doctors. Who do you go with?

**P9 –** Mum.

**P1 –** Mum. And does the doctor speak to you or your mum?

**P9 –** Me. Sometimes my mum will like…

**P1 –** Ah, okay. And how do you feel when you go to the doctors?

**P9 –** Okay

**P1 –** You feel okay?

**P9 –** Yeah.

**P1 –** And, when you walk out of the doctors are you like happy with what they’ve said?

**P9 –** Yeah.

**Interviewer –** And when you, when the doctor speaks to you, what sort of things help you to understand what he’s saying? Does he, does he do anything special?

**P9 –** No.

**Interviewer –** Does he use big long words?

**P9 –** Just smaller things.

**Interviewer –** Yeah and helps you to understand. Does he want to know how you, what you think about things?

**P9 –** Yeah.

**Interviewer –** And so people were…when they go to the doctors they’re generally happy. How else does it make you feel afterwards? If you’ve been poorly and then you’ve had a chat to the doctor and they’ve said that things are okay, how do you feel after that?

**P1 –** So, we can talk about when you… when you broke your arm.

**P10 –** Oh yeah.

**P1 –** Did you, did you go, did you go to the hospital? Or did you go to the doctors first?

**P10 –** Urm, I went to see the doctor first. If I did break my arm. I explained what… what happened to my arm.

**P1 –** And then did they tell you to go to the hospital?

**P10 –** Yeah.

**P1 –** And then once they told you everything, how did you feel afterwards?

**P10 –** I weren’t in pain first…

**P1 –** Yeah

**P10 –** …and then, uh, it got worse.

**P1 –** Then it got worse? And when they told… when they put a pot on it…

**P10 –** Yeah.

**P1 –** Did they tell, did they give you any advice on what to do? With your arm in a pot?

**P10 –** They put the pot on (inaudible 18:01-18:15).

**P1 –** Yeah.

**P10 –** I were in pain, I couldn’t move me arm.

**P1 –** Yeah. So once, once you was at the hospital and they’d put a pot on your arm. After you’d come out, knowing, knowing what’s, knowing what happened, did you feel better for it afterwards, knowing that your arm’s going to be bit better?

**P10 –** Yeah.

**P1 –** Yeah.

**P10 –** Yeah.

**Interviewer –** When the… when the doctor was examining your arm? Did he… he or she explain you what he was doing whilst he was doing it? Did they tell you what they were going to do before they did stuff?

**P10 –** Yeah they did.

**Interviewer –** And did you have a special photograph of your arm taken, an X-ray?

**P10 –** Yeah I did.

**Interviewer –** And how was that? What was that like? Because you’ve met a really important type of healthcare professional called a radiographer. They take pictures of people’s bones. What was that like?

**P10 -** (Inaudible 19:27)

**Interviewer –** Yes.

**P? -** __(inaudible 19:43)__ your pain?

**P10 –** Yeah.

**Interviewer –** And do you think that people are able to explain or understand how to ask you about pain? Do you think doctors and nurses can understand how you feel about pain?

**P10 –** Not nice.

**Interviewer –** No it’s not nice.

**P10 –** I do know that by now, when I’m in bed, my feet get really stiff and I didn’t really what it was at first I was like oh I cannot walk on it and it were exactly like, it were like, it were like, the pain were like, worse than toothache.

**Interviewer –** Did you go to the doctors for that pain?

**P10 –** I’ve been once to the doctors but they told me to, to rest it one time then the others…

**Interviewer –** So when someone asks you to do, if they can examine you or um talk to you about something, do they ask you before they do it?

**P10 –** Say again.

**Interviewer –** So when, if they, the doctor wants to examine you or take your X-ray or look at a part of your body do they ask you before they do it?

**P7 –** So when I broke my wrist, this wrist, I had to go to St George’s to, for an X-ray.

**Interviewer –** Yep.

**P7 –** And they said it were broke and I broke it in two parts. I broke my wrist when I were playing football, okay, and I say like I thought it weren’t broke and I got to St George’s to X-ray it and they sent me to the hospital and they said it were broke. So I had a pot put there, up me arm.

**Interviewer –** Right up your arm.

**P7 –** And it were half there.

**Interviewer –** And what, where’s, what was that like when they put the pot on? Were you frightened? Were you…

**P7 –** No well I was in a lot of pain it were like I couldn’t go out or get in the bath or get in the shower I had to like put my arm like that and like middle of night but when I were sleeping on the night give me tablets I’d take like paracetamol and me arm were so like aching like in pain I screamed for my mum like mum come look. It was so, so I had to go back to hospital or, I was coming into work the day but I didn’t, I couldn’t do owt so I just had to do till. But I had to now cos I’m in agony and pains and pains, I couldn’t even eat.

Interviewer – Oh is that before you had a pot on it?

**P7 –** Yeah.

**Interviewer –** Yeah.

**P7 –** And I don’t like putting a bag on it and I ripped it off and it were better but still it feels like still hurts a bit when I try to like clap like that since I (inaudible 23:11), but I had to go, it were like nine o’clock at night and me mam went oh I’m not taking you, went well it’s broken, it better not be broke, she were going on holiday with my nan, she went it better not be broke, I went it’s broke mum. For God’s sake alright. I was so in pain I went to go to bed I were like in agony.

**P3 –** Did you feel a bit sickly?

**P7 –** I felt sick yeah, yeah. I couldn’t eat or, or like I was sick. But yeah, it helped when I were at St, when I was at…

**P2 (female assistant) –** Did you find it easy to tell them how painful it was?

**P7 –** Yeah…

**P2 –** And they understood?

**P7 -** …they just had to like double some more painkillers I was just up all night with it and I couldn’t go to the shower, so I had to get one of the, you know the things, to put on, what was it called, them plastic things to put on your…

**P3 –** Plastic bags

**P7 –** Yeah, had to put on your bag…

**Interviewer –** When you’re in the shower

**P7 –** Yeah, that were, that were like really, I couldn’t eat for like two days, I were in bed most of it. I some painkillers with water and I were still sick.

**Interviewer –** So when you, let’s think about a time, if you can think in your head of a time when you went to the doctors or the hospital and they, it was a really good experience, I want to try and think about what makes it a really good experience, so that we can, tell our students to do that for you. So, if you had, when you, when you go, what is it about when you, when you go, what is the first thing that happens when you walk in through the door that makes it a good experience? Or what would, if you, if your experiences aren’t good, what would make it better for you?

**P7 –** I don’t know, trying to think.

**P2 –** It could be like the welcoming.

**P1 –** Who do you usually go see when you first go to the doctors?

**P7 –** Receptionist.

**P1 –** There you go.

**P7 –** They always say… can I, like help you, yeah just come for an appointment. Good, what’s your name and date of birth and you go thank you go and sit down and wait to call you. I said thank you very much mate and they’ll come out and say thank you for your helpful. And they are helpful receptionists. Well where I live it’s… I go to… and all the doctors are nice and nurses are there. And the receptionist is nice.

**Interviewer –** So do you think it helps that people know that you might… that you have a learning disability before you get there?

**P1 –** Is it supposed to be on their system? Is it supposed to be…

**Interviewer –** In theory…

**P1 -** …highlighted to say.

**Interviewer –** I think in general practice it’s highlighted, I think when you go to the hospital is when things are, not that it isn’t highlighted, but things are not as well put together. So do you find that going to your GP is fine generally, people, you know, see the same people don’t you, work in the same place. But you go to the hospital, do you find that more difficult experience? Do you, do you, so when you broke your arm, obviously you’re not going to see those people on a day to day basis, you’re going in because you’d had an accident, or it was an emergency situation. What about going to see doctors at the hospital, for other things over a long period of time? Do you see the same people? Do you see different people?

**P7 –** See different doctors. I see different doctors at the hospital.

**Interviewer –** Yep and how does that make you feel?

**P7 –** Okay, but just think it just, I think it’s just hard thing just to, I don’t know if I know, I would know what new doctor at the hospital who knows me more, don’t like messing about and that. That’s what I feel like because like…

**Interviewer –** So do you have to explain the same thing lots of times?

**P7 –** Yeah well my, well my mum does because I can’t like, like my mum go to me she go, she’ll come in and see doctor and they’ve got like my record or to see what and then they’ll say oh well you’ve got to go and have this scan on your bone, well you didn’t tell me this, this is what, my mum gets annoyed because it’s really, it upsets my mum, because they don’t…

**Interviewer –** And then does that upset you?

**P7 –** It upsets me yeah.

**Interviewer –** That your mums upset.

**P7 –** Yeah. I remember last time when my mum was upset, it wasn’t like a doctor but it wasn’t like a thing, like a centre and we went to go and see this thing, doctor thing, and as soon as she just burst into tears, I said what’s up mum, is it me, she said no it’s just, because everything was going on in my life and it just, it were just, it upset me and I had to like are you alright mum, she went, I give her a hug and I, and she just were crying. I don’t like it when she cries and I just give her a hug when she starts crying or owt or not. But, she were upset last night, not last night but she was just really upset and tired, but, she were just fed up because she just like, I said what’s up like, she said nowt I just don’t (inaudible 29:30-29:34) my mum and she goes to the doctors and she has to explain it all to the doctor and her experience at the doctors are just the same as what I’m experiencing, like wasting time with doctors. Go and see a doctor and just don’t… it’s always there, I’m always there for my mum.

**Interviewer –** I’m sure you are.

**P1 –** What about you [name]? When you were there in hospital for a few days, did you see different doctors every day? Or did you just have one doctor that you saw all the time?

**P4 –** Think I had different doctor and different…

**Interviewer –** And then, so you’re, one more main doctor and different people…

**P4 –** Yeah.

**Interviewer -** …who would come to see you during the day and at night? And how did you find that? Did you have to explain lots of things lots of times to them?

**P4 –** Yeah…

**Interviewer –** And was that, was that okay?

**P4 –** Yeah.

**Interviewer –** Or was that difficult for you?

**P4 –** That is okay.

**Interviewer –** And when you…. We want to make things better for people going to the doctors, going to the hospital, so we’ve talked about perhaps them knowing in advance, highlighting on their system that you’ve got a learning disability. How do you think that will help then? What things should they do for you knowing that? Do you think? What would help, what would help you if they knew that, what could they do for you?... Cos I’ve got some ideas but I don’t want to say those ideas and you just agree with me, anything that you like that people do for you? Do they write things down? Do they give you leaflets? Do they…

**P7 –** They give me like for the appointments when you come to reception they’re meant to say, like in a leaflet to see what time your appointment, what day and that

**Interviewer –** So you’ve got something that…

**P7 –** That’s information for me that I know what time my doctors is…

**Interviewer –** And where you’ve got to go.

**P7 –** Yeah. And that’s helped me and it’s helped my mum as well cos my mum takes me sometimes.

**Interviewer –** What else could the doctor do for you or the nurse do for you?

**P3 –** Sometimes I go to an asthma nurse, makes sure my chest is okay.

**Interviewer –** Makes sure your chest’s okay, yeah.

**P3 –** Only time I went to the doctors, I remember writing a (inaudible 32:47) I couldn’t stop coughing and I didn’t realise I had a chest infection and I went to the doctors and they said I take a lot of antibiotics, one lot didn’t let it go and I had a second lot to make it right and… well before I finished the second lot of medication, I had to go to hospital, that’s what they said to me, to go to hospital, to get an X-ray to see if there’s more infection inside ya. And that’s what happened. But I had to come into work, didn’t I? That’s all, and then I went to doctors, went to hospital afterwards.

**P1 –** After you’d finished here?

**P3 –** Before I came in…

**P1 –** Oh yeah, yeah.

**P3 –** …that morning. They don’t think I’m really thinking about my own health. But the problem is, I’m one of those people who likes to do something, do stuff keep me going but sometime you got to think about your health, like most of the time, haven’t ya? Get on with me work. I’m one of those people like to, I don’t like being at home, me (inaudible 34:38) I’m one of those people that likes to come into work and sort me self out.

**P7 –** Do you mean you get, yeah, you mean you get more tired…

**P3 –** All you do is worry.

**P7 –** Yeah you worry don’t ya?

**P3 –** I don’t feel, I don’t feel right in myself when I know that and that’s when you get poorlier and poorlier.

**P7 –** Yeah that’s why I (inaudible 35:01) when I’m poorly.

**Interviewer –** And do you think that the doctors and nurses that you go to see understand that? Are you able to, do they ask you about it?

**P3 –** You got to be careful how you do that because a lot of them, English is not their first language, when you go to doctors and nurses. Sometimes you can get doctors that are foreign. So, that’s the reason we started doing (inaudible 35:31) club in the first place.

**Interviewer –** So when you go, if you see a doctor who’s, where English might not be their first language, but you want to be able to understand them, do you find that difficult then?

**P3 –** I find it very difficult when somebody is foreign.

**Interviewer –** Yeah. And… to understand their accent and to understand what they’re saying to you?

**P3 –** See, the problem with me see, is I like to explain stuff. But sometimes people do rush me.

**Interviewer –** Okay.

**P3 –** I don’t like being rushed, because I feel flustered.

**Interviewer –** Yep. So what you’d like is for people to have time for you?

**P3 –** Yes.

**Interviewer –** How does anyone else feel about that?

**P5 (female assistant) –** [name] always hides (inaudible 36:19)

**Interviewer –** Do you like people to have time in the appointment so you don’t feel rushed?

**P7 –** Yeah.

**P1 –** What’s the time, time slots for people with disabilities when they go to the doctors?

**Interviewer –** I think, I think for people with learning disabilities they can, certainly in general practice and certainly with the GP’s that I’ve worked with, if they know that that patient is not an emergency and they’re being squeezed in, if they know they’re coming they get a double appointment. So they get extra time. But at the hospital, at [city] has an arrangement where they would have a double appointment. How the message gets through to clinic, and often I imagine if you’ve sat in a hospital clinic, you’ve waiting a long time, and sometimes it’s because people are very poorly and they’re sorting them out and sometimes it’s because they didn’t know they needed a double appointment and people have taken, needed extra time. So there are things, but one of the things is about making the system better so that it knows, and that’s something we can think about, that it knows that you need that extra time so that you can get the best care that you can. And that’s one of the things we need to think about, isn’t it? Making sure that you’ve got time to get care.

**P3 –** What’s happens if I have me own place and I’ve got no one to go with to the doctors and the nurses?

**Interviewer –** Say that again.

**P3 –** What happens to these people that won’t, have got no one to back you up?

**Interviewer –** Well I, I that’s, that’s, I think that’s a big problem for lots of people.

**P3 –** In this case, if I had me own place, that’s that’s I know that’s why I’m a bit worried about.

**P7 –** If you had your own place [name] and nobody to go with ya.

**P3 –** Yeah. And that means I’ve got to do it on me own then off me own back.

**Interviewer –** So perhaps it’s having doctors and nurses recognising how difficult that is for some people.

**P3 –** There must be a question in front of them doctors and nurses, are you able to go to housing? Or owt like that.

**Interviewer –** So you want them to ask you first, what…

**P3 –** Where you live.

**Interviewer –** Where you live, about your life, and how, how, how things are for you, okay. So that’s, that’s a really important question. That’s a really important question.

**P3 –** And what frightens me getting my own place in the first place, I am know, I know I am ready to do it, but I, when people say about behind the closed doors, that’s what frightens me.

**Interviewer –** So you’ve brought some really good points about time and about them asking questions. What do you think are the right questions that doctors should ask you to know about you?

**P3 –** To really know us?

**Interviewer –** To know about you and your life. Cos what I find with our students is they’re really keen to do the best for all of their patients, which is why we’re here, to know from you what it is you want…

**P1 –** Cos you have, they have medical history, don’t they? But then that’ll go back from when they first, when they’re really young to, to now. But then, like [name] says, is there any information, is there any information about who they live with and, I don’t know.

**Interviewer –** Who they support, who their support is.

**P1 –** Yeah, like, like for most people who, who, you know, on the system when it does highlight to say that person does a learning disability, well, who’s to know they have support.

**Interviewer –** Yeah. Yeah. And, and what sort of learning disability they have and how much they can understand, and actually when you don’t feel very well, you’re very vulnerable and you don’t, you need even more help to make decisions around how you are. And you might not make, you might need someone to be there for you to, to discuss it with you whilst you’re there.

**P1 –** What about, uh, when you’ve been to doctors and you get prescribed medication, tablets, when you get, when you get given tablets, when the doctor says you need to take these tablets, do you, do you guys, does everyone understand how many times you should take tablets for?

**P7 –** Yeah, yeah I know what, I’ve took my tablets for. Twice a day. At the morning and a tea time. And that’s for the epilepsy. So like last night, I were out, and I didn’t actually take my tablet, it was a mistake, so that’s when, I must’ve, yeah, I must’ve missed that tablet and I didn’t get back till like ten o’clock last night and I didn’t feel too good. But I took it when I got back, cos that’s when I have to, if I don’t miss it…

**Interviewer –** You take it, reg…proper times…

**P7 –** yeah, but I’ve not had like epilepsy fit, I ain’t had a fit for like… last time I had a fit was when I was a baby. It’s all under control and I’ve been through the hospital and I’ve been seen the psychologist, what is it, is it psychologist, is it a psychologist?

**Interviewer –** Well you might be a psychologist.

**P7 –** Yeah at the hospital, where it tells me my fits are under control, don’t have to have no more fits, cos they’re all under control. Just have to keep taking tablets.

**Interviewer –** Just keep taking the medicine and stop you having a fit.

**P7 –** If you’ve not missed, missed them all.

**P1 –** So would it, does every, so, would it be easy to have easy reads labelling on medication?

**P3 + Interviewer –** Yeah.

**P7 –** [name].

**P1 –** With nice easy read pictures?

**P3 + Interviewer –** Yeah.

**Interviewer –** Big, big writing so you recognise what it is?

**P3 –** Yeah and it’s bolder.

**P1 –** When you’re at the pharmacy and you go collect them

**P7 –** I can’t see…

**Interviewer –** Tiny label…

**P1 –** don’t they print it off for you and stick it on, don’t they, please take these two to three times a day or whatever.

**P7 –** And I can’t see that, I need it bigger.

**P1 –** And, and it’d be nice to…

**Interviewer –** It depends what it is, depends what it is…

**P3 –** Easy read now and we asked them, that, why is it not easy read these letters. Urm, that’s, they said to me that’s a good point, why weren’t we not doing that. So we decided to do it that way now, (inaudible 43:08).

**Interviewer –** So if the hospital gives you leaflets, having it in an easy read format would really help you?

**P7 –** I want the, do you know them, like what you get from chemists, poster about like, I want it like bigger, cos then I can write like what tablets I’m taking and see what…

**Interviewer –** So you can see it better?

**P7 –** Yeah, cos I don’t, I can’t see it so small. I’m like… letters like that on that need to be bigger…

**P3 –** I think we should have pictures on medication as well…

**P7 –** Yeah.

**P3 –** A lot of people might not be able to read.

**Interviewer –** Yep.

**P7 –** Yeah, I agree.

**Interviewer –** And I suppose, I suppose it’s keeping the pictures the same for the same medication, because sometimes medicines can be the same but they can look different. So having pictures on the front of your medicines so you know what it is.

**P3 –** It’s like, like I say in my prescription they have a little thing inside, I don’t know what they’re called, instructions about your medication…

**Interviewer –** Uh, yeah the…

**P3 –** Well they’re very, very small writing and they need to be a bit more bigger as pictures, in case…

**Interviewer –** So when the, they give you a leaflet with the medicine that comes, it’s like all the warnings and things that if you, if you have an allergic reaction this might happen, these are the common side effects, so you need those bigger.

**P3 –** Yeah.

**P1 –** Would that just that, well that’s a lot of work, int it?

**Interviewer –** It is.

**P1 –** It’s a lot of work, but then again…

**P3 –** It has to be done [name].

**P1 –** Of course it does.

**Interviewer –** But it makes, but I think if it makes you feel safer, I mean ultimately one of the most important things that we do, we work with and the people that I’m working with, [name], [name], [name] and [name], we, we, we have a special interest in patient safety. So in making things, not only better for patients, but safer for patients and you’ve hit on a really good point there about medicines. So when the writing is really small, and the leaflet isn’t good, what do you do then? What happens then?

**P3 –** Go back to the doctors…

**Interviewer –** Do you just not read it? Go back to the doctors.

**P7 –** I think I’d go back to the doctors and ask.

**P1 –** Or do you ask, do you ask with who you live with? Do you say…

**Interviewer –** Do you ask your mum to look at it for you?

**P7 –** I’ll ask me mum first, then I’ll ask me mum and then if my mums not there I’ll probably go to the doctors. Just say doctor can you explain what this is please.

**Interviewer –** And do you feel, you feel able to go and do that?

**P7 –** Yeah.

**Interviewer –** That’s really good, that’s really good.

**P7 –** If my mum goes away or something and I’m on my own, I’ve like had to go to appointments, I’ll just go to appointments on me own or I’ll have to wait till my mum gets back for appointment. Cos like if I’m going to, like, I don’t know, an appointment to see a nurse or doctor, I’m going to have to wait till my mum gets back, cos then that’s, I don’t like going on my own. And I don’t like travelling on my, that way, not cos, I don’t like going on my own, I like to go with my mum. But like if it’s doctors, I’ll just go on my own to see doctor.

**Interviewer –** That’s okay then.

**P7 –** Yeah, yeah it’s fine.

**P1 –** I also think, um, updating your medical information, for example when you go into doctors and they go, can you spend two minutes just sitting down here to tablet just updating all your details, I think for these guys, as well, that should be in an easy read format.

**Interviewer –** Format, yeah, to make it easy for you to be able to do…

**P1 –** Because I don’t know if you’ve ever been asked that where they’ve probably if they give you a tablet or anything like that, where they’ve said or give you a piece of paper…

**P7 –** Yeah, yeah, to write different medications, details that…

**P1 –** Details and…

**P7 –** And I don’t know what I’m doing and that’s why I need like my mum to know what…

**Interviewer –** To know what you’re doing and to support you with that. So perhaps it’s being able to give you that but to come back to it, or if you know that you’re going to be asked that you could think about it before you go.

**P7 –** Like, if they give me a piece of paper like that and I have to write it, I’m like… well you’ll have to explain it or I’m going to have to take it home and show it to my mum and see what she says because this ain’t right, cos this what, I can’t understand what even have to do.

**P1 –** See if it were in an easier read format then you’d be able to complete it.

**P7 –** Yeah, but not, not like, when it’s not that no…

**Interviewer –** So to try and make it easier for you and to understand that different people have different needs and abilities to do stuff.

**P1 –** And I think, not just for adults with learning disabilities, but if that came across for everybody then it’d be so much simpler.

**Interviewer –** Yeah, it would be because then you’d only have to do it once, wouldn’t you? And if it’s inclusive for everybody, then actually it does make it easier, having something, I know my mum’s eyesight is very poor at the moment and she, she, the smaller writing she’s like looking at it to try, try and make sure she takes the same, the right medicines.

**P7 –** It’s for my, like for my grandma, like my grandma’s really poorly and she can’t, she’ll go to the doctors and my mum has to take her and explain what medication she’s on and what she’s not allowed to have, like morphine on tablets, cos apparently the doctors have been giving her morphine tablets and it’s, it, it does, it makes her like really thin. Like when she was in hospital, this is what the morphine was, it upset me because I had to go see my gran in hospital and the morphine was, the doctors was at, I think it were at [a] hospital, and they, all the nurses were fine, and doctors, but it’s the one nurse who told my grandma not to give, me mum told and me uncle [name] said please do not give her morphine tablets and then they give her morphine and… she went loopy. I’m sorry but that was not my grandma.

**Interviewer –** My grandma.

**P7 –** Because my grandma has got…

**Interview –** But all medications have got side effects and some people who can take medicines and some people can’t because it makes you unwell…

**P7 –** No, but this one was more, it were worse because they give her like ten, twenty and she was and I’m going grandma where are you, she went who’s that, I went it’s me [name], go away I don’t want ya and that’s my own grandma, I don’t, why does she do this, because it’s with the…

**Interviewer –** With the medication…

**P7 –** Yeah. Sorry but that was medication but…

**P3 –** And, and also as well we’ve got to be careful, we’ve got to be careful with medication as well because some people might got allergic reactions with them, with those medications, you’ve got to make sure it’s, it’s, it’s good to and it’s the medication is good for that person.

**P2 (female assistant) –** I’ve got a question.

**Interviewer –** Go on.

**P2 –** Do people, because you just don’t know do you, do people learning disabilities get like a longer time if you go to the doctors or is it a...

**Interviewer –** Some, some, some appointments are longer…

**P2 –** Right.

**Interviewer –** And certainly within general practice they try to give people with learning disabilities a double appointment. If it’s an emergency appointment I don’t know if it is the same. There is something set up at [city] where they are meant to have a longer appointment. How often that happens, in reality, is, who knows. But, it, it, but I think part of it was that, some of the staff that I’ve spoke to about this knew that they’ve got double appointments and some of the staff didn’t. But then it’s also then it’s marrying up that longer appointment with the person with the learning disability and quite often those things aren’t, not…

**P3 –** It’s just for people with disabilities you’ve got to see that, you’ve got to think about other people as well, um, we’ve got to think about the, um, if they’ve got a, that’s why it’s important to have a health passport. In case you’ve got allergic reactions or owt like that, it’s be on that passport.

**Interviewer –** So that they, people know before you come that that’s what you, the healthcare is what you need.

**P2 –** Is there an easier way to book appointments, for people with disabilities? Because I find it difficult, does like everybody find it easy to make an appointment if you have to go to the doctors? Or do you find it difficult?

**P7 –** I find it difficult because, I don’t know why I can’t make an appointment, I make an appointment over the phone and then they say they haven’t got any appointments and have to come and travel all the way up there to doctors and then they do it again and they haven’t got any appointments and that’s like a waste of my time, like, well you should’ve just told me on the phone then.

**P2 –** Cos I find it difficult making appointments that’s why I asked.

**Interviewer –** I find it difficult making appointments

**P7 –** My mum does as well when she does it.

**P3 –** Yeah.

**P2 –** So you feel like there should be like an easier way to access…

**P7 –** Yeah.

**P3 –** I live with my sister, right now, and she always comes with me…

**Interviewer –** Yeah.

**P3 –** What happens is I, what happens is, I don’t have my sister anymore.

**P7 –** She means if she don’t have, means don’t have your sister…

**P3 –** If anything happened to my sister, that’s what I mean.

**P7 –** Yeah your sister. Yeah and you have to go, and you have to go to the doctors on your own, you, you don’t like going on your own.

**P3 –** Yeah, it’s not because I don’t want to be on my own…

**P7 –** It’s just hard for you…

**P3 –** It’s be hard for me because, it’s my sister int she?

**P7 –** Yeah. It’s like me, if my, any my family gone and it was like, I’d have to, I’ll, I’m struggling to go to doctors on my own…

**P3 –** Are you alright when I said that? [name].

**P7 –** You alright [name]?

**P3 –** Are you upset yeah? If I have, I’ll apologise right now. Alright?

**P7 –** He said okay. You alright [name]?

**P6 –** I just want… to put in now, I got, I got, I only got me mum… cos me dad, I lost fa… fa…

**P3 –** A member of your family.

**P6 –** I lost, I lost, my family all died. My dad, my, my brother and my sister because she had dementia.

**P3 –** What your sister?

**P6 –** I lost my sister yeah. So when I lost my dad.

**P3 –** (inaudible 55:11-55:16) And then when we got to his own room, he went. He had cancer.

**Interviewer –** So when we think about, things that we can change for healthcare, things that we can help you with, it’s about, you’ve mentioned things about having time, making things clear, yeah, trying to understand how you live, so asking questions about your life now, explaining things clearly, is there anything else you can think about going to the doctors that you want to tell me about today? Or going to the nurse, or going into hospital? Anything you want to share?

**P7 –** Can you think of owt [name]?

**P –** I can’t…

**P7 –** Can you think of owt [name]? Can you think of owt?

**P3 –** Not really. I said it all.

**P7 –** I said it all, I can’t think of owt.

**Interviewer –** I think you’ve done a fantastic job thank you so much, I’m going to press stop on my machines now.

**P7 –** Stop.

**Interviewer –** Thank you ever so much for your help.
