# Supplementary material for: Systemic safety inequities for people with learning disabilities: a qualitative integrative analysis of the experiences of English health and social care for people with learning disabilities, their families and carers
Source: Int J Equity Health. 2022 Jan 28;21:13. doi: 10.1186/s12939-021-01612-1 (PMC8795982; doi:10.1186/s12939-021-01612-1)
Supplement: Supplementary file 3 — Additional file 3. [file 12939_2021_1612_MOESM3_ESM.doc]

**Focus Group 1.**

**Interviewer –** I’m going to use two, I’ve got two really small devices.

**P1 –** Oh god.

**Interviewer –** So I can pick up everyone else

**P2 (female assistant) –** It’s like taking notes, pen and paper.

**Interviewer –** And it means that, it means I can capture exactly what you say rather than just writing down.

**P3 (female) –** You’re not allowed to say each other’s names are we?

**P1 –** Ooh no.

**Interviewer –** Well you can say whatever you like cos only I will hear this and then I will anonymise it. So no one else, no one else will hear it.

**P1 –** She’s going to write a book…

**Interviewer –** And I’ll, I’ll delete the recording so no one else will hear what you’re saying and it’ll just be on a print, it’ll just be printed out on a paper and your names won’t be there.

**P1 –** Oh, okay.

**P5 –** So you can say what you like, so.

**Interviewer –** And I won’t even say where I am or what part of, what town I’m in.

**P4 –** No swearing, the only person allowed to swear is me.

**P1 –** Oh yeah cos you’re the boss lady okay.

**P5 –** Are they operating.

**Interviewer –** We’re ready.

**P5 –** …are they on then?

**Interviewer –** Yep they’re on, we’re ready to go.

**P5 –** Well, one thing I want to ask then…

**Interviewer –** Yes.

**P5 –** What about mental issues?

**Interviewer –** Okay. What about…

**P5 –** The reason I ask is because, you know, with learning disabilities, learning difficulties there’s still a long way to go with those things but I feel mental issues is still a huge way off from support. The reason I say that is because I’m suffering from depression and after a good couple of years and a after a couple of weekends I hit my lowest point again where, no offence, I tried to… because I’d really just had enough with what is my family where they don’t think, that they don’t really look at how ill I am and they feel I’m just over reacting about things. My best friend gets it and [name] gets, like recently she’d helped me apply for a bus pass, I’ve been begging for a long time for a bus pass because I need to get out of my house. I can’t drive and considering these fits might possibly happen again, I don’t want to be the one to cause a god forbid accident, but then there’s like on the official, was it [a] website or national website.

**P3 (female) –** It’s at least, it’s a county council.

**P5 –** Yeah for that website then to apply for bus pass apparently if you willingly give up your driving license that won’t help towards (inaudible 1:23:43) you have to think it’s really unfair because I’m doing the safe thing by not driving but to help my kind of mental issue right now is to get out the house but there’s nothing like around [town] anymore everything, everyone is taken away and I feel [town] if anything is hugely diverse against things that are invisible or invisibly, not wrong, but different about society.

**P4 –** Can I just make a point [name], I think [name], would you have a conversation with [name] at…

**P2 –** About the bus pass? Yeah afterwards.

**P4 –** No, but yeah just afterwards. I’m just thinking [name] would probably be the best person to help you with that rather than…

**P3 –** avoiding it.

**P2 –** But my own…

**P4 –** No but, just, just, just whether there’s anything that we can think of.

(inaudible voices over each other 1:22:52)

**P2 –** Yes but my only experience…

**P5 –** In general about another thing with mental illness in general because I still feel it’s a huge thing that’s not being looked at and…

**P2 –** I think it’s widely acknowledged [name] that if you haven’t got a learning disability but you have autism and, we know that there is a higher rate of mental ill health for people with autism, anxiety and depression primarily, but you do fall through the gaps, there is no autism service at the moment. But you’re, so you’re absolutely right that needs to be addressed and acknowledged and also how that anxiety and depression will impact on you differently to someone that doesn’t have autism, that’s not recognised.

**P4 –** So it’s, [name] can signpost you to perhaps somebody that can…

**P2 –** I can try but there is, you’re, you’re absolutely right that there is a lack…

**P4 –** Well that’s something that we need, that’s something that we need to raise then.

**P2 –** Yeah.

**P3 –** I have a problem all the time with mental health, I don’t acknowledge it but sometimes it’s always there because everyone expects you whose someone with autism to live in a society where they want you to change, they don’t want to change to fit you in. But sometimes with all of my other health conditions as well, it’s not always possible. I’ve been lucky, I have [name], that I see every week and then I got pain management psychologist, which I was petrified to get, cos I had a big stigma because working with (inaudible 1:21:15) and seeing the psychologist about all the bad things that I see on Facebook about psychology, sort of do this to you, psychologists do this to you and psychologists (inaudible 1:21:02), I was petrified to go to see a psychologist, even though this psychologist was to do with, supposed to help me with pain. And they said they wanted to go, they wanted to help me to go because they said some of the pain that we have is connected to our head, that it connects to the other bits of our body and if we sort that, that, I can’t remember what they call it, if we sort that out then the other bits of our body might not hurt anymore, because that pain, we’ve talked about the other bits of our pain. I had to have, I was lucky because I have seen (inaudible 1:20:25). So, I have an autism that, I’m sorry but, they’re quite more advanced than a team of four people, which are people for learning disabilities and autism, but, they don’t, you don’t need to have, unlike [name], you, you can receive just if you’ve got autism there. So they actually came with me to my appointment with me.

**P4 –** Right shall we go back then, to talk about that. I mean that’s really good point for you, but…

**P6 –** I was just going to add to [name]’s point there that, about the psychologist and how you were afraid of going to the psychologist, [name], do you think you’d be more comfortable about going to the psychologist if it had been explained to you better…

**P3 –** No.

**P6 –** …in the first place? So if somebody had said to you, have you got any concerns about seeing the psychologist and talked to you better.

**P3 –** I mean, no…

**P6 –** It wouldn’t have helped?

**P3 –** No.

**P6 –** What would have helped?

**P4 –** (inaudible 1:19:25) we felt safe cos you knew she wouldn’t do anything.

**P2 –** Is this because of your, is this because of the way autism impacts on how you kind of process and understand or what information that you read?

**P3 –** Yeah it’s a bit of both, I’m still learning how, because I was only diagnosed with being, having autism last year in May, I’m still learning how autism, I understand what autism means, but I don’t understand how autism affects my everyday life.

**P4 –** And that’s what we were talking about earlier, that there’s not enough support for people is there really? To understand that you’ve got that diagnosis is there?

**P3 –** And I don’t, and there’s so many bad things which (inaudible 1:18:43) and everything about people getting locked up cos of mental health problems as well, so I am scared of the word psychology.

**P6 –** But that’s what I’m thinking about if, when they were referring you to the psychologist, if that person that was referring you understood autism better, they might be able to explain it in the first place.

**P3 –** I think she did, I think she does understand. I think she was, I think she was understand, but it’s in me own head.

**P6 –** But is there, but if the autism nurse had been there that would have helped?

**P3 –** She was there, she was there the whole time.

**P6 –** I know but you said it was helpful to have the nurses there, so what is it they would do…

**P3 –** Well, I think…

**P6 –** …that would help.

**P3 –** This is going to sound silly, but I felt like she was safeguarding me, like you would’ve done.

**P6 –** Right. But what we should have is the doctors and nurses themselves that are working with you to be able to do that rather than having the specialist there that has to do it.

**P5 –** Overall, we should be able to feel safe.

**P4 –** Yeah, you should, you should be able to feel safe.

**P2 –** Will you need the autism nurse to go with you next time?

**P3 –** Yeah.

**P2 –** Really?

**P3 –** Yeah.

**P2 –** Even though you’ve already met the psychologist?

**P3 –** I’m not going by myself. The problem is as well because my health is so complex, that I’m scared they’d treat, they’d try to trip me up. And I don’t wanna talk about, and the other thing is, I’m sorry to say this, I don’t want to talk about my past all the time.

**P2 –** Do you guys use health passports?

**P1 –** I don’t na.

**P3 –** I’ve got a new one now.

**P1 –** But I’m, I (inaudible 1:17:24)

**P4 –** Have you got a health passport?

**P5 –** No one’s ever taught me about it before until [name] the other day sent me an e-mail, so that’s all new to me.

**P4 –** Okay.

**P5 –** But then I had to agree with what [name]’s saying there about a year ago when I was waiting in, I think it was in A&E section to see someone for my depression, I had to wait a good seven odd hours in the end to past 2am. Now I get it when people with more physical life threatening injuries, but the biggest issue I had there was waiting all along with no one to talk to. So yeah I got really upset I started self-harming myself and all I remember was this nurse coming through, excuse me are you going to carry on doing that I will call the police and have you removed. Now that straight away felt even more depressing because it’s like I’m not being understood, you know, I get it, people have more life threatening injuries but why not put us in a quiet zone or somewhere where we’re going to feel more comfortable than having to feel scared about being in such a public place where, for me I felt like I’m going to be judged.

**P1 –** At the end of the day people have got the power over you [name] and [name], that’s what I was going to say. I don’t, I don’t mind talking about my past, I’m quite happy to talk about mt pass because the more you talk about you, the more professionals understand you than (inaudible 1:15:55) and she said I’m a social worker, what, tell me about you. How am I supposed to talk about myself then (inaudible 1:15:47) can go away and I know what the social can need. I know what you need, I know what you were crying…

**P2 –** We don’t always know what you need.

**P1 –** No but I’m, no but I’m only using that for an example, I’m only using that for the example because I, I, I, to be fair you’ve got excellent social workers but not everybody’s excellent in doing what their job. They need to know about you, they worry a bit about social care and I heard on the news recently that Boris Johnson is going to reform social care to make it better, so that’ll be interesting to do with your research. Well, if you want to change social care how is he going to make it interesting, how is he going to make it better?

**P3 –** Right, going back to, say places in [a city] hospital there is also an autism safe room, where people can go in A&E where it’s specially designed for autism and the bit about, you missed completely, why I have my autism nurse with me, it’s not about training the doctors, it’s about when we’re in hospital we need some time that’s one-to-one care even people like [name], we need some people that we know to sit with us all the time to make sure we’re okay, to reassure us because when we’re, I’ve, I’ve got a weird phobia of everything to do with hospitals, so sometimes we just need reassurance, even when, just like the people with learning difficulties, that would make us say that more why. So someone with, whose a familiar face that’s who I need, not, it doesn’t, no, it doesn’t, doctors and nurses keep changing shifts, they’re not familiar and that is scary.

**P4 –** But that’s not gonna, that’s not gonna change though is it I’m afraid but…

**P5 –** I agree with what [name]’s saying though, if you had a sort of team then at least you have some people who do understand you…

**P2 –** A team yeah.

**P3 –** But that, that has, can you just stop interrupting me please.

**P1 –** Now, now, now come on.

**P4 –** Let’s agree, one of the things, the rules for this group, isn’t it…

**P3 –** Well I’m sorry but every time I’ve been talking you’ve been interrupting me.

**P4 –** Well…

**P1 –** We’re helping you.

**P4 –** We’re helping you, we just…

**P3 –** Well you’re not.

**P1 –** I am.

**P4 –** Right, okay, let’s just calm down. Have two minutes please.

**P1 –** Tell what I was gonna (inaudible 1:13:31).

**P5 –** Can I please be excused, can I be excused?

**P4 –** Yeah.

**P1 –** Okay.

**P4 –** He needs to go outside for a bit.

**P1 –** (inaudible 1:13:25-1:13:18) and they did for children so they could go play in the children’s group when people go for appointments and stuff like that. So, we are getting there, we are getting there with the health service cos I think the important bit about this research, we need to look after the health service what we got.

**P6 –** And I think one of the biggest barriers that we’ve got is things like the quiet waiting area would be lovely, so in our A&E we’d love to have one, but actually if we…

**P4 –** You haven’t got, you haven’t got the space.

**Interviewer –** The space.

**P1 –** Oh they haven’t? I’m sorry, sorry I…

**P6 –** If we, if we, I suppose, have a quiet waiting area, we’d have to lose two cubicles in A&E.

**P1 –** How big are they?

**P6 –** Which means there’s another two people waiting all the time. So what we have to do is things like, if we, if someone, we know that someone needs somewhere quiet…

**Interviewer –** We try and make a trade…

**P6 –** We try and let them wait in a quiet waiting cubicle, one of the cubicles have got solid walls rather than curtains so we can ask them to go in there instead. But sometimes you can’t fix certain things…

**P2 –** I’ve just had a genius idea.

**P6 –** Go on.

**P2 –** So you know when you go to, I don’t know, somewhere like Ben and Jerry’s and they’re going your table’s not ready yet go and wait at the bar here’s a beeper thing.

**P6 –** Oh, I was desperate to get one of them.

**P2 –** So to have one of those, so someone could wait wherever they wanted to. They could wait in their car…

**P6 –** They’d be great for outpatients, I keep trying to get them but A&E you couldn’t have them.

**P1 –** Why couldn’t you have them?

**P6 –** We need people there.

**Interviewer –** Totally different subject, I know they’re an improvement project for children’s paediatric surgery where they use the beepers to call parents for, to recovery.

**P1 –** So, now well…

**P4 –** Can I just have a quick, sort of a mention, we’ve been talking about autism a lot, but I’m just thinking about people with more profound and complex needs.

**P6 –** I’ll check on [name].

**P4 –** There’s a lot of, obviously there’s lots of work that needs to be done to make that better for people with profound and complex needs.

**P1 –** I don’t, we, I know from this group ages ago, we used to have [name] and [name] they used to go and do, you know, (inaudible 1:11:18-16) chats and stuff like that. That’s what we did in the past and it got…

**P2 –** Are [name] and [name] self-advocates? (check)

**P1 –** Yeah. [name] and [name].

**P4 –** [name], [name], and…

**P1 –** [name] [name], urm, they did the (inaudible word 1:11:06) check before at (inaudible word1:11:04) and they weren’t on the ward and you know the communication book?

**P2 –** Yeah.

**P1 –** They looked at one of them and they said yeah that would be good you need to put that in the communication book, you need to put this in your communication book. I, the funny story about the communication book, I love this story, one of the patients decided to take it home with him, he didn’t want to leave it, he just wanted to take it home with him because he loved it that much.

**P4 –** We’ll talk to [name] in a minute about that because [name] will know, I know [name] will probably say that they still have communication books on the ward. But I think what happens is that they don’t get used, or rather they’re kept away and the staff have them, but then they’re not shared and it’s the Sam,e with the other thing that we were talking about. It’s about the hospital menus, where they’re there in a format that are easy for people to understand, because that’s not always the case is it.

**P1 –** No it isn’t the case.

**P2 –** I keep thinking about the health task group meeting that I attended a little while ago when I was (inaudible word 1:10:09) to talk about the communication books and, the hosp, actually the hospital passports and how some hospitals wouldn’t accept them if they originated from a different hospital where essentially it’s just information that’s useful to share, so…

**Interviewer –** Just, I was…

**P3 –** I found on the internet I didn’t type all of the passport, I found one for autism and I found this, this other one on the internet and it was connected to…

**P2 –** Oh I’ve not seen that one before

**P3 –** And it was cos, because I was working close to, it’s only, it’s (inaudible 1:09:34) anyway because I’ve got both of them from my autism nurse in these and this one’s called get, get, get me better and she’s noticed it hasn’t got a name on the top it’s just got NHS and it’s just like we’re the Sam,e.

**P2 –** So stop branding them just have them.

**Interviewer –** Just have them yes, so they’re not, they’re not, there’s, different with the different trusts and it’s got different trust name on it.

**P2 –** Umm, so you were talking about the health inequalities for people with learning disabilities and I’m assuming that this is the Leder research…

**Interviewer –** So some of the stuff around, from, from the Leder group.

**P2 –** Yeah and you guys have got an understanding of Leder…

**P1 –** Yeah.

**P4 –** We’re going to be running…

**P2 –** Is that the one in March?

**P1 –** In March.

**P4 –** Yeah end of March we’re going to be running a conference for people.

**P1 –** Oh wow.

**P2 –** So just that information for health professionals to know that and to know what the evidence says would really help hopefully to change some practice. Sometimes you need to have it in context don’t you.

**P6 –** But (inaudible 1:08:35) focused only on learning disabilities…

**P2 –** No, that’s true.

**P6 –** Doesn’t include autism. But it should.

**Interviewer –** But it should yeah.

**P2 –** But there is also some research that about that…

**Interviewer –** Well there’s some I mean there’s, I know, I know there is research that says people who have autism and a learning disability the, the mortality rate is even more poor.

**P1 –** There’s a lot of things we need to learn about the health service cos you talk (inaudible 1:08:09) can talk about eating disabilities as well. There’s a range of topics you can talk about.

**P3 –** Well I’m trying to, well so far they just, they seem, if you’re trying to get to hospital and (inaudible 1:07:56) your disabilities because you’re not saying.

**P1 –** [name] while you were out the room we was talking about the communication book.

**P6 –** Communication books?

**P1 –** Yeah.

**P2 –** In hospital, you remember we talked about them before and about how they don’t often get shared in the ward.

**P1 –** On the ward.

**P6 –** I think communication books are one of those things that aren’t used that often because people don’t need them that often, so they get forgotten about. But on certain wards, like our stroke ward, if you ask any nurse on our stroke ward where’s the communication book, they’ll know straight away, sort of people that have had strokes struggle to communicate if they use them. But on other wards, even people with learning disabilities, it’s not the majority of people that would use a communication book. So they’re one of those things that they’re always available, but people do forget about them if they don’t use them regularly.

**P1 –** Right.

**P6 –** And I think I’ve missed the bit about passports as well haven’t I?

**P1 –** You have.

**P6 –** You talked about (inaudible chatter 1:07:00)

**P3 –** No, I was just showing that you were (inaudible 1:06:57).

**P6 –** Have we said about how there’s different ones for different areas?

**P3 –** Yeah.

**P6 –** And that’s…

**P2 –** Oops sorry.

**Interviewer –** And that different places won’t you… it’s just, it is just information.

**P6 –** I’ve, I’ve never personally come across (inaudible 1:06:47).

**P2 –** Yeah well not (inaudible 1:06:46).

**P6 –** No, but they’ve, they’ve done what we did because they used to have [city] written all over it and we, we have the regular meeting where all the, all the nasal nurses regionally get together and I chaired ours and said look we’ve, I went to the regional health task group and they’d shared about how some hospitals weren’t accepting other hospitals passports, so we’ve done ours without a logo on. So that means they’ve done the Sam,e. So hopefully more people are going to start seeing that it’s the patient…

**Interviewer –** Well understanding and appreciating our…

**P4 –** They should be appreciated everywhere, shouldn’t they? If you’ve got a, if you’ve got some information, and hopefully [name] you’ve got that now. Have you got, did [name] give you…

**P3 –** I didn’t…

**P5 –** No she sent me an e-mail but didn’t…

**P3 –** You can have, you can have this one and I’ll take another one, here you go, here you go, that’s okay, it’s fine.

**P5 –** She simply e-mailed but unfortunately the link didn’t come on…

**P4 –** That that’s the Sam,e as me, she sent that to me, I’ve already mentioned that to you [name] this morning, didn’t I? But I just…(inaudible 1:05:53)

**P3 –** But then that’s the part again, it shouldn’t be up to me to do this and even though I’m a health task rep, it shouldn’t be up to me to research new information to find other information to help myself and to help others. It should be something that is circulated to me.

**P5 –** [name] just bringing up a fair point there because when you go to things like a doctor or so forth, they’re the people we rely on to help us, you know, so the end of the day it’s like I only found out about (inaudible 1:05:20) recently through Tesco because there was a few stores who’re now throwing these things out, and I go to this place called the Orb in [town] with activities for people with mental problems and so forth, and I said to my group look why don’t we do some sort of drama it’s called Cabaret, sorry, advertising sunflowers to do with Spring but also to raise awareness for this because things like such as buses they still have the old logos showing elderly people stickers people needing to sit so on and so forth. One of our songs such as me with anxiety, I prefer a single seat on my own rather, not next to some stranger, but again that’s one of the things I believe still needs pushing forwards because it’s overlooked when it comes to hidden disabilities that…

**P3 –** And the other thing is, no disrespect to [name] but he said one time look it up on the internet. I couldn’t do a google search to look up a passport. Now I was lucky that I rang [name] and then she would sort me in the end, but when I’m poorly so many things come up about autism, you don’t know what they is safe and what is not safe, what is right and what is not right. (inaudible 1:04:05) [name] can you send me the link and she (inaudible 1:04:02). I mean I can’t at the moment, this is how bad I am, I’ve got a laptop and it’s quite simple to use but I can’t use it, I have to use my ipad, that’s the only thing I can use and if somebody else gives me an ipad I can’t use it I can only use my ipad. All my settings are saved on my ipad.

**P5 –** I’m like [name] because I’ve got a very good laptop which I never use, only through my itunes, I start using my phone, but even, like you were saying there [name] about google, the problem is with online it’s not simplified for most of us, you know, because I need things simplified but when you click a simple question it comes up with various results and you’re thinking, what, what, what, it’s nothing related to what you’re typing in.

**P4 –** So really I think what we’re all saying is there needs to be a better understanding.

**P1 –** I think there needs to be a better way…

**P4 –** And have better, clearer information. Go on [name].

**P1 –** Sorry, I think there needs to be a better understanding about technology information, doctors, the public at large decide how to treat you. Like [name] says that he likes to sit by himself. If I was a bus driver and I knew [name]’s disability oh [name] right you come here mate, sit there, that’s your seat while you’re on the bus or whatever or if I was a train person, right [name] you’re getting on a train, right, you’re in carriage A, I’ll show you where your seat is, if you get in trouble just give us a shout, I’ll walk up and down the carriage so you, you know, you feel safe, and if he decides he needs all that but people who are not in the profession need to know, understand about our disabilities (inaudible 1:02:14) or whatever, mental health issues or stuff like that, I think it’s down to society to look after people and the unfortunate bit about society, people tend to forget people because we’re too busy looking after our own life, we’re all doing our own thing and that’s the sad bit about society.

**P2 –** Do you think that relates also to the subject of the research about healthcare professionals…

**P1 –** Yeah, I, yeah, yeah…

**P2 –** everyone’s doing their job and we’re all busy (inaudible 1:01:45).

**P1 –** You’re all busy, but you see, professionals that’re doing (inaudible 1:01:43) job, look after wellbeing of the patients or individual people. The health service only gets funded by the government, so the government needs to look after people with stuff like that and, you know, we need to look after the health service, so the professionals can look after the people who need that support, that treatment or look after the nurses, doctors who need the job and stuff like that and have better information so people who don’t understand English can understand what the doctors are saying, but you need to understand about (inaudible word 1:01:02) disabilities as well as all the other range of disabilities of more complex needs.

**Interviewer –** We’ve talked a lot about things that haven’t been done as well. So can you think of an experience when you’ve been to your GP or possibly...

**P3 –** Can I just say one more thing…

**P4 –** Let, let, let the researcher finish, go on [name].

**Interviewer –** And it was a good experience for you. What made it a good experience?

**P1 –** For me…

**P3 –** The doctors recently.

**Interviewer –** Okay.

**P3 –** The doctors when I was in hospital last time when I said I can’t speak to you when I have five doctors crowding me all the time.

**Interviewer –** And did they leave some of them?

**P3 –** They were not happy, they were like how can you tell us to do that. Yeah they stood outside, well they went somewhere else and then they drew a curtain around and also when they examined me I made sure because I was petrified that I was allowed someone in the room with me because a nurse or someone in the room with me and either we’re a problem with the lights and the lights of the room because I’ve got autism and I’d be like please these lights, I actually get headaches that make me have fits all the time. So once I laid back and everything I said sorry can you get me my glasses because I can’t lay back because that might have got… so they’ve passed me my glasses as well and made sure I got…

**Interviewer –** Coming in and out.

**P5 –** Oh I’ll go next.

**P1 –** I think for me, do you want to go first [name].

**P5 –** No I’ll let you go, it’s alright.

**P1 –** I think for me I got referred to [city] and you know where they put you through the machine to go and scan your body?

**Interviewer –** Yes.

**P1 –** And I, I, I did say, I did say to the nurse I can’t fit, no don’t wait (inaudible 59:13) but he gonna explain everything what’s going to happen before we actually do it and they said can you, can you, can you transfer yourself onto our trolley so we can slide you into the machine, can you do it for him, no not a problem, so they did it and then they said that we want you to press this button just as a test and if you panic then if you press that we know when to stop the actual scanning bit. So pretty good, we were talking all the time through the process and that was good. I think also for me, I think our ambulance service were pretty good because they explained something to me. When I went to the doctors they explained about my (inaudible 58:28) and they said that oh it’s an infection of the blood. So if you understand what doctors are saying because carers (inaudible 58:19) now I don’t want to affect you [name] what with you being an ex-carer, kids sit there and they just they, they sometimes you think while you been to healthcare trust, carers seem to take over and not ask anything so it’s down to doctors and me talking to doctors.

**P5 –** I have to agree, disagree with you there [name].

**P1 –** Don’t care.

**P5 –** I agree, agree what you’re saying there about carers because a lot of them were like that whereas we unfortunately, well I say unfortunately, I was once with one particular client and I did things her way no matter what, as long as they were not risky or anything like that, I did things her way because that’s how I believe a carer should be. Unfortunately company would quite happen that some of the methods that I was doing, although the client was in a mind of her own, she had her own likes and dislikes and I just simply followed through. But for what you’re saying there about, I’ll be honest with NHS doctors I feel like for the past two or so years I’ve just been moved from one lamppost to the next to the next and so on and so forth down to my mental depression. The only good thing I’ve had was back, I think it was about August, September last year when I was involved with the crisis team, this one person who was at work with me she made a care plan and she did it exactly the way I wanted AKA she asked me do I want it in words or pictures so I chose pictures because it’s more simplified and, you know, what I do, work with for, all it takes is one caring management team to say okay we’ll simplify this for you so you understand better because the end of the day I can work, I can do things but because of my kind of autism I’ve got to do things differently, I don’t always know how until I do it you see. Think of it as like an old fashioned watch, instead of the digital ones but the old classical clocky ones, if one little teeny weeny little wheel falls out of place, the whole thing breaks apart and that’s how my mind set works. But with that put back onto the case with that person in the crisis team by planning the way she did, that was the only thing that helped me there and then because she knew exactly what she was looking into and she seemed to understand where I was coming from about how I felt inside.

**P4 –** Thank you [name].

**P1 –** I think also would parents who have got a disability and they’ve got children who’ve got a disability or whatever, we need to make sure they understand the information as well.

**P4 –** Yeah so if the parents…

**P2 –** They need supporters really.

**P1 –** Yeah, yeah.

**P5 –** And that’s all (inaudible 55:34) because I wanted to be like that person who could help the other individual like the carer like I was the carer. I’m always the helping person because that’s the kind of person I am it’s like my sister for instance, you can’t, I can’t teach her what’s going on with me because she’s that kind of person that’s like nope don’t wanna hear it, nope you’re just going to ruin everything for everyone else and she won’t take it on board you see, whereas where I can put my voice through to help those individuals who need that little extra voice, I’ll do whatever I can because at the end of the day like I think it’s what you said earlier on Sam,, we are all human at the end of the day so why can’t we be treated as humans instead of an individual with a classification.

**P3 –** I’m a mum to a twenty two year old and when he was born because I didn’t know about my autism then and I tried to get help and they thought I had post mental, post traumatic, what is it, baby depression yeah baby depression instead and I think it was actually, I had a really bad pregnancy and a really bad labour and then epilepsy started soon after he was born again. I think it was more a cry for help that now I’ve got this like vulnerable child thing it’s like what’re you supposed to do with it.

**P2 –** That’s a really interesting point you’ve made there though. As an autistic woman who has gone through her pregnancy and birth and what was that experience like, because I’m like, it’s quite limited, the materials available to me about that.

**P3 –** Well yeah because, yeah well it was different to what [name]’s told me because it…

**P2 –** What was your experience of pregnancy [name]?

**P3 –** No no what... no sorry what [name] said is that we did a thing on it that when, if people have, get pregnant in England they’re closely monitored by, by you and by the healthcare professionals aren’t they… (inaudible 53:30).

**P2 –** Were you not in England then?

**P6 –** It’s getting better as time goes on. So twenty odd years ago it would be very different.

**P3 –** Yeah. So I gave birth actually in Malaysia.

**P2 –** Oh right.

**P3 –** I was lucky because I had private health insurance.

**P2 –** You were very lucky.

**P3 –** So, the person that was with me was, I saw the Sam,e person all the way through before I was pregnant, whilst I was pregnant and then after he helped me give birth and then his wife was a renowned breast feeding person then I came back to England with this tiny child not really finding my feet, probably still got baby brain at the age of, when he was two month old. So it has been quite challenging at times, and the most challenging thing was really, it wasn’t actually ringing them up all the time and stuff like that, I’m scared of driving him, because I was… I used to have fits, so I was petrified of having fits, I was petrified of having things, more than anything else. I managed when he was younger when we lived in [city], but we moved here when he was four we went back to a load of social groups and everything, so I made sure I was around people all the time, but when we were at home that’s where I was vulnerable. There was nothing, I tried to get help but there was nothing there that they would understand. They made me do parenting classes but they didn’t quite grasp what was wrong.

**P2 –** And it was before, I suppose, you knew yourself.

**P1 –** I think…

**P2 –** Thank you. [name]’s got something [name] wanted to say.

**P1 –** Yeah go on [name] then, go on.

**P5 –** Thank you. You mentioned you wanted to hear good things, here’s another one from me. I had a massive argument with my doctor because of my depression tablets so on and so forth months ago, there was one thing she did do right though I’ll give you credit for and that was forwarding me to this place called the Orb as I explained they are to help people with difficulties, mental disabilities, so on and so forth. If it weren’t for her pointing me in that direction, I wouldn’t have had someone to go to around house (check audio 51:03) where it tries to keep my mind quiet and gives me an activity to do cos you know it’s like over two years ago I used to do guitar lessons. I’ve now broadened it by doing a bit of art now, cabaret as well and you know the make-up experience recently they now want me to do some of their make-up for some of the cabarets plays they do. It’s given me some opportunity as a possible horizon to maybe look forward to so I could save, cos it came from my doctor originally (inaudible word 50:36) to them. That is one good thing because if it weren’t for her I wouldn’t be able to go through considering usually you have to pay for it, but because I was referred by my doctor then… sorry…

**P2 –** So, so, is that about your doctor knowing what’s available in the local community? What might be of support to you?

**P5 –** Not just necessarily with that but also she forwarded, she referred me sorry to them.

**Interviewer –** Referred you to them.

**P2 –** So she didn’t just say I know it’s there, but she actively…

**P5 –** No she, you know, she looked into it and said okay they do this and that and this and I said okay well maybe I could go and have a look and when I recently was still in care I decided to get myself an electric guitar because I love metal goth kind of music so I thought okay might give us a little hobby, gets me out the house and maybe I could learn something new at the Sam,e time and my key worker who was, who worked me for the two or so years he’s been a really great guy, you know, very understanding and then we’ve done a few songs here and there but… yeah without a doctor first referring me I wouldn’t have been able to even get somewhere because as I say in [town] there’s nothing else to really do really. The only thing I can do at the moment is go to the cinema but that’s only when I go to [city] because I get my transport paid for by this thing called acting towards inclusion, that’s due to end in March because I’m up to the year end now. Again, yeah I’ll pay the monthly fee for the cinema but it gives me the pleasure I need to stay out the house instead of feeling confined where I’m constantly being depressed. At least at the cinema I can go there and, what’s the word, escape.

**P4 –** Escape. I was going to say, I was at a meeting yesterday and I don’t know if this is in [town] cos this was in [town] about social prescribing. You can self-refer yourself to social prescribe at your GP surgery and they will actually do exactly what you’ve just been saying. They will help you to talk through what’s going on in your life and they will be able to help you to find something for you to do. So that might be something that anybody here could do if they want to do.

**P2 –** Possibly. I don’t know an awful lot about it.

**P5 –** (inaudible 48:28).

**P4 –** So if I could look into that and perhaps I could share that with you.

**P5 –** You could if you wanted to. I’m still waiting at the moment for this thing called a link worker.

**P4 –** That’s the, that is, that is with socially prescribing.

**P5 –** But I’m still (inaudible 48:14). Like I called my mum but unfortunately the person came in to see me two weeks ago tomorrow she was ill, so fair enough. With what happened on the weekend I called Monday saying look I’m really sorry I’m having a really like that’s why I took to self-harm so on and so forth. They are trying to get back in contact with me so unfortunately the person isn’t back yet.

**P4 –** Okay.

**P5 –** So it’s a waiting game.

**P3 –** It was… sorry. It was the young CAMHs team that helped me the most when [name] was a baby. When [name] was joining primary school it was a young carers team, until he left primary school and went to senior school he had a great one-to-one counsellor that he used to shadow him and talk to him and then if there was any problems report back to me, because he was actually, I mean, he could’ve had it all the way through his life but he was bullied cos he was, cos he had a carer because of someone there to support him because of me and so he stopped it and then it’s affected his mental health, you know. So, urm, it’s hard isn’t it because he found that he couldn’t talk to anyone about it, there was only certain people he could talk about it, about it as well because people didn’t understand.

**P1 –** I think what would be interesting, if we find out what people did round the world, different doctors how did they treat people in different ways in different countries and we could learn from them, they could learn from us. But also, we, well I didn’t do it, [name] did it, cos the 111 number you know where you phone the health service number, they did it with a, [name] did a video huddle out so people could phone in the 111 number, the out service and actually talk to, and I think that could be better by actually when people phone in they get specific questions, not hard ones. Something so they can understand it. Even for the carers, so they could understand the information what’s being asked or whatever.

**P4 –** Thank you [name]. Shall we have a break?

**P1 –** Oh yes.

**P4 –** I’m conscious that we’re a bit… we’ve talked quite a lot haven’t we?

**P1 –** We have.

**Interviewer –** We have talked a lot.

**P4 –** Shall we have a ten minute break?

**Interviewer –** Sounds good to me.

**P5 –** You need to pause that.

**Interviewer –** I’m gonna pause it yep.

**P2 –** [name].

**P4 –** Oh I knew someone was missing.

(inaudible chatter 45:36-45:29)

**Interviewer –** Okay, so you’ve…

**P5 –** Press the red one isn’t it?

**Interviewer –** Yeah you press the red one again that’s perfect, thank you. So you’ve told me a lot about your experiences and things that would help. Is there anything you want to say about what we could do as healthcare professionals to make a, your experience at your GP surgery or in the hospital better for you?

**P3 –** Yeah talk to one another.

**P4 –** (inaudible 45:00).

**P3 –** One thing that we haven’t talked about…

**Interviewer –** Nope.

**P3 -** …is reaching out to people with learning disabilities and autism is when you’re on the hospital ward, when you’re suddenly told that you can leave if you’re elderly they have a discharge nurse…

**Interviewer –** Yep.

**P3 –** …come to see you. But they just presume that we’re okay and we can manage but we can’t.

**P2 –** Well some people can (inaudible chatter 44:35).

**P3 –** Yeah but they should, yes but what I’m saying is they should treat us as an individual and ask us, and also if they’ve changed our medicines around, we’re not always conscious of the medicines and they’re giving us medicines at their times not the times we might usually take them. Then they’ll take back (inaudible 44:16).

**Interviewer –** And of course changes to your medicines are a safety…

**P3 –** And… and then… yeah

**Interviewer -** ….safety risk aren’t they.

**P3 –** And then, and then they don’t write down what medicines we took when and where and what the medicines are and I, most times I’ve had to ring up the ward and say when was the last time I’ve had my medicine because with some medicines, especially if you’re on opiates, or (inaudible word 43:53) co-codamol you have to go an X amount of hours before you take the next dose and if they’ve not told you when you’ve had your last dose and then suddenly they let you home or they’ve not wrote it down or not passed on the information to your carer, how are you supposed to know?

**P6 –** Can I just clarify about the discharge nurses, [name]? So the discharge nurses don’t see people because they’re elderly or because they’ve got autism, they’ll see people that have got a package of care…

**P3 –** Right.

**P6 -** …at home. So if it’s our own carers need to start again and what they need, if the carers are still the right carers.

**P3 –** Right.

**P6 –** So it’s not about age, it’s about that person supported in the community.

**P3 –** But then you go home and they give it to you at home and then they’re supposed to write to your GP about everything that’s just happened and only thing with the GP, you can’t get a GP appointment and you finally get a GP appointment and the GP goes I haven’t got that letter and, and then, and then, and then look at me, you… for me I always have a copy of the letter and say well this is what’s happened and then they have to take on the information from there.

**P6 –** So the discharge letters automatically go to the GP, for everybody. So what it is, is the GP will get hundreds and hundreds of discharge letters a week, so they don’t read them all, they just know there is a letter.

**P1 –** Right.

**P6 –** So when you go to see them…

**P2 –** Even if they did read them all they wouldn’t be able to absorb all that information, you know.

**P6 –** No.

**P1 –** You’d be brain dead.

**P6 –** So if you go to the GP, they will have one but they probably won’t have seen it, because they won’t have time to read all of them.

**Interviewer –** And they might read it while you’re there then.

**P3 –** Well, though I always read mine when I’m… but then it doesn’t help when all of a sudden you stop (inaudible 42:02) things and you, you (inaudible 42:00) a lot of my care now is in [town] but some is in [city], but the computers from [city] and [town] are not compatible…

**P2 –** They don’t make a match.

**Interviewer –** They don’t link.

**P3 –** So that is another reason why I’ve got to print a lot of my documents recently to give to one to the other.

**P5 –** [name] do you have anything to say?

**P1 –** I think what we need to learn and educate people is, not to use jargon, because you will go to the doctors or go on a ward and they are gonna use jargon but many people don’t understand jargon.

**P2 –** It’s a different language isn’t it?

**P1 –** It is. But luckily for me it (inaudible 41:21) experience with the health service I’ve found so far is pretty good. But also there, there was a bad thing what did happen to me. I went in cos of me leg, and the carer was trying to explain to this doctor, I don’t know what planet, doctor planet was on, he said you can’t gets, you can’t release [name] to go back home he’s poorly, his legs poorly, urm yeah he can he can go home. In fact I was back in by Saturday tea time and the doctors were going (inaudible 40:48) they said, it’s your fault you did this, no it’s your fault you sent me back home cos you was using your jargon, jargon of how you know. Cos I had a carer who used to be a nurse, she said you shouldn’t have been let home, the doctors did it wrong, that’s why you’re going back in. Oh… yeah, we see where you’re coming from. So, that’s the thing.

**P4 –** I think…

**P1 –** Go on.

**P4 –** What’s happened sometimes with people is that the doctors thinks the person they’re talking to understands everything that they’re saying, and that might be because they’re too nervous to say, or they want to get out the room and they just say yes I understand everything or they just completely don’t understand. But the doctor or the nurse or whoever thinks that person understands exactly…

**Interviewer –** Even if they say, do you understand, you just agree. Yeah.

**P4 –** And that’s, and then that’s the problem because the doctor thinks you’ve understood everything you’ve just said, the person goes home and then that’s, then they’re probably back in again.

**P1 –** Yes.

**Interviewer –** What do you think we could tell our healthcare professional students about that sort of communication? Do they need to ask lots of times? Do they need to check for clarification?

**P2 –** I think for different, yeah, to repeat back…

**Interviewer –** Repeat it back to you.

**P2 –** …in your own words.

**P5 –** Make it more available for what options are, maybe.

**P3 –** Well especially if someone has a hospital passport and you know that person has a learning difficulty or autism and with some hospitals that don’t flag it on your notes, with those people, take more time and get to know that person and try to adapt to that person using whatever language cos usually in these hospital passports it will say talk to us this way, we don’t like this, we like this. Try to use that as your advantage to how to communicate with us.

**P2 –** And that’s really important for everybody that’s in hospital.

**P6 –** I was just about to say though…

**P3 –** I, I…

**P2 –** It’s true though isn’t it?

**P6 –** Yes very true.

**Interviewer –** Because actually when you are in hospital, and you don’t feel well, you feel very vulnerable and…

**P3 –** And also…

**Interviewer –** Disempowered and how you would react when you felt well is very, very different to how you feel when you don’t feel well.

**P6 –** [name]’s point about jargon is really interesting because even as someone who works in a hospital, I’ll often read a discharge letter and when I’m reading it I’ve then got to go on the internet and…

**Interviewer –** To decipher it.

**P6 –** Google what it is. So if I don’t understand what it is…

**P2 –** So plain English please.

**P6 –** It’s not just people with hospital passports who have learning disabilities, it’s everybody.

**P3 –** And also, write a summary, maybe, of what’s wrong with us and what happened.

**P6 –** And then that should be on your discharge letter. You should be able to understand it and I think it’s not just talking to people it’s…

**P3 –** Somebody, some…

**P6 –** give them writing as well.

**P3 –** Some people don’t…

**Interviewer –** So that you can take it away and read it again when you’ve finished.

**P3 –** But, but, but some people don’t like looking at the discharge letter. I’m talking about the more personal letters to us saying like you have this problem, you’ve been did this, urm, you were on this medicine and these are the times you had it. So then you, then you can look back at that and that is like your plan then for when you’re out of hospital, cos people think the discharge letter’s a scary thing and they don’t, they, always, they don’t always look at the discharge letter.

**Interviewer –** I know they, I know of…

**P6 –** And I think that’s what needs to change, so everyone can understand their discharge letter.

**Interviewer –** Letter.

**P1 –** I think what, me next…

**Interviewer –** I know there’s a, there’s a project not centred around learning disability or autism but around people understanding what we’ve said to them in their consultations, when they talk to the doctors and nurses and it was a particular, I think it was urm, I think it was an oncology clinic that they were trialling this in because you get lots of complex information when you don’t feel well at a time of real, real stress. So they were trialling a way in which that you could, that you and the doctor and other healthcare professionals could write on a piece of paper so they would write in easy language, simple language what the, what we did today and then you could write any questions you wanted to ask for next time.

**P6 –** We, we’ve got a form that we use quite a lot by a person that used to attend the (inaudible 36:20) but has now left the area and she found it really useful cos it’s just an easy read form, one sheet of paper that says these are the three things that I want to get from this appointment and that gets given to the doctor and they have another form that they fill in that says this is what we discussed at your appointment so they can take that away with them.

(inaudible chatter 36:02)

**P2 –** I would like that please, for me.

**P3 –** Can I, can I have a copy of that?

**P2 –** Have a vague one for my own personal use.

**P1 –** I think what would help…

**P3 –** I’m next though…

**P1 –** So…

**P4 –** I’m sorry [name] was just about to say…

**P1 –** Go on [name] you go, you go.

**P3 –** Sorry.

**P5 –** Thank you. I think one thing that needs to be more noticed by professionals is the availability of services AKA anything that is going to help individual. For instance, I can’t remember what it was when I first met [name], but it was back to when I went to this thing called Autism Strategy. Now, I’d never heard of such thing before, the only way I found out about that was a poster being put up at the Orb. So why can’t things such as that be more widely spread so that we can get more people who want to go to these sort of things rather than just word of mouth, because we won’t know these things unless we hear about it.

**P2 –** What I often think would be really lovely and I think some places do, and we don’t in this local authority at the moment, but if we had an autism page, for example, on our website where anything that was relevant to somebody with autism, such as the passport there or the word bank if it was on there and then all, all that, all you would need to do is to, to find out about is that page and then everything else within that page has got everything that has got links to things that would make your life easier. We need to do this.

(inaudible chatter 34:25)

**P6 –** I think things like the autism strategy, we need to make sure other organisations are involved as well, cos in the hospital I don’t think anyone understands the autism strategy at all or what’s involved in it.

**P3 –** No and the other thing that really upset me about that, was because it’s connected to [county] council, (inaudible word 34:08) is also connected to [county] council. I went along and there was not any information about there about (inaudible word 34:02), about flying high, about safe places, about (inaudible 33:57)…

**P2 –** So it was a missed opportunity.

**P3 –** So it was a missed opportunity. Guess who had to hand out all the information.

**P4 –** Well it’s good for you though…

**P3 –** Yeah but again it shouldn’t be all in my hands.

**P5 –** [name] just chill, chill you’re (inaudible 33:41)

**P3 –** It’s not fair, it’s not fair that I have to do this, it’s not fair that I have to be there to hand out all the information all the time.

**P2 –** Shall we talk about this after?

**P5 –** Yeah because at the end of the day with no [name], I’m meeting you here because (inaudible 33:29) as well, this is the reason why I joined (inaudible word 33:25) so I could be another kind of voice like you. At the end of the day we’re all where we are to help each other as to prove who we are. But we also need to push out of the voice, so things are more available for everyone else, cos not rude, there’s bounds to be, I’m sure, loads of autistic people in [town] who maybe are too afraid to speak, who don’t know who to turn to. I’ve never known who to turn to before because no one’s ever shown me the way.

**P4 –** And that’s well, that’s really well said, thank you [name].

**P6 –** Groups like this are really good for that. So people like, say [name] represents this group at the hospital, at the quality forum. So you share all the information there don’t you.

**P4 –** And that’s what, that’s, that’s really, that’s what your role is like coming to this group, is by learning the information and then sharing that out and that’s really, really important.

**P5 –** And that’s what could rid of the curiosity to want to join thanks to [name] and all of you here as well so.

**P4 –** So you should be proud of all that you’ve achieved. I mean, you know, that, you’ve done some really, really good work.

**P3 –** I just went there because I wanted to learn about autism though.

**P4 –** Well, you, well you have…

**P6 –** You have.

**P4 –** and that’s brilliant.

**P6 –** And you’re a good representative of this (inaudible 32:20)

**P3 –** Yeah but I understand that but, what, I’m sorry but I just wanted a day off where I could just learn something about autism myself, me, but it didn’t work out that way.

(Inaudible chatter 32:08)

**P2 –** Was this in the autism strategy consultations that they were doing just before Christmas?

**P3 –** Yeah them ones (inaudible 32:04)

**P5 –** But, as I was saying, that was the first time I’d ever even heard of the autism strategy cos nothing has even been broadcasted about help or people who want to seek help with autism because at the end of the day autism is a huge spectrum, there’s too many statistics. Thank you.

**P1 –** It’s okay.

(Inaudible chatter 31:41)

**P5 –** They’re losing that because there’s too many versions of it and there isn’t enough to say okay maybe we can help you here, maybe we can point you in this direction so on and so forth. I, for me, do I want to learn about autism in a nice way of saying it, no I don’t because I can’t take it all in. But, for me as a person, I am, I work better when I physically see work with each individual because you get to know that individual for who they are, what their strengths are and as I’ve said about work before, if I go back to employment I would love for an employer to say okay you can’t do this, you can’t do that, we’ll give you this, can you do that instead? Work with our strengths, not force our weaknesses.

**P2 –** Nice (inaudible 30:49).

**P1 –** You know the thing we went to [name]? Last year, you know, the lions thing? Would that have (inaudible 30:41).

**P4 –** Oh you mean [town] and all of the lions that knew…

**P1 –** Yeah, would that help, would that help with the research?

**P4 –** Uhh, well I think [town] and (inaudible 30:33) was specifically just for [town] area, wasn’t it? About how we could make things better. It wasn’t based just on health, it was based on more on social care I think, wasn’t it? And how…

**P3 –** It was about making our health professionals…

**P1 –** Yeah but I mean would it, would it help by (inaudible word 30:18) the research?

**P4 –** I don’t know. I mean there’s, there may be some, some things there that we can learn from, but you’ve all given some really good, really good information today.

**Interviewer –** I mean, I was really struck at the start by when you said [name] that you wanted to leave the hospital because you didn’t feel safe here. I’ll say, what, what we want to do is educate our students so people don’t feel they want to leave because they don’t feel safe. I think that’s a really powerful message to take to them.

**P5 –** Well yeah because when you go to these places you, it’s like we’re trusting them with our lives…

**Interviewer –** Yeah, yeah and that’s the, that’s the Sam,e for anyone whoever you are when you go into that and you were unwell and you feel more vulnerable than you would normally that actually you are putting your trust into them.

**P1 –** I think what would help everybody in everyday life is (inaudible word 29:24) people and talking to each other so when you’re leaving hospital, you get to go, you get to tell social workers that patient’s leaving. So when there’s a follow up like you’re going back home, a (inaudible word 29:11) nurse gets know and that’s where the biggest, that’s where the big fall down is. You haven’t got your team of people communicating with each other. So I think you need to, we need to learn from that as well.

**P4 –** Can I just say one other thing? I mean obviously we had another member of this group who sadly died now, but he was in hospital and he had a hearing impairment and he, I remember him saying to me that the difficulty that he had was that he was, he would take his hearing aid out when he was say sleeping, resting and the doctors were talking with him and not realising he couldn’t hear anything.

**P2 –** And I think again I think that is across the board. I have recently been to a hospital in [county] to assess somebody and I was speaking with their named nurse who didn’t mention that at all that he had a hearing impairment, I picked that up from his notes and when I asked her where his hearing aid is, well I don’t know where it is. A completely lack of regard for those really basic communication tools.

**Interviewer –** There’s no, there is no way that that person would have been able to talk properly with you if they could not hear.

**P4 –** And I know with [name], when [name] went into hospital and I went to visit him, sorry [name]…

**P5 –** No.

**P4 –** And [name] you didn’t even have a radio because you didn’t know your radio didn’t work.

**P1 –** Didn’t work no.

**P4 –** This was before your time…

**P5 –** Sorry (inaudible 27:47)

**P4 –** This, he had, you know like you can get a radio can’t you?

**P3 –** You can’t now.

**P4 –** Can you not get a radio now?

**P1 –** You have to pay.

**P5 –** Oh those ones that are hearing aid kind of things. Oh right.

**P6 –** You can.

**P4 –** Can you?

**P6 –** Yeah.

**P4 –** Oh right.

**P2 –** Are we talking about a radio like radio 4 type radio?

**P1 –** (inaudible 27:32) radio.

**P4 –** Yeah and [name]’s radio didn’t work but nobody had told him or helped him, it was before bed time and try, I, didn’t want, you know…

**P6 –** There’s still the people that can’t get the radio.

**P4 –** I think for somebody like [name] who can’t see, especially…

**Interviewer –** Yes, that’s really important.

**P4 –** And also for, like we talked before about somebody who was, had autism as well, that didn’t know how, what they could do with their time because it’s, it must be quite difficult if you’re suddenly in, like, you know, with both of you, you know, with a diagnosis of autism that you’re in a bed all day amongst other people and how do you fill your time. I know you’re not well, I know you’re not well…

**P2 –** (inaudible 26:50)

**P4 –** But it’s got…

**P6 –** I always say if I’m in hospital I’m going to be bored out of my tree the whole time and that’s going to be the same for everybody.

**P4 –** Yeah, so, so that thinking about how we could be, how we could make that a better experience.

**P5 –** It’s, it’s more just about that though. It’s about people, each individual’s willingness to do it. For instance when I was in care, this one person I worked with she found it hard working with me but I still worked with her. Well, you know, I don’t want to say too much because I don’t want to go against (inaudible 26:18) kind of thing, but she had, urm, she suffered majorly from seizures, she barely walked, so I’d see her more wheelchair bound, her legs were very big because of that. I sorted things with her, she has such as set plan to how she wants her clothes put away certain ways cos she couldn’t do it herself. So I took everything on board, the rest of my staff, didn’t give a damn.

**P1 –** I know the feeling.

**P5 –** The amount of times I would, the amount of times I would go to her closet or drawers and see things just chucked in there, it boils me that much and as soon as I have a go at them they’re pulling me aside, even when I was in a nice tone, they pull me aside, oh you had a go about this again [name], you’re, you know, that’s enough [name]. I think, come on where is the passion, where is your drive to say I am here to help that individual, I’m not here for myself. You know, the one thing I can’t stand about any sort of care worker is them going there for the money cos, well to me it didn’t feel like a job it felt like a home.

**P1 –** You went…

**P2 –** So basically, sorry [name].

**P1 –** It’s okay. You went beyond what you should be doing. You made sure that person was having the life what she wanted, you, you didn’t…

**P5 –** Exactly, I didn’t do it for myself, I did it for her.

**P1 –** Yeah, well for me, and I’m not criticising anybody, you know, I think the care industry people just go for their money or, you know…

**P2 –** I don’t think it’s always the case.

**P1 –** No, no but I’m just saying in general people could…

**P4 –** You’ve not had a very good experience have you Sam,? So…

**P2 –** (inaudible chatter 24:46)

**P5 –** And I would see that cos that’s how I did it as a carer myself.

**P1 –** Well, I wouldn’t, no.

**P2 –** (inaudible 24:42)

**P5 –** Not to get too involved in a client. This one time I had to suggest to her, look do you fancy a trip to [city] dungeon because she’s from [city] so I asked her about [city] dungeons so on and so forth. Great day, she enjoys herself, I’m scared. I don’t do scary stuff because I’m (inaudible word 24:25). We walk back home, she’s in a good mood, put her in her bedroom, put a DVD on that she wanted, I then got pulled aside by my two managers, why’d you take her there [name]? Well, so I asked her…

**P1 –** Because she wanted.

**P5 –** Oh well sorry [name] that’s not your thing being goth and everything and I said to them how f’ing dare you, I gave the client the choice, I gave her the choice and I said do you want to try something different, you’re from [city], have you been to [city] dungeons and if so do you fancy [city] dungeons since we’re close to that? But when they said that, that really drove me insane because I did everything for her no matter what and I did things, like I say, going above and beyond that I probably shouldn’t have done, but I did it in the best interest of the client cos I saw how strong her personality was and I wanted her to bring it out herself.

**P1 –** I would’ve (inaudible 23:32) upset the bosses.

**P3 –** But it’s, it’s like what you were saying about the deaf people on more, on loads of occasions I’ve been in hospital and I’ve had people, I’ve got epilepsy but I’ve got partial stages of (inaudible 23:11) that sometimes it looks like I’m daydreaming and I’m zoning out for a bit and next minute I’m so scared people are like shouting at me in my face telling me to do things and I haven’t got the foggiest what’s happening.

**P2 –** Because you’ve zoned out.

**P3 –** Because I’ve zoned out and I don’t know I’ve done it and it’s, and then I end up going and then (inaudible 22:50) crying ward, like why are you crying. Stop shouting at me.

**P5 –** See that’s how my sister was with me on Saturday because the anxiety she saw as me just over reacting that I’m going to ruin my mum’s big massive party because I’m suffering the anxiety cos of how busy the crowd was but in her eyes I’m ruining everything she’s planned.

**P4 –** So it’s just stepping away from those people that are causing you that distress.

**P3 –** Oh I nearly collapsed when suddenly they had to help me to my bed because I can’t walk anymore it’s like…

**P2 –** What, what I’m hearing, as a professional listening to this, is about the importance of a person centred care pattern that this important information, and then for those professionals involved to read that care pack and understand…

**Interviewer –** And act on it.

**P1 –** Understand it.

**P2 –** So that’s not overly complicated. Have it in bullet points if that’s easier to digest but have those important factors and that, doesn’t that a lot of professionals, senior health care professionals, often…

**P5 –** You know what else is really important to say there because for me and old (inaudible 21:45) I couldn’t read this massive folder. It’s too many words to take in, but that’s why when I worked one-to-one with this one client I got to know her better and understood what she needed so on and so forth, by that way say bullet points, it’s so easy for people like me to have a bullet point, okay that makes more sense.

**P6 –** And that’s a hospital passport.

**P2 –** Except…

**P6 –** (inaudible 21:21) it exists.

**P1 –** The one thing that, the one thing I like about hospitals is you can people listen as well you can sit on a ward and…

**P4 –** But you can because you can, because you can’t see you’ve got that extra sense haven’t you?

**P1 –** Yeah.

**P4 –** Where you can just…

**P1 –** Yeah, I can, but it’s brilliant because you can listen to people conversations. You can listen to the nurses (inaudible 20:59) oh have you took this, have you been to the bathroom, oh have you had your (inaudible word 20:55) and then the worst bit is…

**P2 –** That is just what it’s like.

**P1 –** it’s really important, oh Mr, Mr whatever your name is I’ve come to take you down to x-ray, I’ve come to take you for this and I always remember I was in [town] hospital and this fellar came in because he had, he had, he had a funny turn and he came down to the hospital and all we had, how will I (inaudible 20:30) and he says and I said to him where are you from, oh I’m from, I’m from [city] but I’d rather, if I get poorly I come in town (inaudible 20:21) something posher.

**P3 –** The point I wanted to say, sorry.

**P1 –** It’s okay.

**P3 –** Is that, so some people like me, it’s not continuous, it can change within a second, my health is, it goes like that, like with my epilepsy, my anxiety, my autism, it’s not continuous. It’s like one minute you’re fit and then the one minute you’re not. It can change in a second so it’s a, it’s a, it’s not so easy to put all that on a passport.

**P2 –** No I know, but just appreciating that maybe you live with a high level of anxiety all the time so there’s, so a smaller figure can seem like having a disproportionate reaction you know.

**P3 –** Yeah but also if you suddenly start having fits as well and you’re zoning out or your pain levels spikes it’s because of my tummy, it’s, so like (inaudible 19:20)

**P5 –** [name]’s got a fair point there, you know, because I could easily go to a gig with all a thousand people because it’s the kind of music I listen to and I would cope with that, but that party full of about a hundred odd people who are complete strangers and all that kind of atmosphere, that’s where my anxiety just went through the roof and that’s new to me you see. But like you were saying [name], (inaudible word 18:58) [name]…

**P4 –** [name].

**P1 –** You can call me what you want mate.

**P5 –** I do apologise.

**P1 –** No I, I won’t take offence.

**P6 –** You can be me for the day [name], it’s fine.

**P5 –** You, you can’t see, you can hear. So bless you, I can see, but I don’t like to hear. So every time I’m waiting I always have to put my earphones on cos the only way I’m going to feel comfortable and not keep hearing (inaudible 18:36) it just becomes too much.

**P6 –** I was thinking that when [name] was talking about it, about how he can hear everything, he’s always entertaining. But for a lot of people…

**P4 –** It’s too much.

**P6 –** It wouldn’t be entertaining at all.

**P3 –** Oh no, especially not…

(inaudible chatter 18:24-18:22)

**P5 –** …just enough, there’s too many what’s going on at the same time it’s like, I think it’s also I kind of have like a sensory towards that as well as towards rain because I don’t like getting dripped on in clothes, so apparently that’s a sensory thing for me because like it’s taken all these years to finally be told that when I could’ve been told when I was younger.

**P2 –** And I work, I’ve worked with some people who absolutely cannot tolerate a shower for that reason and so a bath is their choice for attending to their hygiene, but what if there isn’t a bath available in the hospital or in a therapy setting.

**P1 –** The worst thing about hospital is I was on a ward and they have to give you a bed bath, you know like, you sit on the tray and they wash, I was washed down there or whatever and I was on another ward where the man he said right we’ll get you, we could get you in the shower, we could get you in the shower not a problem and we had, I actually wet this fellars shoes cos he had (inaudible 17:22), and I wet his shoes by accident, and the wards they came banging on the door, what you doing in the bathroom, [name]’s just wet me shoes by spraying the shower all up my (inaudible word 17:10) shoes.

**P2 –** (inaudible 17:08-17:06)

**P1 –** Ah well you shouldn’t wear your (inaudible word 17:05) shoes. Oh but, but I wanted to but I think the worst bit about hospital, well, there was one, I asked the nurse one day could, could I have the bed pan, hello no you can’t someone else has got it. So I said that, sorry to her, well get to before we’ll share it together.

**P4 –** That was something I shared with [name.] [name] was doing your job again, that there was an issue, there was only one bed pan.

**P6 –** Was it a commode?

**P1 –** No bed pan what you, you…

**P4 –** No, it was a bed pan, an actual bed pan on the ward there was only one.

**P1 –** Only one.

**P4 –** And somebody else was using it. Either that or there was something not…

**P2 –** It’s not the kind of thing you want to share is it?

**P6 –** No, they’re, they’re disposable (inaudible 16:26).

**P4 –** But I think it was at that time…

**P1 –** It was at that time.

**P3 –** I’ve actually got a phobia of hospital bathrooms, I mean closed spaces.

**P2 –** Very specific thing.

**P3 –** Well it’s because when my son was a baby and I was on the ward with my son when he was younger and I was trying to have a bath with him and I was, he was actually in with somebody on the ward at the time and I got into the bath first and somehow [name] (inaudible word 15:58) needs to get out of the bath and I started having a fit in the bath and the water was nearly (inaudible word 15:51) what’s going over my head. Luckily my husband, no no my son was banging on the door and screaming and the nurses just walked past. Luckily my husband (inaudible word 15:40) noticed something was wrong, banged into the door and pulled out the plug. So now (inaudible 15:34) in hospital, I can’t, if someone leaves me hidden in closed, cos (inaudible 15:28) and locks the door or I’ve got to lock the door, I freak.

**P4 –** So have you got something like that on your hospital passport?

**P3 –** I, I, I tried to tell them and…

**P4 –** But if it’s on your passport…

**P3 –** I haven’t yet cos (inaudible 15:15), but that is the reason why I and it goes back cos I get a sensory overload of that time, that, that event really did happen.

**P2 –** It’s actually quite a traumatic experience.

**P3 –** And then I’ve had to take my bath, now, I don’t have a bath anymore. I actually have a wet room now because of that reason.

**P4 –** Just think that…

**P6 –** One thing that I think’s really important to acknowledge as well is that if you are in the hospital, on that ward, on that day, there’s probably going to be five or six people that work with you, so, as much as we’re saying how hospital passport put it in there. The chances of all five or six people having read it, every day…

**P3 –** They haven’t read it, yeah that’s the problem that’s…

**P6 –** are small. So I think there needs to be somewhere where it’s on the person to say, I can’t do that, I don’t like that.

**P3 –** Yeah but that’s what we’re trying to do.

**P6 –** I know that a lot of people can’t do that and it’s really important that you have these, but I think also people need to also tell people what’s important to them to save time.

**P4 –** (inaudible 14:15)

**P2 –** Or a one page profile even just summarise those key points and actually…

**P5 –** No offense, cards, because of the day and age we’re in, why does it all have to be on paper, why not…

**P2 –** It doesn’t, you’re absolutely right.

**P4 –** (inaudible 14:03)

**P5 –** You know, yeah, a lot of us use ipads, phones these days, surely we should be allowed to have something that’s gonna work in our favour, cos some people do prefer written stuff, some (inaudible 13:49) easier to work on technology.

**P6 –** As, as much as I agree with you, in reality, if it’s on paper and it’s there, they’re more likely to read it than if it’s on a computer.

**P5 –** No, no I’m not saying just a computer. What if we had, if we have our own devices there then we could show them there and then.

**P6 –** You could absolutely do that.

**P4 –** (inaudible 13:31)

**P6 –** And you could say to people, please read that, and if you say to someone please read that before you come and speak to me, they should be doing that.

**P4 –** That would be brilliant.

**P5 –** First I’ve ever heard that.

**P2 –** But absolutely (inaudible 13:22) on a ward round. Read that, then (inaudible 13:20)

**P6 –** Yeah, if you read that first before speaking to me please.

**P1 –** I think technology…

**P6 –** And all the key information can be there, but the problem we have is, if you say please read that and it’s twenty five pages long, the reality, I know it’s not ideal but the reality would be that that wouldn’t be read by everyone that’s going to see you.

(inaudible chatter 13:00-12:54)

**P6 –** And I think, I think people should be empowered to do that, to say please do that.

**P3 –** That’s why people mistake me for like freaking out sometimes when I’m in the bathroom cos there was one time I was on the ward and I said can you stay by the door and they didn’t and I just, couldn’t get back to the bathroom and I couldn’t walk I needed help to get back and I just carried on freaking. That’s why they didn’t understand and then they thought I was going, I was like getting (inaudible 12:26) being horrible to them but they, I tried to explain to them the reasons why but they wouldn’t, they didn’t listen.

**P1 –** You know these passports [name]?

**P6 –** Yeah.

**P1 –** What we were talking about. Did they have, did they have them in all hospitals or just certain areas?

**P6 –** Um, most hospitals have a different version but all hospitals should be using hospital passports.

**P1 –** Do, so, my question is, you don’t, it’s not, it’s not a nasty one, why don’t they have a national passport for everybody in the health service, not [town], have a national one where everybody can follow it, the guidelines saying this is the annual passport blah blah blah blah blah. Why don’t we have a national one?

**P6 –** Various reasons. Firstly, I’ll see I completely agree with you and it would be a really good thing to have.

**P1 –** Yeah.

**P6 –** But also people can’t agree on what it would look like. So I’m very passionate about the (inaudible word 11:30) very good, but I know from speaking to staff in A&E they would’ve been through all that. The traffic light one that we’ve got, they know the red section (inaudible 11:21) profile is what they’ve got to read, so they’ll read those two pages, but they wouldn’t read the whole document.

**P4 –** Right, okay.

**P6 –** So, if there was a national one and they said we’re going to use that, I would be saying from [town], we don’t want to use that one because it doesn’t work for us. Because for me, and even the traffic light ones, nearly all traffic light ones that exist, cos there’s lots of versions, communication is in the amber section which is the things that you should know about me, and the red section is things that you must know about me. So, at our hospital we’ve put communication in the red section because you must know it. And so it’s little things like that that no, not everyone is going to agree with me and people have their own ideas of what’s right.

**P3 –** But is (inaudible 10:36) that we all agree on.

**P6 –** Good luck with that.

**P3 –** The next thing you ought to have to get from you guys to regularly talk together, wouldn’t you? Because that’s…

**P6 –** We, we do talk all the time…

**P3 –** I know but that’s, it’s not you guys I’m talking about other staff cos the, it, it’s out, you do an amazing job and all of the people like you in all the hospitals do an amazing job then it goes out of your hands, it’s you’ve got to get the staff to meet you as well. That’s what I meant.

**P6 –** And then that’s why I really like the traffic one because the staff in A&E they like it and if the staff in A&E like it and are gonna use it, that’s one of the big barriers crossed.

**P3 –** Okay. But can I ask about the autism one? Do people recognise the autism one because that’s a national one for the national autism society, so that’s a new one.

**P6 –** So in our, in our training that we have, we tell people there’s hundreds of different hospital passports, it doesn’t matter which one someone brings in, it’s important to read it.

**P2 –** It is a template (inaudible 09:37).

**P3 –** But the national, but the national autistic association have put that (inaudible 09:33).

**P6 –** Yep, so that’s this one. So we have this one in A&E to give out to people.

**P3 –** Yeah. Right.

**P6 –** But one of the biggest problems I think, might be jumping ahead now, is that if somebody comes to A&E, we already need this information. There’s not much point giving it to them after everything’s gone horribly wrong. It needs to be given out sort of through primary care, through social care so actually if you go to hospital take this with you and you don’t have that horrible experience that first time because then next time you go you’re gonna be even more scared because the first time went so wrong. So, I think as much as it important for A&E and the hospitals to give these out, I think it should be given out before they get to hospital.

**P5 –** I think, not being rude, in this day in society and age we live in, not being rude, we should probably have our own passport AKA one that is made specifically for our terms for the person we are, our knowledge so on and so forth, instead of having one national box. I mean, if I may, for me to see (inaudible word 08:23) support, I feel a little bit offended by, yes I would like some more support, but it’s like once again we’re being labelled such as people being labelled with dyslexia or with any other brand. I’d rather make my own passport say this is my name, these are the issues I have so maybe you can understand the key points I’ve made. They don’t have to be big or massive.

**P6 –** And you can do that. There’s nothing to stop you doing that.

**P5 –** Again, that’s new to me as well. You see I’ve not heard things.

**P6 –** The other one that we’ve got the learning disabilities one, it doesn’t say their learning disability anywhere on it. It doesn’t say [town] anywhere on it. If that worked for you, you could use that. If one that they use in [city]l works for you, you could use that, you could use any that is right for you. If that means making your own…

**P4 –** Or just try it, just try it, just write your own one. If you’re very creative and I guess you are.

**P5 –** To an extent.

**P4 –** Yeah, well you could do your own passport.

**P2 –** Or you could adapt…

**P1 –** You could adapt it, yeah.

**P4 –** Just, just say, yeah. Just say this is about me and have your picture on the front or whatever and just, you could really go to town with it and just…

**P2 –** I think we all need to be reminded…

**P5 –** This is what I mean this is like new to me because without hearing these things from all of you right here and right now, I wouldn’t have known anything about it when you would expect more things to be, not necessarily thrown out towards us, but knock knock try this.

**P2 –** I’ve taken that on board and I think there’s a lot more we can do to promote things, but I, I…

**P5 –** (inaudible 06:54)

**P2 –** But I also think we all at times need to be reminded that we are the masters of our own destiny and there is so much that we can control and we shouldn’t need permission for that. But sometimes we need reminding, that we are in charge of our own lives. If you wanna do something that’s not already out there…

**P1 –** Get on with it. If you wanna go parachute jumping, you go and do that then.

**P2 –** Let’s see what we can do to create, you know, I’m not the most creative person.

**P3 –** Bear in mind [name], with those passports you’re, they have depression side, but you can fill in whatever section you want to fill in and how you want to fill in so it’s still relevant information to you. So it’s still how you want to fill it in and so you can just use that as a guideline when you make your own because the good questions are quite good, and, and um sometimes I think we should take examples from all the hospitals as well cos like in [city] they’ve got, they’re also some (inaudible 05:55) there. They’ve actually learning difficulty and autism hub volunteers connected to them where they help the autism team.

**P4 –** But we did talk about that before. This is going back a long time now about…

**P3 –** No I’ve only just read it, researched it.

**P4 –** No I was just going to say you’ve reminded me of something we talked about a long time ago.

**P1 –** Very long time ago.

**P4 –** [name] you’ll probably remember this. But we’re talking about having like a buddy system so like people with learning disability or autism can support other people like you were just saying when somebody’s in hospital I’d go and visit them and just sit with them in (inaudible word 05:16) and help them to, you know, just go through that time.

**P2 –** (inaudible 05:13)

**P5 –** Why not? (inaudible 05:11)

**P4 –** And you would be brilliant at that, that [name] because you’ve already, you know, you’ve been and did that carer, carer role.

**P1 –** (Inaudible 05:05)

**P5 –** Just about me being cared over, cos the kind of personality I’ve got I’m born happy to do that, I would love to just sit and chat away someone or do something they would be entertained to do if it’s going to keep them in the happy zone because at the end of the day it’s like a better carer for instance all your staff would see my client as the person she is and don’t really take her likes or dislikes on, whereas me I did the complete opposite. For instance, she’s a huge fan of batman, so when I started bringing joker involved and Harley Quinn, she got so into it. That was her choice, whereas again, the other staff they did not see the light of that at all. They thought it was me making it as my own decision for my own personal preference so on and so forth, whereas it was completely the opposite why I was doing, I was doing it for her because at the end of the day each human has their own individuality.

**P3 –** There is (inaudible 04:09) screening about two year and (inaudible 04:07) long time but I don’t know if they’ve got anybody in particular connected to learning difficulties and autism.

**P6 –** Anyone can volunteer to work from hospital. (inaudible 03:57) anyone can.

**P3 –** But that I, the one that, one that was connected what I was talking about was a bit different than what I talked about before because they actually connected to the [city] autism, learning disability and autism team and they help them do the work and they actually paid money so that’s why.

**P6 –** Because they’re not volunteers though, they’re employees.

**P3 –** Though they paid a bit of money to actually help employ them and they’re the people who take part in some, some of the training for the hospital and also some of the videos that (inaudible 03:22)

**P2 –** Well that’s one of the things that I was thinking about, listening to you guys and how passionate you’ve got from the lived experience that you’ve got, incorporating that so experts by experience having the training would be invaluable.

**P4 –** It would be, it would be.

**P1 –** Well, me [name] ages ago and the team, we went to radio (inaudible 02:59) and we talking about disabilities and how we treat people and stuff like that and a lot of from the, matron, matron of the ward it were, she came and talked about what she does at Airedale hospital and we did jam, we used to have diversity group and we would have to go and do a performance on how people get treated in hospital and stuff like that.

**P5 –** I would love to do that because I love drama.

**P1 –** Yeah, but we unfortunately we don’t, we don’t do drama anymore now.

**P5 –** Damn.

**P1 –** Well, we used to do, we did all sorts. We went to [town] and we did like (inaudible 02:17) and stuff like that and people appreciated it cos they related to what we were trying to get across.

**P5 –** That’s why it’s just like where I go to the [city] club about trying to promote more about the sunflower lanyard because the only way to promote it is for people to see some sort of powerful message. Like one huge message I believe in, for me, is music. There were loads of songs I listen to but only so many few select ones I listen to the lyrics behind them have that message of understanding that speak for me whereas I can’t speak for myself. So, that’s what I said to the group at [city], if we want to do something to promote sunflower and things like that, we should do something together so that we can get that message out. Even if it’s like, they do this thing called cabaret, I think it’s like drama similar so to speak, you do something like that. That’s one way of promoting it, it’s one way of shouting a voice out because at the end of the day why should we as individuals why should we be ashamed of what we have, what classification we’ve got. We’re still human at the end of the day, we’re just different, everyone’s different anyway.

**P2 –** And it shouldn’t be ashamed…

**P1 –** You should always embrace life [name] and enjoy what you’ve got man.

**P4 –** So I think there’s some really powerful messages, I’m just trying to wrap it up because…

**Interviewer –** (inaudible 01:06-01:03) because you’ve got other things you need to do as well.

**P4 –** I am go, I just want to just go through just a few other bits before we go.

**P1 –** I thought we leave.

**Interviewer –** I really, I really appreciate all your experiences and you telling me about them too.

**P2 –** There is some professional capabilities for social workers are you aware? Professional capabilities framework, urm, for, and there are, there are two, there’s there’s the overarching one but then there’s the PCF’s for autism and PCF’s for the learning disabilities. So specific skills, urm, or developmental leads that are addressed through, that’s the kind of evidence that is part of your training.

**P6 –** Did you see the core confidence’s frameworks that we’ve got (inaudible 00:21)

**Interviewer –** I’ve got, I was gonna, I was gonna, I’ve got the health, I’ve got the core confidence’s for that, but not for social workers we haven’t.

**P2 –** But you might, there might be some (inaudible 00:14)

**Interviewer –** They do so then, and they do social work in the school of healthcare with social work students.

**P4 –** Thank you very much [researcher].

**Interviewer –** That’s it, lovely. Thank you so much.

**P1 –** You’re welcome, you’re welcome [reseracher]

**P2 –** I’ve fully enjoyed being part of that discussion.
